# Pyrazole-sulfonamide scaffold featuring dual-tail strategy as apoptosis inducers in colon cancer

**DOI:** 10.1038/s41598-023-32820-0

**Published:** 2023-04-08

**Authors:** Reham M. M. El-Hazek, Nashwa H. Zaher, Hagar E. S. Emam, Marwa G. El-Gazzar, Amira Khalil

**Affiliations:** 1 Drug Radiation Research Department, National Center for Radiation Research and Technology (NCRRT), Egyptian Atomic Energy Authority (EAEA), Cairo, 11787 Egypt; 2 Biomedical Research Division, Nawah Scientific, Cairo, Egypt; 3 Department of Pharmaceutical Chemistry, Faculty of Pharmacy, The British University in Egypt (BUE), El-Sherouk City, 11837 Egypt; 4 The Center for Drug Research and Development (CDRD), Faculty of Pharmacy, The British University in Egypt (BUE), El-Sherouk City, Cairo, 11837 Egypt

**Keywords:** Chemical biology, Drug discovery

## Abstract

Dual-tail strategy has been successfully utilized in the development of novel carbonic anhydrase IX (CA IX) inhibitors. Herein we adopted this approach in the design and synthesis of a series of novel pyridine sulfonamide-pyrazole hybrid scaffold mimicking dual-tail inhibitors of CA IX. A library of 15 compounds was synthesized and assessed for their potential cytotoxic effects against colorectal cancer cells. Compounds **3**, and **11** induced potential cytotoxic effects against the three cancer cell lines (HCT-116, HT-29, and SW-620) with IC_50_s’ of 45.88, 28.27, and 16.57 uM, 25.01, 8.99, and 3.27 µM, respectively. Both compounds induced cellular apoptosis on HCT-116 and SW-620 cells, while compound **3** induced necrosis as well. In addition, both compounds induced cell cycle arrest on G0/G1, and S phases. Also, compound **11** showed potential autophagy induction on both colon cancer cell lines (HCT-116, and HT-29), and a little bit on metastatic type. Both compounds were less cytotoxic than the reference drug on normal epithelial cell. The migration rates of HCT-116 and the metastatic one SW-620 were reduced by both compounds. Finally, molecular docking of compounds **3** and **11** into the active site of CA IX confirmed in vitro inhibitory activity for both compounds.

## Introduction

Colon cancer is widely spread type of cancer causing high rates of mortality and morbidity among all cancer tumours. Worldwide, it is estimated that colon cancer burden to increase by about 60% which could result in 2.2 million new cases and 1.1 million deaths by 2030^1^. Despite the discovery of several novel anticancer agents to target colon cancer, the 5-year survival rates for patient with colon cancer still very low, below 15%^2^. The main current treatment for colon cancer is surgery with or without chemotherapeutic agents^3^. 5-Fluorouracil (5-FU) remains the milestone treatment for colon cancer patients over the past years^4^. 5-FU is an antimetabolite cytotoxic drug that exerts its effect through its incorporation into DNA and RNA molecules, resulting in their misconfiguration and ultimately leading to cell death^5^. Due to the emergence of drug resistance, the use of 5-FU has been limited in clinical applications. In advanced colorectal cancer patients who were treated with 5-FU alone, the response rate is very poor, only 10–15%^6^, while when 5-FU was combined with other cytotoxic agents the response rates greatly improved to 40–50%^7^. Several studies have suggested that multiple factors may contribute to 5-FU resistance^8^, including alteration in drug influx and efflux, enhancement of drug inactivation, and drug target mutations^9^. Besides, due to the high cytotoxicity of 5-FU, its use is always accompanied with high range of side effects including cardiotoxicity^10^ and cognitive impairment^11^, among others. There are still huge efforts needed to be done in the area of discovering novel therapeutic agents with higher efficacy, less toxicity and minimal drug resistance for the treatment of colon cancer. The application of targeted therapy in the treatment of colon cancer has been considered a critical shift in this field.

Apoptosis is defined as programmed cell death, which gained a lot of interest from researchers and clinicians in the field of cancer treatment^12^. Dysfunction of apoptotic pathways has been contributed to both colorectal pathogenesis and treatment resistance^13^.

Apoptosis constitutes of two pathways; intrinsic and extrinsic pathways where intrinsic pathway is mainly being regulated through family members of B-cell lymphoma-2 (Bcl-2)^14^. Whereas, extrinsic pathway is started through activation of death receptors^15^. Resistance to cellular apoptosis is considered an important early event in cancer pathogenesis, which made targeting apoptosis a crucial aim in the current anticancer therapies^15^. Alongside apoptosis, autophagy plays very important role in cellular metabolism and homeostasis^16^. Autophagy is a normal degradation process which responds to stressful cellular conditions including starvation, organelle damage, and abnormal proteins^17^. The main advantage of the autophagic degradation pathway is to offer protection to the cells from misfolded proteins, guard the organelles from present toxins, preserve energy homeostasis and to assist cell survival^18^. Furthermore, it has been shown that modulation of autophagy pathways plays crucial roles in both promotion and suppression of different types of cancers^16^. Therefore, designing new therapeutic agents which target autophagy could be a potential hit in cancer chemotherapy^19–21^.

In addition to all the above mentioned facts, hypoxia-related acidosis has been shown to participate in many processes related to cancer progression including, cancer cell invasion, migration as well as metastasis which would make tumour management more challenging and complicated^22,23^. As a defence mechanism, tumour cells survive in these conditions through activation and over expression of pH regulating factors including hypoxia-inducible factor-1 (HIF-1)-dependant factors^24^. HIF-1 dependant factors include sodium bicarbonate transporters, monocarboxylate transporter-4 (MCT-4), carbonic anhydrase IX (CA IX), and sodium-proton exchangers^25^. CA IX has shown to exhibit crucial roles in survival, migration, adhesion and cell-signalling pathways of tumour cells^26,27^. The fact that normal cells show very low levels of CA IX, while it is overexpressed in cancer cells, has made CA IX a promising target in cancer chemotherapy.

**Figure 1:**
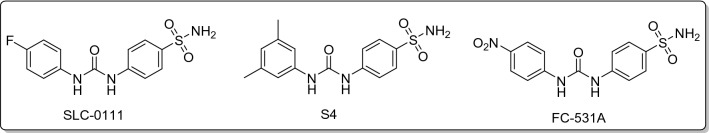
Reported sulfonamide-based small molecules with potent CA IX inhibition.

It is worth to mention that sulfonamide-based small molecules including SLC-0111, S4 and FC-531A (Fig. 1) have shown to exert potent CA IX inhibitory activities^28,29^. SLC-0111 is gaining lots of attention as it is now progressing into phase Ib/II clinical trials^30^.

As a continuation of our previous work^31^, we are encouraged to investigate the potential cytotoxic effects of 15 novel synthesised pyrazole-sulfonamides hybrids on human colorectal cancer cell lines (HCT-116, HT-29, and SW-620) using 5-FU as reference drug.

**Figure 2:**
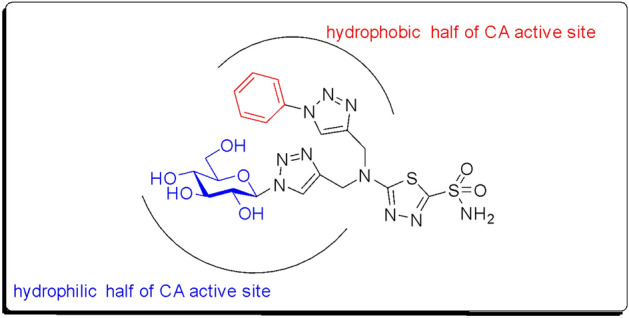
Dual tail concept in designing novel CA inhibitors^35^.

Many approaches have been applied to design more potent sulfonamide-based molecules as CA inhibitors. Among these approaches is the “tail approach” where it has been reported to be the most explored and efficient approach^32–34^. Furthermore, applying dual tail approach was employed by Tanpure et al.^35^ through combining the phenyl and glycosidic portions into the sulfonamide scaffold (Fig. 2).

**Figure 3:**
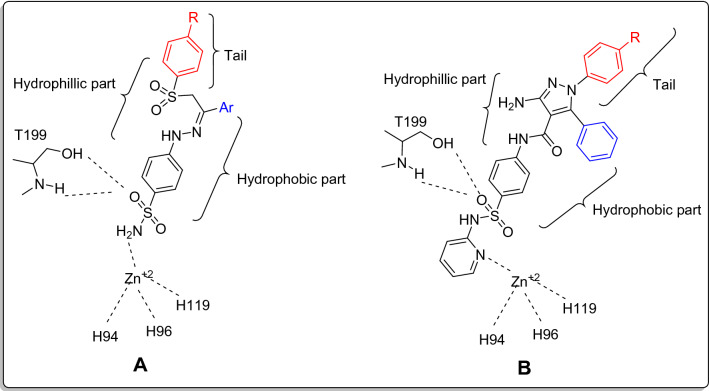
Applying the dual tail concept; (**A**) library designed in literature^35^, and (**B**) The designed target compounds.

In the present study, we were inspired by the work done in 2018^36^, as we applied the dual tail concept while designing our target compounds as shown in Fig. 3.

While designing our target compounds we have been also interested in investigating the use of pyrazole-sulfonamide hybrids as they were reported for their anticancer activities against colon cancer^37^. Pyrazole-containing molecules were reported for their potential anticancer activities through acting on various targets^38,39^. Thus, developing a scaffold hybridizing the dual-tail and pyrazole-sulfonamides might potentially lead to CA IX inhibition with augmented activity towards colon cancer.

## Results and discussion

### Chemistry

**Figure 4:**
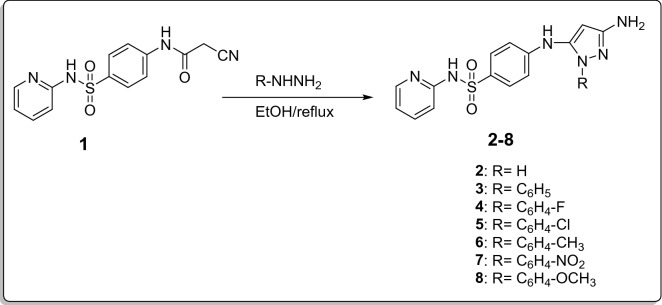
Synthetic route for compounds **2**–**8**.

Pyrazole-sulfonamide hybrids were reported for their anticancer activities against colon cancer^37^. In an attempt for obtaining new candidates to act against colon cancer, 2-Cyano-*N*-(4-(*N*-(pyridin-2-yl)sulfamoyl)phenyl)acetamide **(1**)^40^ was allowed to react with different hydrazines namely; hydrazine hydrate, phenyl hydrazine, *p*-flurophenyl hydrazine, *p*-chlorophenyl hydrazine, *p*-methyl phenyl hydrazine, *p*-nitro phenyl hydrazine and *p*-methoxy phenyl hydrazine in refluxing ethanol in order to obtain different amino pyrazole series of 4-((3-Amino-1-substituted-1*H*-pyrazol-5-yl)amino)-*N*-(pyridin-2-yl) benzenesulfonamide **2–8,** respectively, in good yields (Fig. 4).

Structures of all the newly synthesized compounds **2–8** were verified via spectral and elemental analysis. Disappearance of the characteristic C=O, CN and aliphatic CH_2_ signals of the starting compound **1** in all the spectral data. The introduction of NH_2_ group was confirmed through both IR, where new bands appeared, and ^1^H-NMR spectra, where singlet signal exchangeable with D_2_O at 4.62–4.73 ppm was displayed. Moreover, ^1^H-NMR spectra for compounds **2–8** showed singlet signals at 4.34–5.53 ppm attributed to the CH of the newly synthesized pyrazole ring. ^1^H-NMR spectra of compounds** 3**–**8** displayed extra aromatic protons at specified positions for the introduced phenyl ring. ^1^H-NMR spectra of compounds** 6** and **8** exhibited the most up field extra singlet signals at 2.25 and 3.43 ppm assigned for the introduced methyl and methoxy groups, respectively. Additionally, ^13^C-NMR spectra of compounds **2**–**8** showed up-field signals at 82.27–96.05 ppm, assigned for CH-pyrazole. Moreover, C-pyrazole was displayed at 140.06–140.87 ppm. ^13^C-NMR spectra of compounds **2**–**8** also displayed downfield signals at 153.51–155.06 ppm ascribed for the introduced C-NH_2_ of the formed pyrazole ring.

All the above mentioned, confirmed cyclo-condensation and reaction of different hydrazines at specified positions to form pyrazole ring.

**Figure 5:**
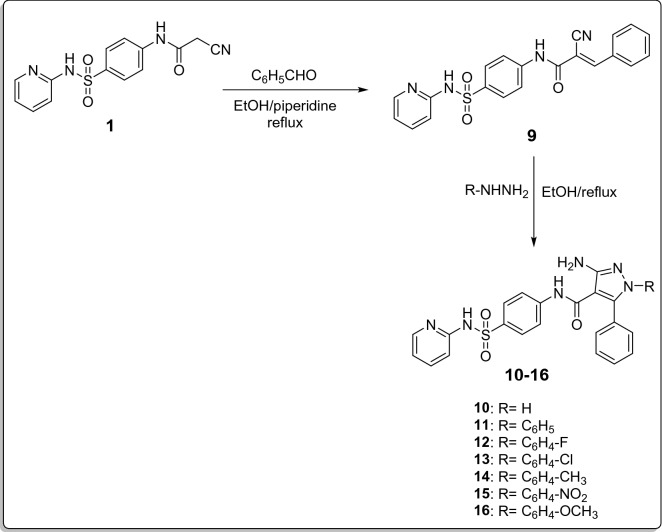
Synthetic route for compounds **9–16**.

A new series of phenyl amino-pyrazole derivatives **10–16** was synthesized by the reaction of the starting 2-Cyano-*N*-(4-(*N*-(pyridin-2-yl)sulfamoyl)phenyl)acetamide (**1)** with benzaldehyde in refluxing ethanol to afford 2-Cyano-3-phenyl-*N*-(4-(*N*-(pyridin-2-yl)sulfamoyl)phenyl acrylamide **(9)** with the elimination of H_2_O. Compound **9** was then reacted, with the same hydrazines mentioned earlier, in refluxing ethanol to give compounds **10–16** (Fig. 5).

Structures of all newly synthesized compounds **9–16** were verified via spectral and elemental analysis. IR spectrum of compound **9** showed characteristic band for CN at 2200 cm^−1^ and high intensity band at 1720 cm^−1^ ascribed for C=O, confirming the reaction of compound **1** with benzaldehyde at the specified position. ^1^H-NMR spectrum of compound **9** displayed singlet signal at 8.37 ppm attributed to CH methylene. ^13^C-NMR spectrum of compound **9** exhibited most shielded C atom of **C**=CH at 106.98 ppm and signal at 116.44 ppm ascribed for CN which both disappeared in ^13^C-NMR spectra of compounds **10–16**.

IR spectra of compounds **10–16** showed no bands for CN, at the same time stretching bands of the introduced NH_2_ appeared at the specified positions. ^1^H-NMR spectra of compounds **10–16** exhibited exchangeable signal with D_2_O at 4.84–5.23 ppm attributed to NH_2_. ^1^H-NMR spectra of compounds **11–16** displayed extra aromatic protons at specified positions for the introduced different aromatic hydrazines. ^13^C-NMR spectra of compounds **10–16** showed shielded C atoms for pyrazole **C**–C=O at 105.26–112.41 ppm, while C–NH_2_ appeared at 145.77–153.94 ppm. In addition, ^13^C-NMR spectra of compounds **10–16** displayed pyrazole **C**-phenyl at 145.12–145.76 ppm. ^13^C-NMR spectrum of compounds **14** and **16** exhibited the most up field signals at 21.13 and 56.04 ppm attributed to the introduced CH_3_ and OCH_3_, respectively. ^13^C-NMR spectrum of compound **14** showed a signal at 138.26 ppm ascribed for phenyl **C**-CH_3_. ^13^C-NMR spectrum of compound **16** displayed a signal at 158.70 ppm ascribed for phenyl **C**-OCH_3_. All the above mentioned confirmed the reaction of compound **9** with different hydrazines to form the pyrazole ring at specified positions in compounds **10–16**. Additionally, all mass spectra and elemental analysis come in accordance with postulated structures.

### Biological evaluation

#### Cytotoxicity

SRB assay was used to assess the cytotoxicity of fifteen compounds against three different colon cancer cell lines (HCT-116, HT-29, and SW-620) using 5-FU as reference drug. 5-FU remains the drug of choice for suppressing tumor in colon cancer patients. However, adjuvant chemotherapeutic agents of comparable potency are given to prevent recurrency^7^. The results of the tested compounds showed that only two among them responded to the three cell lines, while the rest were inactive. The phenyl pyrazolo derivative **3** and diphenyl pyrazole carboxamide derivative **11** were the most active with calculated IC_50_ ranging from 3.27 to 45.88 µM. The IC_50_ of compound **3** was significantly different from 5-FU on HCT-116 and HT-29 cell lines, while non-significantly different on SW-620 cell line. For compound **11**, the IC_50_ of was significantly different from 5-FU on HCT-116 and SW-620 cell lines, while non-significantly different on HT-29 cell line.

The obtained results suggests the importance of introducing phenyl ring to pyrazole and that substitution on phenyl ring causes drop in the activity may be due to the added steric effect. Accordingly, compounds **3** and **11** were selected for further flow cytometric studies (Table 1, Fig. S1).

**Table 1:** IC_50_ values (µM) for the 15 compounds compared to 5-FU on the three colon cancer cell lines.

Compound	HCT-116 (µM)	HT-29 (µM)	SW-620 (µM)
**2**	36.54 ± 1.35*	> 100	> 100
**3**	45.88 ± 1.24*	28.23 ± 0.97*	16.57 ± 0.83^NS^
**4**	> 100	> 100	> 100
**5**	> 100	> 100	> 100
**6**	> 100	> 100	> 100
**7**	> 100	> 100	> 100
**8**	> 100	> 100	> 100
**9**	> 100	> 100	> 100
**10**	> 100	> 100	> 100
**11**	25.01 ± 2.11*	8.99 ± 0.56^NS^	3.27 ± 0.33*
**12**	23.33 ± 2.23*	> 100	> 100
**13**	> 100	> 100	> 100
**14**	> 100	> 100	> 100
**15**	> 100	> 100	> 100
**16**	> 100	> 100	> 100
**5-FU**	3.7 ± 1.22	12.36 ± 0.76	14.80 ± 0.66

Results are average of triplicate experiments ± SEM.

*Significantly different from 5-FU; *NS* not significant, *p* < 0.05.

#### Apoptosis/necrosis assessment using flow cytometry

**Figure 6:**
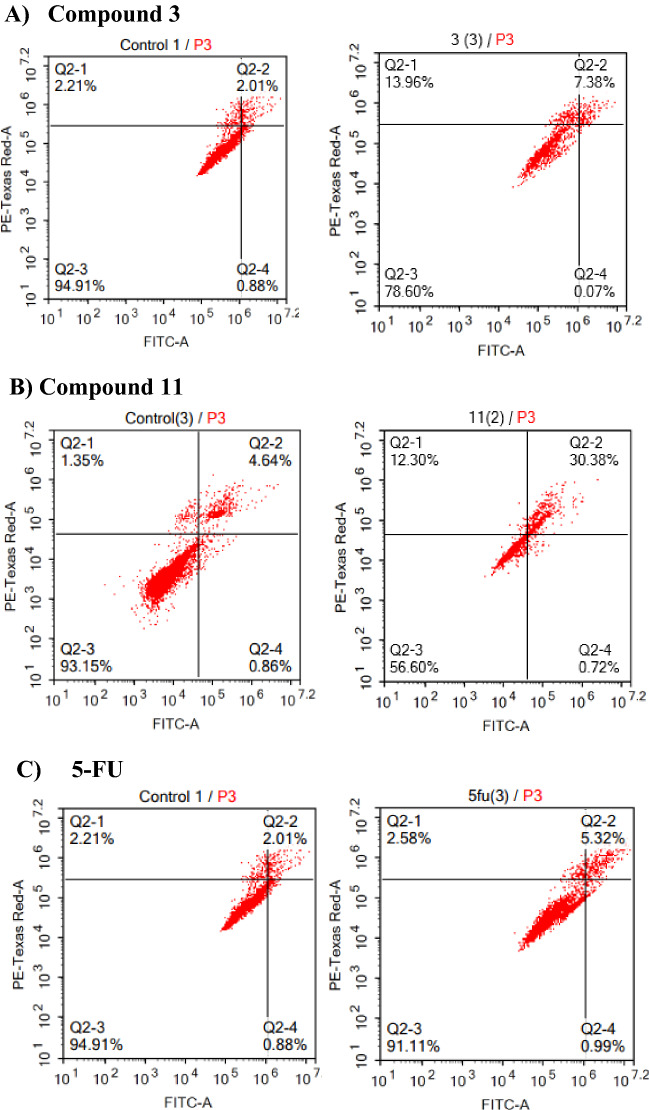
Apoptosis assessment for compounds **3**, **11** and **5-FU** on HCT-116 cells; (**A**), (**B**), and (**C**) respectively.

To determine the exact mechanism of cell death (programmed cell death versus non-programmed cell death), cells were assessed by Annexin-V/FITC staining coupled with flow cytometric analysis^41^ after exposure to the IC_50_ of compounds **3**, and **11**, and the positive control 5-FU (Fig. 6).

Regarding the HCT-116 cell line, we found that compound **11** displayed (43.40%) total cell death from the whole population (31.10% total apoptosis, and 12.30% necrosis). Compound **3** showed (21.41%) total cell death from the whole cell population (7.45% total apoptosis and 13.96% necrosis). In contrast, the positive control 5-FU which showed only (8.80%) total cell death (6.31% total apoptosis, and 2.58% necrosis) from the whole cell population. This means that both compounds elicit cell death by programmed cell death (apoptosis) mechanism on HCT-116 colon cancer cell line and they are more potent than the standard **5-FU**. Eliciting cell death by apoptosis has more advantages than necrosis as cells do not cause injury to neighbouring cells, in contrast, cells that go under necrosis cause potential damage to neighbouring cells, and potential inflammatory response^42^.

In addition to HCT-116 colon cells, we investigated the mechanism of cell death on HT-29 colon cancer cell lines. Compound **11** elicited (35.46%) cell death by the same mechanism as on HCT-116 which is apoptosis, but compound **3** elicited (10.83%) cell death by different mechanism (necrosis) from HCT-116, and the standard **5-FU** showed (11.54%) total cell death (9.52% total apoptosis, and 1.98% necrosis). From the data obtained, we can conclude that diphenyl pyrazole carboxamide derivative **11** is a very promising compound in the treatment of this type of colon cancer as it elicits cell death by apoptosis and is more potent than compound **3** and the positive control **5-FU**, Figure S2^43^.

We also investigated the effect of the two selected compounds (**3** and **11**) on metastatic colon cancer cells lines (SW-620) which showed a high percentage of cell death (70%) for compound **11** (16.72% total apoptosis, and 53.52% necrosis), and (59.83%) for compound **3** (16.72% total apoptosis, and 53.5% necrosis) compared to another two cell lines. In contrast to the positive control **5-FU** which demonstrated only 5.83% cell death (16.72% total apoptosis, and 53.54% necrosis) on this metastasis colon type, Fig. S3. From the obtained results, it is obvious that the phenyl pyrazolo derivative **3** and diphenyl pyrazole carboxamide derivative **11,** have the ability to target metastatic cancer type with higher potency than the standard treatment.

#### Cell cycle assessment

Further assessment for the mechanism of action of compounds **3**, **11** were performed in addition to cytotoxicity assay, and cell death mechanism, we analysed cell cycle for compounds **3**, **11**, against **5-FU** as positive control on the three colon cancer cells (HCT-116, HT-29, and SW-620).

**Figure 7:**
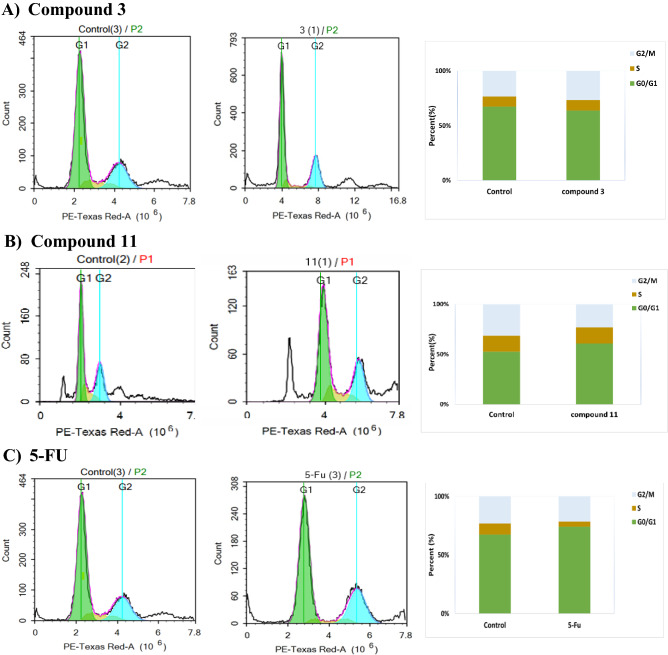
Cell cycle assessment for compounds **3**, **11** and **5-FU** on HCT-116 cells; (**A**), (**B**), and (**C**), respectively.

In regard to HCT-116: After 48 h of exposure**,** we found that compound **11** induced cell cycle arrest on G0/G1 phase by increasing its population from 36.26 to 45.39%. There is a decrease in G0/G1 of compound **3** (48.78%) compared to control cells (63.51%). Also, the positive control **5-FU** induced cell cycle arrest in the G2/M phase by (26.01%) compared to control cells (21%), Fig. 7^44^.

For HT-29, After 48 h of treatment, we found that compound **11** decreased the cell population from 99.59 to 63.86% in G0/G1 phase and induced antiproliferative effects through S-phase arrest by increasing its population from 10 to 15.54%. Also, compound **3** decreased cell population in G0/G1 phase from 74.34 to 66.03%. On the other hand, the positive control **5-FU** decreased the cell population in G0/G1 phase (81.45%) compared to the control (99.59%), Fig. S4. We can conclude that diphenyl pyrazole carboxamide derivative **11** affects DNA synthesis by cell cycle arrest in S-phase in contrast to the positive control **5-FU** which induced cell cycle arrest in G0/G1 phase^45^. The increase in the S phase of compound **11**-treated HT-29 cells, may be due to the induction of S phase arrest or the acceleration of the transition from G1 to S phase or both reasons^46^.

For the metastatic cancer cells SW-620, after 48 h of treatment, we found that compound **11** induced cell cycle arrest in G0/G1 phase by increasing its cell population from 32.06 to 50.47% and decreased the S phase population from 37.06 to 29.5%. While compound **3** has no effect on SW-620 cells, in comparison to **5-FU** the positive control which decreased the population % from 37.06 to 29.5% in S-phase, Fig. S5, from the obtained results, diphenyl pyrazole carboxamide derivative **11** has a promising effect on metastatic cancer cells.

#### Autophagy

Autophagy is an alternative programmed cell death pathway; however, its role in cancer cell death is complicated and often controversial^14^. Herein, we assessed autophagy using acridine orange lysosomal stain coupled with flow cytometric analysis.

**Figure 8:**
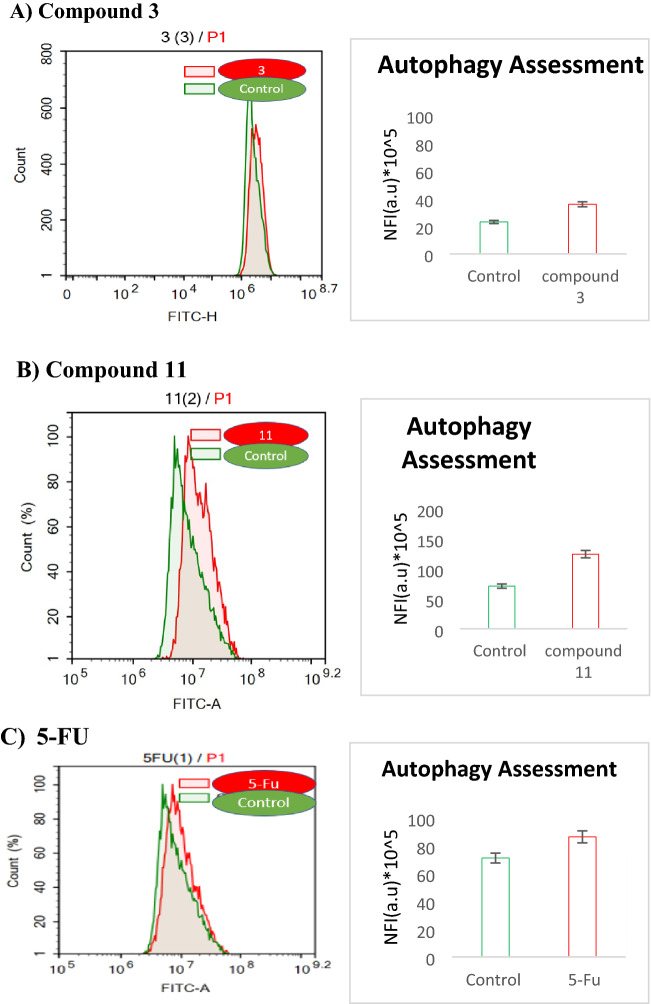
Autophagy assessment for compounds **3**, **11** and **5-FU** on HCT-116 cells; (**A**), (**B**), and (**C**), respectively.

For HCT-116, after 48 h of compounds exposure, phenyl pyrazolo derivative **3** induced autophagy in treated cells from (23 * 10^5^) to (36 * 10^5^), and diphenyl pyrazole carboxamide derivative **11** also increased autophagy from (71 * 10^5^) to (124 * 10^5^), and the positive control **5-FU** also induced autophagy from (71 * 10^5^) to (86 * 10^5^), Fig. 8. This means that the two compounds elicit cell death by autophagy mechanism and the most potent one was diphenyl pyrazolo carboxamide derivative **11**.

This confirms the ability of compound **11** to induce irreversible cell death by sensitizing cell autophagy after apoptosis^47^.

For HT-29, after 48 h of treatment, we found that compound **3** has been of no effect on autophagy, but compound **11** has induced autophagy in treated cells from (66 * 10^5^) to (85 * 10^5^) in comparison to **5-FU** which induced autophagy from (51 * 10^5^) to (94 * 10^5^) (Fig. S6).

For SW-620, after 48 h of treatment, we found that compound **11** induced autophagy in treated cells from (45 * 10^5^) to (55 * 10^5^), while compound **3** induced autophagy from (45 * 10^5^) to (57 * 10^5^) (Fig. S7).

#### Assessment of tumorigenicity selectivity

We tested also the cytotoxicity activity using SRB test on normal cells OEC (Oral Epithelial cells to understand the selectivity of these two compounds **3**, and **11** between tumorigenic and non-tumorigenic cell lines. The results showed that the tested compounds were much safer than 5-FU on normal epithelial cells with higher IC_50_. Compound **3** was ten-folds less cytotoxic than 5-FU. While, compound **11** was nearly 6-folds less cytotoxic than 5-FU. Their IC_50_ are (24.68 and 14.65 uM) compared to 5-FU which is 2.35 uM (Table 2, Fig. S8).

**Table 2:** IC_50_ values (µM) of compounds **3** and **11** on normal oral epithelial cells (OEC).

Compound	OEC (µM)
**3**	24.68 ± 0.31
**11**	14.65 ± 0.22
**5-FU**	2.35 ± 0.30

Results are average of triplicate experiments ± SEM.

#### Cell migration assay

**Figure 9:**
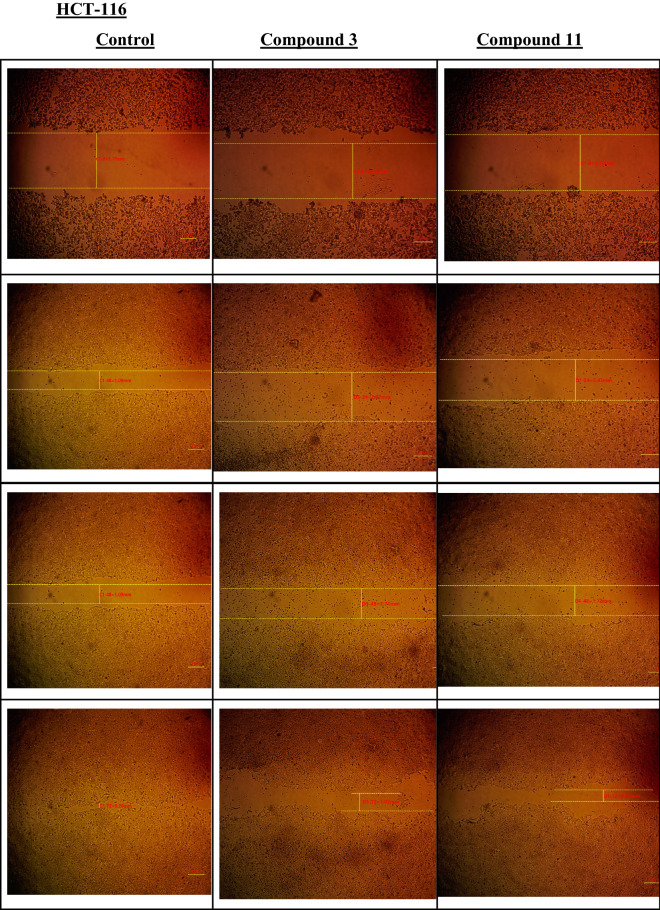
Wound closure percentage for both compounds **3** and **11** after three-time intervals, 24, 48, and 72 h on HCT-116 cells.

The two selected compounds **3** and **11** were also tested for their anti-migration effect on HCT-116 and SW-620 cancer cells. Using cell migration assay, against **5-FU** as positive control (Fig. 9).

From the above results, after 72 h of exposure to compound **3** it gave wound closure percentage (70%) compared to the control cells which gave (96%) on HCT-116 cells, and (27%) compared to control cells (31%) on metastatic cancer cells SW-620. On the other hand, compound **11** has given significant decrease on wound closure percentage on both cancer cells model (64% compared to control 96%) on HCT-116, and (18% compared to control cells 31%) on SW-620 colon cancer cells. This results supported that diphenyl pyrazole carboxamide derivative **11** has the ability of apoptosis induction and cell cycle arrest with higher potency than compound **3** and the standard treatment **5-FU** on metastatic cancer cells SW-620 (Fig. S9).

### Molecular docking

Molecular docking was conducted to predict the potential binding poses of the phenyl pyrazolo derivative **3** and the diphenyl pyrazolo derivative **11** with CA IX. PDB ID: 7pom was chosen as it is the most recent CA IX PDB crystal structure, with a co-crystallized ligand facilitating the identification of the binding site, and with a good resolution of 1.98 Å^48^. It is also the human variant for CA IX, on which the in vitro studies were carried out.

**Figure 10:**
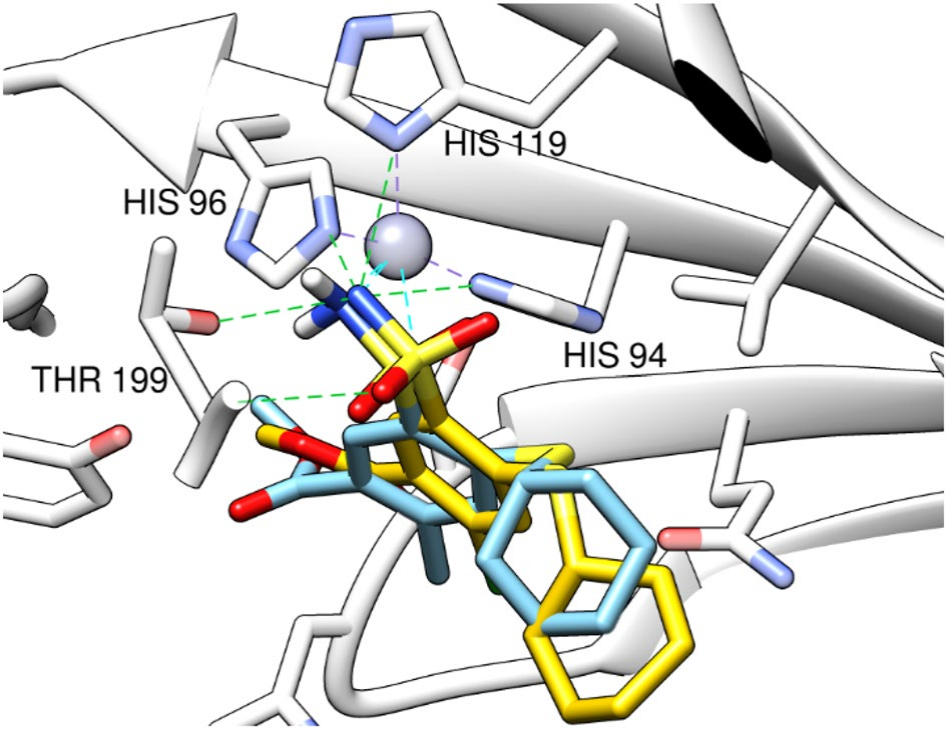
3D interaction diagrams for CA IX (PDB ID: 7pom) (grey) with sulfonamide co-crystallized ligand (yellow) and its docked pose (cyan). Grey dashed lines: coordination of histidine triad with zinc, cyan dashed lines: coordination of compounds with zinc, green dashed lines: conventional hydrogen bonds.

We used AutoDock vina^49^ for docking, AutoDock4Zn for protein force field description^50^ and AutoDock 4 for its scoring function^51^ to account for the zinc atom in CA zinc metalloprotein. First, we carried out a docking validation for the docking protocol by pose retrieval experiment via docking the co-crystallized ligand and measuring RMSD between the docked and co-crystallized poses. DockRMSD webserver^52^ was used for measuring RMSD which was equal to 1.4 Å. The software succeeded to predict the bioactive conformer pose with acceptable accuracy (Fig. 10). The docked pose of the co-crystallized ligand had a docking score of − 32.034 kcal/mol, reproducing the main binding interactions formed by the co-crystallized ligand. These interactions included five hydrogen bonds, three of them with the histidine triad of His94, His96 and His119 through the sulfonamide NH_2_ group, and two with Thr199 side chain OH and backbone NH groups. In addition, it retained the coordination with Zn^2+^ ion through the sulfonamide NH_2_ group (Fig. 10). 2D interactions of the co-crystallized ligand are depicted in Fig. S10 A.

**Figure 11:**
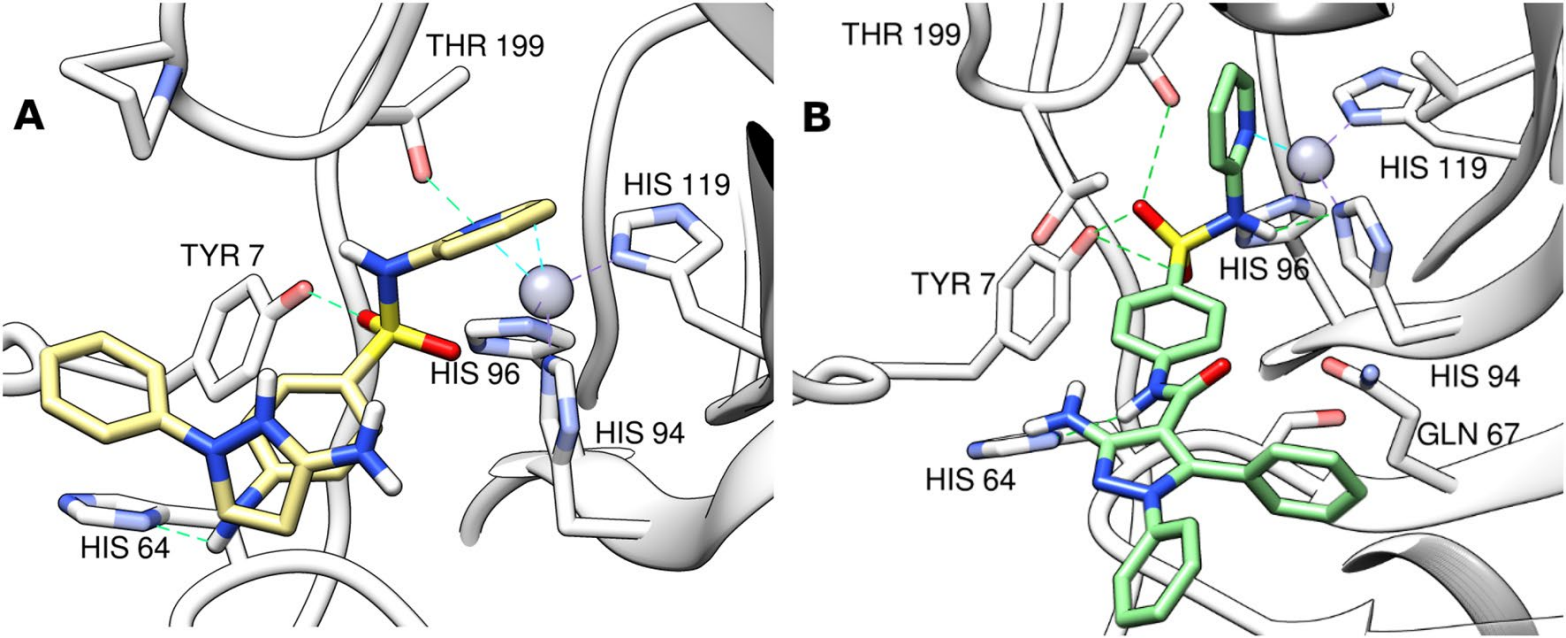
3D interaction diagrams for CA IX (PDB ID: 7pom) (grey) with compound **3** (khaki) (**A**) and compound **11** (green) (**B**). Grey dashed lines: coordination of histidine triad with zinc, cyan dashed lines: coordination of compounds with zinc, green dashed lines: conventional hydrogen bonds.

Compounds **3** and **11** showed comparable docking scores with that of the co-crystallized ligand, with docking scores of − 31.66 and − 31.56 kcal/mol, respectively, indicating their favorable binding to the protein. Both compounds maintained the Zn^2+^ metal coordination through the pyridine nitrogen atom (Fig. 10). Compound **3** formed a hydrogen bond through the SO_2_ group with Tyr7 residue, a hydrogen bond with His64 through the NH group of the aniline moiety, and a third hydrogen bond with Thr199 through the pyridine nitrogen atom (Fig. 11A). Compound **11** forms hydrogen bonds with Tyr7, Thr199 and Thr200 through SO_2_ group and another bond with Leu198 through the pyridine ring (Fig. 11B). Pi–sulfur interactions with His96 residue were formed through the sulfur atom in both compounds. The central phenyl ring in compound **3** forms a T-shaped pi–pi stack and pi–sigma interactions with His94 and Thr200 residues, in compound **11**, it forms a T-shaped pi–pi stack interaction with His94 (Fig. S10B,C). Also, the two phenyl rings attached to the pyrazole ring form pi–sulfur interaction with Arg60 in compound **11** (Fig. S10C). The stability of both compounds docked poses and their good docking scores can be attributed to these binding interactions.

### In vitro carbonic anhydrase IX inhibitory assay

In order to confirm the design strategy of our target compounds, and based on the docking results that supported the efficacy of dual tail approach in designing novel CA IX inhibitors, compounds **3** and **11** were evaluated for In vitro inhibitory evaluation against CA IX. The results were in correlation with the obtained docking scores, Compound **11** (0.128 µM) was more potent than compound **3** (0.336 µM) and of equal activity to Dorzolamide (0.122 µM) which is a known CA inhibitor, (Table 3).

**Table 3:** In-vitro CA IX inhibitory activity for compounds **3** and **11**.

Compound	CA IX (IC_50_ uM)*
**3**	0.336 ± 0.016
**11**	0.128 ± 0.006
**Dorzolamide**	0.122 ± 0.002

*Results are average of triplicate experiments ± SEM.

### Irradiation and purity

The most potent diphenyl pyrazolo derivative **11** was exposed to γ-radiation in an attempt to study its chemical stability. Radio-stability is important in means of sterilization of compounds and expecting their shelf life when introduced as drugs in pharmaceutical industry. Radiation sterilization showed success over conventional (chemical) methods with the scale of its applications rising. Compound **11** was irradiated at 25 kGy as a single dose in its solid state^53^. The physicochemical properties were compared, within days after irradiation. It was observed that there is no difference in the physico-chemical properties of compound **11** before and after irradiation. There was no change in solubility, odour, colour and form.

TLC and HPLC chromatographic techniques, were used to evaluate the purity of the irradiated compound **11**^54^. No change in Rf values, moreover no additional spots were observed in TLC. Close purity percentage of compound **11** was perceived in HPLC before and after the irradiation process (Fig. S11). In conclusion, compound **11** could be considered radio-stable, as it didn’t experience critical changes in its physico-chemical properties upon irradiation. Its stability could be encouraging to conduct more investigational *In-vivo* studies for compound **11** in future work using irradiated rat models.

## Conclusion

The present work describes the application of dual tail strategy for designing novel CA inhibitors. Novel 15 pyrazole-sulfonamide derivatives were synthesized, initial in vitro screening against three colon cancer cell lines identified compounds **3** and **11** as promising chemotherapeutic drugs as both induced cell death either by apoptosis, necrosis, or autophagy. The diphenyl pyrazolo **11** induced cell death by apoptosis and autophagy on HCT-116, and HT-29 cells but elicits cell death by necrosis, non-programmed cell death on SW-620 and by autophagy. The phenyl pyrazole **3** induced autophagy in the HCT-116, and SW-620, apoptosis in HCT-116 cells, necrosis in HT-29, and SW-620 cells. Also, both compounds have effects on cell cycle as compound **11** induced cell cycle arrest in G0/G1 phase in HCT-116, and SW-620 cells but induced S-Phase in HT-29 cells. Whereas, compound **3** decreased cell population in G0/G1 phase on HCT-116, and HT-29 with no effect on the metastatic cells SW-620. Moreover, compound **11** showed similar inhibitory activity as dorzolamide on CA IX and was found to be more active then compound **3** suggesting the importance of the presence of two unsubstituted aromatic rings for optimum hydrophobic interactions in the active site. These results introduce pyrazole-sulfonamide hybrids as promising scaffold to be further optimized to obtain more potent CA IX inhibitors of better therapeutic index for cancer treatment.

## Materials and methods

### Chemistry

#### General

Melting points were recorded uncorrected in open capillary tubes using Stuart melting point apparatus (Stuart Scientific, Redhill, UK). Infrared (IR) spectra of all newly synthesized compounds were reported using FTIR Shimadzu spectrometer (Shimadzu, Tokyo, Japan). Bruker 400 NMR spectrometer (Bruker Bioscience, MA, USA) was used at 400 and 100 MHz for recording ^1^H-NMR and ^13^C-NMR spectra, respectively. TMS was used as an internal standard, while deuterated DMSO was used as a solvent. HP Model MS-5988 (Hewlett Packard, Palo, Alto, California, USA) was used to determine mass spectra. To obtain microanalyses values, a Carlo Erba 1108 Elemental Analyzer (Heraeus, Hanau, Germany), was used. For checking of reactions’ Completion, Pre-coated SiO_2_ gel (200 mesh, HF254) aluminum plates (Merk, Darmstadt, Germany) were used as TLC. Where a developing solvent system of chloroform/methanol (6.5:3.5) was used. Spots were visualized under UV light. Spectral and elemental analysis were in consistent with the postulated structures. For purity inspection, HPLC was used (Agilent 1260 Infinity apparatus) on ZORBAX Eclipse Plus C18 column (4.6 × 100 mm, 3.5 µm), with a mobile phase acetonitrile:water mixture (60:40) at a flow rate of 1.0 mL/min and the detection wavelength was set at 250 nm.

##### General procedure for the preparation of 4-((3-Amino-1-substituted-1*H*-pyrazol-5-yl)amino)-*N*-(pyridin-2-yl) benzenesulfonamide (2–8)

A mixture of 2-Cyano-*N*-(4-(*N*-(pyridin-2-yl)sulfamoyl)phenyl)acetamide **1**^40^ (1.26 g, 0.004 mol) and the appropriate hydrazine derivatives (0.004 mol) in absolute ethanol (30 mL) was refluxed for 8–10 h then concentrated under vacuum. The formed precipitate was filtered off, washed with water and recrystallized from methanol to give compounds **2–8**, respectively.

##### 4-((3-Amino-1*H*-pyrazol-5-yl)amino)-*N*-(pyridin-2-yl)benzenesulfonamide (2)

Yield, 78%, mp 200–202 °C. IR (cm^−1^): 3320, 3230, 3200 (NH, NH_2_), 3046 (CH arom.), 1285, 1110 (SO_2_). ^1^H-NMR (DMSO-d_6_, ppm): 4.34 (s, 1H, CH-pyrazole), 4.62 (s, 2H, NH_2_, exchangeable with D_2_O), 6.67 (t, 1H, *J* = 7.5 Hz, CH-pyridine), 6.80 (d, 1H, *J* = 7.5 Hz, CH-pyridine), 7.31 (s, 1H, NH, exchangeable with D_2_O), 7.33 (d, 2H, *J* = 7.5 Hz, Ar–H), 7.44 (t, 1H, *J* = 7.5 Hz, CH-pyridine), 7.65 (s, 1H, NH, exchangeable with D_2_O), 7.96 (d, 2H, *J* = 7.5 Hz, Ar–H), 8.05 (d, 1H, *J* = 7.5 Hz, CH-pyridine), 11.14 (s, 1H, NH-pyrazole, exchangeable with D_2_O). ^13^C-NMR (DMSO-d_6_, ppm): 96.05 (CH-pyrazole), 115.70, 117.75 (2CH-pyridine), 120.72, 131.26 (4CH-phenyl), 136.97 (C-phenyl), 140.06 (CH-pyridine), 140.27 (C-pyrazole), 145.90 (C-phenyl), 148.83 (CH-pyridine), 155.06 (C-NH_2_), 155.64 (C-pyridine). MS m/z: 330 (M^+^). Analysis calculated for C_14_H_14_N_6_O_2_S: C, 50.90; H, 4.27; N, 25.44, found: C, 50.70; H, 4.31; N, 25.55.

##### 4-((3-Amino-1-phenyl-1*H*-pyrazol-5-yl)amino)-*N*-(pyridin-2-yl) benzenesulfonamide (3)

Yield, 75%, mp 230–232 °C. IR (cm^−1^): 3322, 3231, 3202 (NH, NH_2_), 3048 (CH arom.), 1315, 1105 (SO_2_). ^1^H-NMR (DMSO-d_6_, ppm): 4.70 (s, 2H, NH_2_, exchangeable with D_2_O), 4.91 (s, 1H, CH-pyrazole), 6.76 (t, 1H, *J* = 7.5 Hz, CH-pyridine), 6.84–6.88 (m, 2H, Ar–H), 7.10 (d, 2H, *J* = 7.5 Hz, CH-pyridine, Ar–H), 7.21(d, 2H, *J* = 7.5 Hz, Ar–H), 7.52 (t, 1H, *J* = 7.5 Hz, CH-pyridine), 7.59 (s, 1H, NH, exchangeable with D_2_O), 7.83 (d, 4H, *J* = 7.5 Hz, Ar–H), 8.12 (d, 1H, *J* = 7.5 Hz, CH-pyridine), 10.01 (s, 1H, NH, exchangeable with D_2_O). ^13^C-NMR (DMSO-d_6_, ppm): 82.97 (CH-pyrazole), 115.76, 117.85 (2CH-pyridine), 120.51, 125.24, 128.30, 129.28, 131.50 (9CH-phenyl), 136.99, 138.59 (2C-phenyl), 140.06 (CH-pyrazole), 140.27 (C-pyrazole), 147.70 (C-phenyl), 148.93 (CH-pyrazole), 153.51 (C-NH_2_), 155.74 (C-pyridine). MS m/z: 406 (M^+^). Analysis calculated for C_20_H_18_N_6_O_2_S: C, 59.10; H, 4.46; N, 20.68, found: C, 59.30; H, 4.34; N, 20.76.

##### 4-((3-Amino-1-(4-fluorophenyl)-1*H*-pyrazol-5-yl)amino)-*N*-(pyridin-2-yl)benzenesulfonamide (4)

Yield, 69%, mp 190–192 °C. IR (cm^−1^): 3319, 3232, 3202 (NH, NH_2_), 3047 (CH arom.), 1259, 1125 (SO_2_). ^1^H-NMR (DMSO-d_6_, ppm): 4.71 (s, 2H, NH_2_, exchangeable with D_2_O), 4.97 (s, 1H, CH-pyrazole), 6.73 (t, 1H, *J* = 7.5 Hz, CH-pyridine), 6.79 (d, 1H, *J* = 7.5 Hz, CH-pyridine), 7.02 (t, 2H, *J* = 7.5 Hz, Ar–H), 7.23 (d, 2H, *J* = 7.5 Hz, Ar–H), 7.50 (t, 1H, *J* = 7.5 Hz, CH-pyridine), 7.84–7.93 (m, 5H, Ar–H, NH, exchangeable with D_2_O), 8.09 (d, 1H, *J* = 7.5 Hz, CH-pyridine), 10.02 (s, 1H, NH, exchangeable with D_2_O). ^13^C-NMR (DMSO-d_6_, ppm): 82.87 (CH-pyrazole), 115.60, 117.07 (2CH-pyridine), 117.75, 120.61, 128.12, 131.60 (8CH-phenyl), 134.86, 136.89 (2C-phenyl), 140.26 (CH-pyridine), 140.47 (C-pyrazole), 147.24 (C-phenyl), 148.95 (CH-pyridine), 153.71 (C-NH_2_), 155.44 (C-pyridine), 163.84 (C-F). MS m/z: 424 (M^+^). Analysis calculated for C_20_H_17_FN_6_O_2_S: C, 56.59; H, 4.04; N, 19.80, found: C, 56.49; H, 4.24; N, 19.70.

##### 4-((3-Amino-1-(4-chlorophenyl)-1*H*-pyrazol-5-yl)amino)-*N*-(pyridin-2-yl)benzenesulfonamide (5)

Yield, 89%, mp 241–243 °C. IR (cm^−1^): 3327, 3235, 3250 (NH, NH_2_), 3045 (CH arom.), 1262, 1130 (SO_2_), 835 (C–Cl). ^1^H-NMR (DMSO-d_6_, ppm): 4.71 (s, 2H, NH_2_, exchangeable with D_2_O), 4.99 (s,1H, CH-pyrazole), 6.74–6.81 (m, 2H, CH-pyridine), 7.23, 7.25 (2d, 4H, *J* = 7.5 Hz, Ar–H), 7.52 (t, 1H, *J* = 7.5 Hz, CH-pyridine), 7.82, 7.91(2d, 4H, *J* = 7.5 Hz, Ar–H), 7.97(s,1H, NH, exchangeable with D_2_O), 8.10 (d, 1H, *J* = 7.5 Hz, CH-pyridine), 9.92 (s,1H, NH, exchangeable with D_2_O). ^13^C-NMR (DMSO-d_6_, ppm): 83.77 (CH-pyrazole), 115.80, 117.32 (2CH-pyridine), 117.35, 120.54, 127.75, 129.29 (8CH-phenyl), 135.02 (C–Cl), 134.16, 136.41 (2C-phenyl), 140.06 (CH-pyridine), 140.27 (C-pyrazole), 147.76 (C-phenyl), 148.75 (CH-pyridine), 153.91 (C-NH_2_), 155.54 (C-pyridine). MS m/z: 440(M^+^), 442 (M + 2). Analysis calculated for C_20_H_17_ClN_6_O_2_S: C, 54.48; H, 3.89; N, 19.06, found: C, 54.37; H, 3.79; N, 19.26.

##### 4-((3-Amino-1-(p-tolyl)-1*H*-pyrazol-5-yl)amino)-*N*-(pyridin-2-yl) benzenesulfonamide (6)

Yield, 82%, mp 215–217 °C. IR (cm^−1^): 3324, 3232, 3202 (NH, NH_2_), 3046 (CH arom.), 2949, 2870 (CH aliph.), 1255, 1115 (SO_2_). ^1^H-NMR (DMSO-d_6_, ppm): 2.25 (s, 3H, CH_3_), 4.72 (s, 2H, NH_2_, exchangeable with D_2_O), 4.98 (s, 1H, CH-pyrazole), 6.73 (t, 1H, *J* = 7.5 Hz, CH-pyridine), 6.82 (d, 1H, *J* = 7.5 Hz, CH-pyridine), 7.01, 7.23 (2d, 4H, *J* = 7.5 Hz, Ar–H), 7.50 (t, 1H, *J* = 7.5 Hz, CH-pyridine), 7.80, 7.91 (2d, 4H, *J* = 7.5 Hz, Ar–H), 7.96 (s, 1H, NH, exchangeable with D_2_O), 8.09 (d, 1H, *J* = 7.5 Hz, CH-pyridine), 9.87 (s,1H, NH, exchangeable with D_2_O). ^13^C-NMR (DMSO-d_6_, ppm): 21.13 (CH_3_), 82.27 (CH-pyrazole), 115.30, 117.27 (2CH-pyridine), 117.45, 120.81, 123.89, 130.89 (8CH-phenyl), 134.26, 136.01, 136.97 (3C-phenyl), 138.79 (C-CH_3_), 140.26 (CH-pyridine), 140.34 (C-pyrazole), 148.83 (CH-pyridine), 153.51 (C-NH_2_), 155.76 (C-pyridine). MS m/z: 420 (M^+^). Analysis calculated for C_21_H_20_N_6_O_2_S: C, 59.98; H, 4.79; N, 19.99, found: C, 59.88; H, 4.68; N, 19.79.

##### 4-((3-Amino-1-(4-nitrophenyl)-1*H*-pyrazol-5-yl)amino)-*N*-(pyridin-2-yl)benzenesulfonamide (7)

Yield, 74%, mp 237–239 °C. IR (cm^−1^): 3330, 3230, 3201 (NH, NH_2_), 3048 (CH arom.), 1228, 1108 (SO_2_). ^1^H-NMR (DMSO-d_6_, ppm): 4.71 (s, 2H, NH_2_, exchangeable with D_2_O), 4.85 (s,1H, CH-pyrazole), 6.53 (d, 1H, *J* = 7.5 Hz, CH-pyridine), 6.71 (t, 1H, *J* = 7.5 Hz, CH-pyridine), 7.28 (d, 2H, *J* = 7.5 Hz, Ar–H), 7.43 (t, 1H, *J* = 7.5 Hz, CH-pyridine), 7.97 (d, 3H, *J* = 7.5 Hz, Ar–H, NH, exchangeable with D_2_O), 8.06 (d, 2H, *J* = 7.5 Hz, Ar–H), 8.10 (d, 1H, *J* = 7.5 Hz, CH-pyridine), 8.21(d, 2H, *J* = 7.5 Hz, Ar–H), 11.22 (s, 1H, NH, exchangeable with D_2_O). ^13^C-NMR (DMSO-d_6_, ppm): 82.97 (CH-pyrazole), 115.70, 117.07 (2CH-pyridine), 117.75, 120.51, 121.16, 124.86 (8CH-phenyl), 134.87, 136.51 (2C-phenyl), 140.56 (CH-pyridine), 140.27 (C-pyrazole), 144.01 (C-phenyl), 145.10 (C-NO_2_), 148.94 (CH-pyridine), 153.71 (C-NH_2_), 155.64 (C-pyridine). MS m/z: 451(M^+^). Analysis calculated for C_20_H_17_N_7_O_4_S: C, 53.21; H, 3.80; N, 21.72, found: C, 53.3 1; H, 3.70; N, 21.67.

##### 4-((3-Amino-1-(4-methoxyphenyl)-1*H*-pyrazol-5-yl)amino)-*N*-(pyridin-2-yl)benzenesulfonamide (8)

Yield, 80%, mp 222–224 °C. IR (cm^−1^): 3322, 3233, 3205 (NH, NH_2_), 3045 (CH arom.), 2949, 2870 (CH aliph.), 1220, 1111 (SO_2_). ^1^H-NMR (DMSO-d_6_, ppm): 3.43 (s, 3H, OCH_3_), 4.73 (s, 2H, NH_2_, exchangeable with D_2_O), 5.53 (s,1H, CH-pyrazole), 6.66 (t, 1H, *J* = 7.5 Hz, CH-pyridine), 6.75 (d, 1H, *J* = 7.5 Hz, CH-pyridine), 7.18 (d, 4H, *J* = 7.5 Hz, Ar–H), 7.41 (t, 1H, *J* = 7.5 Hz, CH-pyridine), 7.57, 7.88 (2 s,2H, 2NH, exchangeable with D_2_O), 7.92 (d, 4H, *J* = 7.5 Hz, Ar–H), 8.04 (d, 1H, *J* = 7.5 Hz, CH-pyridine). ^13^C-NMR (DMSO-d_6_, ppm): 56.04 (OCH_3_), 82.67 (CH-pyrazole), 115.20, 117.47 (2CH-pyridine), 115.46, 117.75, 120.81, 127.78 (8CH-phenyl), 132.15, 134.16, 136.71 (3C-phenyl), 140.56 (CH-pyridine), 140.87 (C-pyrazole), 148.93 (CH-pyridine), 153.58 (C-NH_2_), 155.14 (C-pyridine), 159.15 (C-OCH_3_). MS m/z: 436 (M^+^). Analysis calculated for C_21_H_20_N_6_O_3_S: C, 57.79; H, 4.62; N, 19.25, found: C, 57.75; H, 4.57; N, 19.35.

##### 2-Cyano-3-phenyl-*N*-(4-(*N*-(pyridin-2-yl)sulfamoyl)phenyl acrylamide (9)

A mixture of 2-Cyano-*N*-(4-(*N*-(pyridin-2-yl)sulfamoyl)phenyl)acetamide **1** (1.26 g, 0.004 mol) and benzaldehyde (0.42 gm, 0.004 mol) was refluxed in absolute ethanol (30 mL) with 5 drops of piperidine for 8–10 h then cooled and poured onto ice water. The precipitated solid was filtered off, washed with water and recrystallized from dioxane to afford compound **9**.Yield, 85%, mp 245–247 °C. IR (cm^−1^): 3203 (NH), 3048 (CH arom.), 2959, 2880 (CH aliph.), 2200 (C≡N), 1720 (C=O), 1232, 1105 (SO_2_). ^1^H-NMR (DMSO-d_6_, ppm): 6.70 (t, 1H, *J* = 7.5 Hz, CH-pyridine), 6.82 (d, 1H, *J* = 7.5 Hz, CH-pyridine), 7.15–7.30 (m, 5H, Ar–H), 7.47 (t, 1H, *J* = 7.5 Hz, CH-pyridine), 7.69 (d, 2H, *J* = 7.5 Hz, Ar–H), 7.83 (s,1H, NH, exchangeable with D_2_O), 8.03 (d, 2H, *J* = 7.5 Hz, Ar–H), 8.07 (d, 1H, *J* = 7.5 Hz, CH-pyridine), 8.37 (s, 1H, CH), 10.24 (s, 1H, NH, exchangeable with D_2_O). ^13^C-NMR (DMSO-d_6_, ppm): 106.98 (C=CH), 115.70 (CH-pyridine), 116.44 (CN), 117.07 (CH-pyridine), 120.21, 128.78, 129.46, 129.50 (8CH-phenyl), 133.70, 133.76, 137.65 (3C-phenyl), 140.06 (CH-pyridine), 143.57 (C-phenyl), 148.83 (CH-pyridine), 150.79 (C=CH), 155.64 (C-pyridine), 165.96 (C=O). MS m/z: 404 (M^+^). Analysis calculated for C_21_H_16_N_4_O_3_S: C, 62.36; H, 3.99; N, 13.85, found: C, 62.26; H, 3.89; N, 13.76.

##### General procedure for the preparation of 3-amino-1-(substituted)-5-phenyl-*N*-(4-(*N*-(pyridin-2-yl)sulfamoyl)phenyl)-1*H*-pyrazole-4-carboxamide (10–16)

A mixture of 2-Cyano-3-phenyl-*N*-(4-(*N*-(pyridin-2-yl)sulfamoyl)phenyl acrylamide **(9)** (1.61 g, 0.004 mol) and the appropriate hydrazine derivatives (0.004 mol) in absolute ethanol (30 mL). The reaction mixture was refluxed for 8–10 h then concentrated under vacuum. The precipitated solid formed was filtered off, washed with water and recrystallized from ethanol/water to give **10–16**, respectively.

##### 3-Amino-5-phenyl-*N*-(4-(*N*-(pyridin-2-yl)sulfamoyl)phenyl)-1*H*-pyrazole-4-carboxamide (10)

Yield, 73%, mp 270–272 °C. IR (cm^−1^): 3332, 3223, 3210 (NH, NH_2_), 3047 (CH arom.), 1690 (C=O), 1222, 1117 (SO_2_). ^1^H-NMR (DMSO-d_6_, ppm): 5.23 (s, 2H, NH_2_, exchangeable with D_2_O), 6.67–6.75 (m, 2H, CH-pyridine), 7.07 (s, 1H, NH, exchangeable with D_2_O), 7.36–7.57 (m, 7H, Ar–H), 7.99 (d, 2H, *J* = 7.5 Hz, Ar–H), 8.13–8.14 (m, 3H, 2CH-pyridine, NH, exchangeable with D_2_O), 13.11 (s, 1H, NH-pyrazole, exchangeable with D_2_O). ^13^C-NMR (DMSO-d_6_, ppm): 105.26 (C-pyrazole), 115.80, 117.47 (2CH-pyridine), 120.51, 127.33, 127.91, 129.32, 129.46 (9CH-phenyl), 130.83, 137.85 (2C-phenyl), 140.06 (CH-pyridine),141.74 (C-pyrazole), 143.97 (C-phenyl), 145.77 (C-NH_2_), 148.53 (CH-pyridine),155.64 (C-pyridine), 170.75 (C=O). MS m/z: 434 (M^+^). Analysis calculated for C_21_H_18_N_6_O_3_S: C, 58.05; H, 4.18; N, 19.34, found: C, 58.25; H, 4.21; N, 19.24.

##### 3-Amino-1,5-diphenyl-*N*-(4-(*N*-(pyridin-2-yl)sulfamoyl)phenyl)-1*H*-pyrazole-4-carboxamide (11)

Yield, 88%, mp > 280 °C. IR (cm^−1^): 3329, 3235, 3209 (NH, NH_2_), 3048 (CH arom.), 1720 (C=O), 1230, 1108 (SO_2_). ^1^H-NMR (DMSO-d_6_, ppm): 4.84 (s, 2H, NH_2_, exchangeable with D_2_O), 6.60 (s, 1H, NH, exchangeable with D_2_O), 6.74–6.84 (m, 2H, CH-pyridine), 6.98–7.08 (m, 3H, Ar–H), 7.30–7.50 (m, 6H, Ar–H), 7.62, 7.68 (2d, 4H, *J* = 7.5 Hz, Ar–H), 8.00 (d, 2H, *J* = 7.5 Hz, 2CH-pyridine), 8.17 (d, 1H, *J* = 7.5 Hz, Ar–H), 10.28 (s, 1H, NH, exchangeable with D_2_O). ^13^C-NMR (DMSO-d_6_, ppm): 112.21 (C-pyrazole), 115.10, 117.13 (2CH-pyridine), 120.51, 122.89, 127.93, 128.42, 128.61, 129.11, 129.56, 129.73 (14CH-phenyl), 131.59, 137.95 (2C-phenyl), 140.16 (CH-pyridine), 140.30, 143.77 (2C-phenyl), 145.32 (C-pyrazole), 148.83 (CH-pyridine), 153.14 (C-NH_2_), 155.74 (C-pyridine), 173.62 (C=O). MS m/z: 510 (M^+^). Analysis calculated for C_27_H_22_N_6_O_3_S: C, 63.52; H, 4.34; N, 16.46, found: C, 63.42; H, 4.24; N, 16.37.

##### 3-Amino-1-(4-fluorophenyl)-5-phenyl-*N*-(4-(*N*-(pyridin-2-yl)sulfamoyl) phenyl)-1*H*-pyrazole-4-carboxamide (12)

Yield, 71%, mp 261–263 °C. IR (cm^−1^): 3320, 3230, 3200 (NH, NH_2_), 3045 (CH arom.), 1680 (C=O), 1315, 1105 (SO_2_). ^1^H-NMR (DMSO-d_6_, ppm): 5.04 (s, 2H, NH_2_, exchangeable with D_2_O), 6.76–6.93 (m, 2H, CH-pyridine, NH, exchangeable with D_2_O), 6.83 (d, 1H, *J* = 7.3 Hz, CH-pyridine), 6.91 (t, 2H, *J* = 7.8 Hz, Ar–H), 7.33–7.35 (m, 1H, Ar–H), 7.42 (t, 2H, *J* = 7.4 Hz, Ar–H), 7.46–7.49 (m, 3H, CH-pyridine, Ar–H), 7.55, 7.64 (2d, 4H, *J* = 7.5 Hz, Ar–H), 7.99 (d, 2H, *J* = 7.5 Hz, Ar–H), 8.09 (s,1H, NH, exchangeable with D_2_O), 8.18 (d, 1H, *J* = 7.5 Hz, CH-pyridine). ^13^C-NMR (DMSO-d_6_, ppm): 112.31 (C-pyrazole), 115.40 (CH-pyridine), 115.89 (2CH-phenyl), 117.57 (CH-pyridine), 120.41, 125.09, 128.62, 128.91, 129.66, 129.93 (11CH-phenyl), 131.47, 135.96, 137.98 (3C-phenyl), 140.65 (CH-pyridine), 143.77 (C-phenyl), 145.76 (C-pyrazole), 148.93 (CH-pyridine), 153.94 (C-NH_2_), 155.54 (C-pyridine), 162.78 (C-F), 173.92 (C=O). MS m/z: 528(M^+^). Analysis calculated for C_27_H_21_FN_6_O_3_S: C, 61.35; H, 4.00; N, 15.90, found: C, 61.25; H, 4.21; N, 15.70.

##### 3-Amino-1-(4-chlorophenyl)-5-phenyl-*N*-(4-(*N*-(pyridin-2-yl)sulfamoyl) phenyl)-1*H*-pyrazole-4-carboxamide (13)

Yield, 83%, mp 256–258 °C. IR (cm^−1^): 3320, 3230, 3200 (NH, NH_2_), 3046 (CH arom.), 1720 (C=O), 1285, 1110 (SO_2_), 835 (C–Cl). ^1^H-NMR (DMSO-d_6_, ppm): 5.04 (s, 2H, NH_2_, exchangeable with D_2_O), 6.73–6.84 (m, 3H, CH-pyridine, Ar–H, NH, exchangeable with D_2_O), 7.12 (d, 2H, *J* = 7.5 Hz, CH-pyridine, Ar–H), 7.40–7.49 (m, 6H, CH-pyridine, Ar–H), 7.54, 7.64 (2d, 4H, *J* = 7.5 Hz, Ar–H), 7.99 (d, 2H, *J* = 7.5 Hz, Ar–H), 8.09 (s, 1H, NH, exchangeable with D_2_O), 8.18 (d, 1H, *J* = 7.5 Hz, CH-pyridine). ^13^C-NMR (DMSO-d_6_, ppm): 112.41 (C-pyrazole), 115.89, 117.57 (2CH-pyridine), 120.34, 124.73, 128.52, 128.81, 128.94, 129.66, 129.97 (13CH-phenyl), 131.64 (C-phenyl), 133.65 (C–Cl), 137.75, 138.63 (2C-phenyl), 140.46 (CH-pyridine), 143.76 (C-phenyl), 145.53 (C-pyrazole), 148.93 (CH-pyridine), 153.94 (C-NH_2_), 155.98 (C-pyridine), 173.82 (C=O). MS m/z: 544(M^+^), 546 (M + 2). Analysis calculated for C_27_H_21_ClN_6_O_3_S: C, 59.50; H, 3.88; N, 15.42, found: C, 59.40; H, 3.76; N, 15.53.

##### 3-Amino-5-phenyl-*N*-(4-(*N*-(pyridin-2-yl)sulfamoyl)phenyl)-1-(p-tolyl)-1*H*-pyrazole-4-carboxamide (14)

Yield, 77%, mp 269–272 °C. IR (cm^−1^): 3322, 3231, 3202 (NH, NH_2_), 3048 (CH arom.), 2949, 2870 (CH aliph.), 1690 (C=O), 1315, 1105 (SO_2_). ^1^H-NMR (DMSO-d_6_, ppm): 2.30 (s, 3H, CH_3_), 5.03 (s, 2H, NH_2_, exchangeable with D_2_O), 6.74 (s,1H, NH, exchangeable with D_2_O), 6.76 (d, 1H, *J* = 7.5 Hz, CH-pyridine), 6.83 (d, 1H, *J* = 7.5 Hz, CH-pyridine), 6.91 (d, 2H, *J* = 7.5 Hz, Ar–H), 7.33(t, 1H, *J* = 7.5 Hz, Ar–H), 7.40–7.43 (m, 4H, Ar–H), 7.49 (t, 1H, *J* = 7.5 Hz, CH-pyridine), 7.55, 7.64, 7.99 (3d, 6H, *J* = 7.5 Hz, Ar–H), 8.08 (s,1H, NH, exchangeable with D_2_O), 8.19 (d, 1H, *J* = 7.5 Hz, CH-pyridine). ^13^C-NMR (DMSO-d_6_, ppm): 21.13 (CH_3_), 112.41 (C-pyrazole), 115.80, 117.15 (2CH-pyridine), 120.31, 125.66, 128.27, 128.62, 128.71, 129.66, 129.93 (13CH-phenyl), 131.49, 137.85 (2C-phenyl), 138.26 (C-CH_3_), 139.27 (C-phenyl), 140.18 (CH-pyridine), 143.77 (C-phenyl), 145.42 (C-pyrazole), 148.76 (CH-pyridine), 153.23 (C-NH_2_), 155.53 (C-pyridine), 173.87 (C=O). MS m/z: 524 (M^+^). Analysis calculated for C_28_H_24_N_6_O_3_S: C, 64.11; H, 4.61; N, 16.02, found: C, 64.22; H, 4.51; N, 16.13.

##### 3-Amino-1-(4-nitrophenyl)-5-phenyl-*N*-(4-(*N*-(pyridin-2-yl)sulfamoyl) phenyl)-1*H*-pyrazole-4-carboxamide (15)

Yield, 72%, mp 261–263 °C. IR (cm^−1^): 3319, 3232, 3202 (NH, NH_2_), 3047 (CH arom.), 1720 (C=O), 1259, 1125 (SO_2_). ^1^H-NMR (DMSO-d_6_, ppm): 4.84 (s, 2H, NH_2_, exchangeable with D_2_O), 6.26 (s,1H, NH, exchangeable with D_2_O), 6.75 (t, 1H, *J* = 7.4 Hz, CH-pyridine), 6.84 (d, 1H, *J* = 7.5 Hz, CH-pyridine), 7.33 (t, 1H, *J* = 7.5 Hz, Ar–H),7.41 (t, 2H, *J* = 7.5 Hz, Ar–H), 7.49 (t, 1H, *J* = 7.5 Hz, CH-pyridine), 7.63 (t, 4H, *J* = 7.7 Hz, Ar–H), 7.76 (d, 2H, *J* = 7.5 Hz, Ar–H), 7.94 (d, 2H, *J* = 7.5 Hz, Ar–H), 8.04 (d, 2H, *J* = 7.5 Hz, Ar–H), 8.11 (d, 1H, *J* = 7.5 Hz, CH-pyridine), 10.20 (s,1H, NH, exchangeable with D_2_O). ^13^C-NMR (DMSO-d_6_, ppm): 112.41 (C-pyrazole), 115.90, 117.57 (2CH-pyridine), 120.41, 124.30, 124.32, 128.65, 128.71, 129.96, 129.99 (13CH-phenyl), 131.59, 137.95 (2C-phenyl), 140.36 (CH-pyridine), 143.65 (C-phenyl), 144.08 (C-phenyl), 145.12 (C-pyrazole), 146.72 (C-NO_2_), 148.63 (CH-pyridine), 153.34 (C-NH_2_), 155.54 (C-pyridine), 173.92 (C=O). MS m/z: 555 (M^+^). Analysis calculated for C_27_H_21_N_7_O_5_S: C, 58.37; H, 3.81; N, 17.65, found: C, 58.27; H, 3.78; N, 17.53.

##### 3-Amino-1-(4-methoxyphenyl)-5-phenyl-*N*-(4-(*N*-(pyridin-2-yl)sulfamoyl)phenyl)-1*H*-pyrazole-4-carboxamide (16)

Yield, 80%, mp 275–277 °C. IR (cm^−1^): 3327, 3235, 3250 (NH, NH_2_), 3045 (CH arom.), 2939, 2877 (CH aliph.), 1680 (C=O), 1262, 1130 (SO_2_). ^1^H-NMR (DMSO-d_6_, ppm): 3.76 (s, 3H, OCH_3_), 4.96 (s, 2H, NH_2_, exchangeable with D_2_O), 6.62–6.67 (m, 3H, CH-pyridine, Ar–H), 6.76–6.84 (m, 2H, Ar–H), 6.99 (s,1H, NH, exchangeable with D_2_O), 7.31–7.33 (m, 3H, Ar–H), 7.39–7.48 (m, 5H, Ar–H, NH, exchangeable with D_2_O), 7.55, 7.99, 8.17 7.76 (3d, 5H, *J* = 7.5 Hz, CH-pyridine, Ar–H). ^13^C-NMR (DMSO-d_6_,ppm): 56.04 (OCH_3_), 112.31 (C-pyrazole), 115.50 (CH-pyridine), 114.39 (2CH-phenyl), 117.27 (CH-pyridine), 120.31, 125.34, 128.52, 128.71, 129.66, 129.83 (11CH-phenyl), 131.69, 134.18, 137.95 (3C-phenyl), 140.16 (CH-pyridine), 143.77 (C-phenyl), 145.52 (C-pyrazole), 148.73 (CH-pyridine), 153.94 (C-NH_2_), 155.64 (C-pyridine), 158.70 (C-OCH_3_), 173.32 (C=O). MS m/z: 540(M^+^). Analysis calculated for C_28_H_24_N_6_O_4_S: C, 62.21; H, 4.47; N, 15.55, found: C, 62.32; H, 4.53; N, 15.65.

### Biological assays

#### Cell culture

Human colorectal adenocarcinoma cell lines (HCT-116, SW-620 and HT-29), and the normal oral epithelial cells OEC were obtained from Nawah Scientific (Cairo, Egypt). Cells were maintained in Dulbecco’s Modified Eagle Medium (DMEM) (Lonza GmbH, Köln, Germany). Media were supplemented with 1% penicillin/streptomycin (Lonza GmbH, Köln, Germany) and 10% heat-inactivated fetal bovine serum (FBS; Gibco, NY, USA). Cells were passaged in a humidified (BINDER, Tuttlingen, Germany) at 37 °C with a 5% (v/v) CO_2_ atmosphere.

#### Cytotoxicity assays

Cytotoxicity was tested by SRB assay as previously described. Cells were collected using 0.25% trypsin/EDTA (Lonza GmbH, Köln, Germany) and seeded in 96 well plates (Greiner bio-one, Germany) at 1000–2000 cells/well. Day after seeding, tested compounds were added over cells and kept for 72 h. Afterward, cells proteins were fixed with 10% trichloroacetic acid (TCA) (Merck, 8.22342.1000) for 1 h at 4 °C, then washed three times. After fixation, cells were exposed to 0.4% Sulforhodamine B (SRB) (Sigma-Aldrich, 230162-5G) for 10 min in dark then washed with 1% glacial acetic acid. After drying overnight, Tris (hydroxymethyl) aminomethane v.p-TRIS (50 mM, pH 7.4) (Chem-Lab) was used to dissolve SRB-stained cells and colour intensity was measured at 540 nm using BMG LABTECH®- FLUOstar Omega microplate reader (Allmendgrün, Ortenberg, Germany).

#### Apoptosis assessment

Annexin V-FITC apoptosis detection kit (ab14085; Abcam, Cambridge, MA) was used to detect apoptotic and necrotic cell populations. Cells were exposed to the predetermined IC_50_’s of the tested compounds, and compound-free media (control group) for 48 h. Cells were harvested and washed twice with PBS, then incubated with 0.5 mL of Annexin V-FITC/PI solution, in the dark, for 30 min at room temperature. After staining, the cells were injected via the ACEA Novocyte™ flowcytometer (ACEA Biosciences Inc., San Diego, CA, USA). For each sample, 12,000 events were acquired and positive FITC and/or PI cells were quantified by quadrant analysis and calculated using the ACEA NovoExpress™ software (ACEA Biosciences Inc., San Diego, CA, USA)^25^.

#### Autophagy assay

Autophaghy assay was done to further investigate the mechanism of cellular death by which colorectal cancer cells might have used in response to the treatment with the synthesized compounds **3** and **11**. Autophagic cell death was assessed quantitatively using acridine orange coupled with cytometric analysis. The cells were synchronously exposed to the predetermined IC_50_’s of the tested compounds and **5-FU** as the reference drug. After treatment, the cells were collected, rinsed twice with PBS and stained with acridine orange (10 µg/mL) while incubated at 37 °C for 30 min in the dark. After staining, the cells were then injected via ACEA Novocyte™ flowcytometer (ACEA Biosciences Inc., San Diego, CA, USA) and analyzed for Cyto-ID differential green/orange, fluorescent signals using the FL1 and FL2 signal detectors, respectively (λex/em 488/530 nm for FITC and λex/em 535/617 nm for PI). For each sample, 12,000 events were acquired, and mean green net fluorescent intensities (NFI) were quantified using the ACEA NovoExpress™ software (ACEA Biosciences Inc., San Diego, CA, USA)^25^.

#### Cell cycle assay

To assess the effect of compounds **3**, **11**, and **5-FU** on cell cycle distribution, HCT-116, HT-29 cells and SW-620 were subjected to the pre-determined IC_50_s’ of the tested compounds or compound-free media for 48 h. After treatment, the cells were collected by trypsinization and rinsed twice with ice-cold PBS, then re-suspended in 0.5 mL of PBS. 2 mL of 60% ice-cold ethanol were added gently while vortexing, and the cells were incubated at 4 °C for 1 h for fixation. Upon analysis, fixed cells were washed and re-suspended in 0.5 mL of PBS containing 50 µg/mL RNAase A and 10 µg/mL propidium iodide. After 20 min of incubation in the dark at 37 °C, the cells were analysed for DNA content using flow cytometry analysis FL2 (λex/em 535/617 nm) signal detector (ACEA Novocyte™ flowcytometer, ACEA Biosciences Inc., SanDiego, CA, USA). For each sample, 12,000 events were acquired. Cell cycle distribution was calculated using the ACEA NovoExpress™ software (ACEA Biosciences Inc., San Diego, CA, USA).

#### Assessment of tumorigenic selectivity

SRB assay was done on normal cells as previously described for the two selected compounds and the reference drug 5-FU.

#### Wound healing assay

Cells were plated at density 2 × 10^5^/well onto a coated 12-well plate for scratch wound assay and cultured overnight in 5% FBS-DMEM at 37 °C and 5% CO_2_. On the next day, horizontal scratches were introduced into the confluent monolayer; the plate was washed thoroughly with PBS, control wells were replenished with fresh medium while drug wells were treated with fresh media containing drug. Images were taken using an inverted microscope at the indicated time intervals. The plate was incubated at 37 °C and 5% CO_2_ in-between time points. The acquired images were displayed here and were analyzed by MII ImageView software version 3.7. The gap was measured at certain time intervals and was compared to the initial gap area at time t = 0. Wound closure (expressed as a percentage) was calculated from the following equation:

$$ {\text{Wound closure}}\;\% {:}\;\left( {{\text{wt}} = 0\,{\text{h}}{-}{\text{wt}} = \Delta {\text{h/wt}} = 0\,{\text{h}}} \right) \times 100 $$

### Statistical analysis

Statistical analysis of IC_50_ values was calculated from concentration–response curves by Sigma Plot software, version 12.0 (System Software, San Jose, CA, USA), using an E-max model equation^24^:

$$ \% \;{\text{Cell viability}} = \left( {100 - {\text{R}}} \right) \times \left( {1 - \frac{{\left[ {\text{D}} \right]^{{\text{m}}} }}{{{\text{K}}_{{\text{d}}}^{{\text{m}}} + \left[ {\text{D}} \right]^{{\text{m}}} }}} \right) + {\text{R}} $$

where (R) is the residual unaffected fraction (the resistance fraction), (D) is the compound concentration used, (K_d_) is the compound concentration that produces a 50% reduction of the maximum inhibition rate, and (m) is a Hill-type coefficient. IC_50_ was defined as the compound concentration required to reduce absorbance to 50% of that of the control (i.e., K_d_ = IC_50_ when R = 0 and Emax = 100 − R). All experiments were performed in triplicate wells for each condition.

Statistical data were analyzed by one-way ANOVA and NewmanKeuls was used as post-hoc test. Graph-Pad Prism (V5, Co., San Diego, USA) was used for the statistical analysis. The differences between groups were considered significant at *p < 0.05.

### Molecular docking

The protein structure of CA IX was downloaded from Protein Data Bank (RCSB PDB)^21^ with PDB ID: 7pom^22^. The protein structure was prepared by removing water molecules, deleting all chains except chain A, and removing co-crystallized ligand and other small molecules through AutoDock Tools 1.5.6^17^. Then, using prepare_receptor4.py script from AutoDock Tools 1.5.7 the prepared pdb was converted to pdbqt format by assigning charges. The zinc_pseudo.py script was used to generate the Zn repulsive component and the attractive component by a TZ pseudoatom^18^. The search space dimensions were determined by GetBox-PyMOL-Plugin with the box centered around the co-crystallized ligand with center at − 27, 12.7, − 27 and dimensions of 25 Å for each axis. Receptor grid file was generated by prepare_gpf4zn.py script for autogrid4 which then generated the grid maps. Ligands were drawn using MarvinSketch^23^, then converted to 3D and energy minimized using OpenBabel 2.4.1 via steepest descent minimization algorithm for 10,000 steps and a convergence criterion of 10^−6^ kcal/mol/Å implementing MMFF94s (Merck Molecular Force Field static variant) for stepwise energy calculations^24^. It was then prepared in pdbqt format using mk_prepare_ligand.py script. Finally, docking was carried out using AutoDock vina 1.2.3^25^. Figures generation was done using UCSF Chimera visualization software^26^.

### Carbonic anhydrase IX inhibitory assay

The inhibitory effect of compounds **3** and **11** against CA-IX was measured using Carbonic Anhydrase (CA) Inhibitor Screening Kit (Catalog # K473-100, BioVision™, Milpitas, CA, USA) through using the purchased Carbonic Anhydrase IX (CA9) (AA 1-459) protein (GST tag) (Catalog # ABIN1347791, antibodies-online GmbH, Aachen, Germany). The assay was performed following the instructions of the assay kit manufacturer. Briefly, compounds **3** and **11** and the positive control Dorzolamide HCl, were dissolved at a final concentration equals to 10X in DMSO and incubated with the assay buffer and the enzyme for 10 min at room temperature. The enzyme substrate (Five μL) were added, mixed well then absorbance was measured at 405 nm in a kinetic mode for 1 h at room temperature. Slopes were calculated from the linear range. All experiments were done as three independent experiments and results were reported as average ± standard deviation (SD).

### Irradiation

Dry pure compound **11**, was collected in polyethylene vials wrapped with an aluminium scabbard then subjected to one dose of 25 kGy γ-rays^52^. ^60^Co source (Indian-Gamma Cell (Ge 4000 A) was utilized for irradiation at a dose rate of 1.208 kGy/h.

## Supplementary Information

Supplementary Information.

## Data Availability

All data generated or analysed for this study are included in this published paper (and its Supplementary Information files).
