# Supplementary material for: Pyrazole-sulfonamide scaffold featuring dual-tail strategy as apoptosis inducers in colon cancer
Source: Sci Rep. 2023 Apr 8;13:5782. doi: 10.1038/s41598-023-32820-0 (PMC10082777; doi:10.1038/s41598-023-32820-0)
Supplement: Supplementary file 1 — Supplementary Information. [file 41598_2023_32820_MOESM1_ESM.docx]

**Supplementary data**


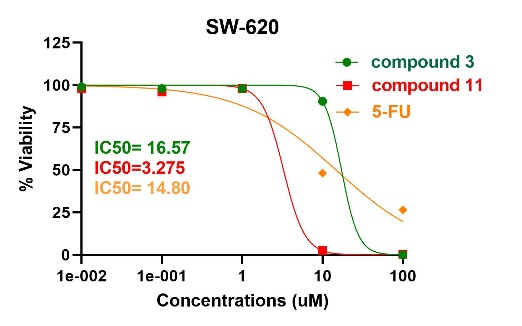

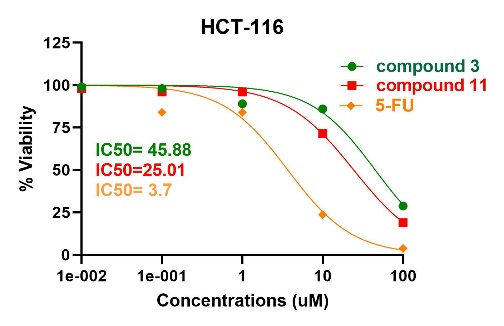

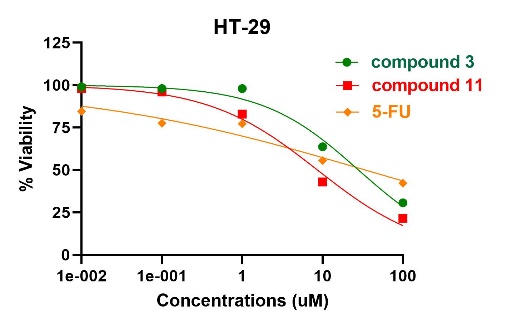


**Figure S1.** Dose response curves for compounds **3** and **11** and **5-FU** on the three cell lines; HT-29, HCT-116 and SW-620.

| 1. **Compound 3**  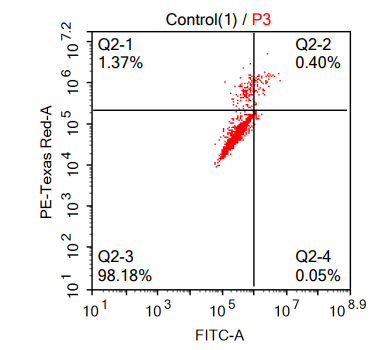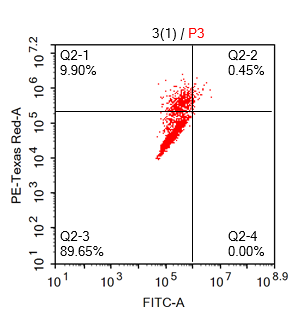 |
| --- |
| 1. **Compound 11**  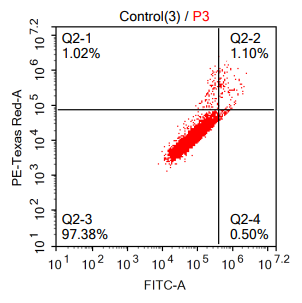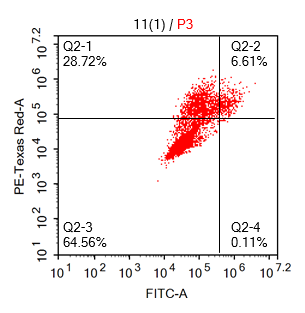 |
| 1. **5-FU**  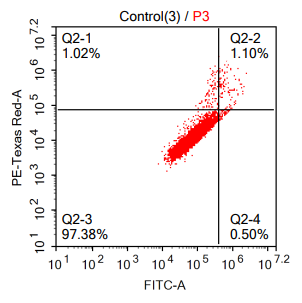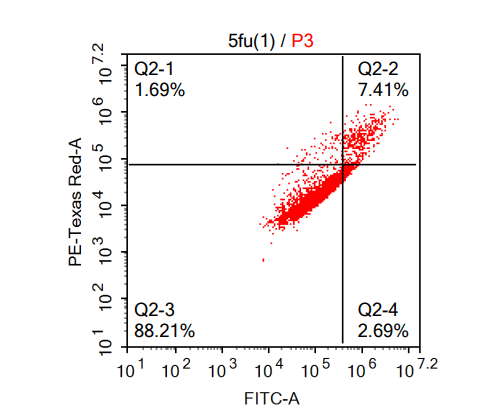 |

**Figure S2.** Apoptosis assessment for compounds **3**, **11** and **5-FU** on HT-29 cells; A, B and C, respectively.

1. **Compound 3**


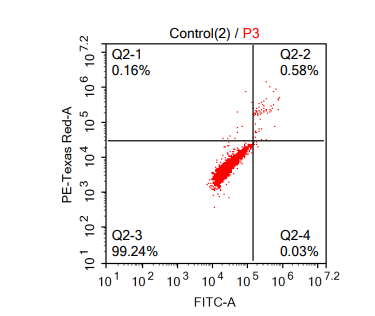

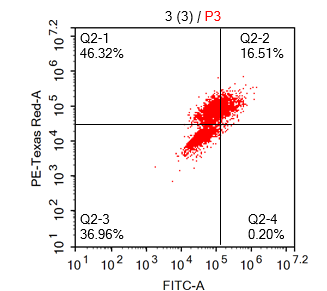

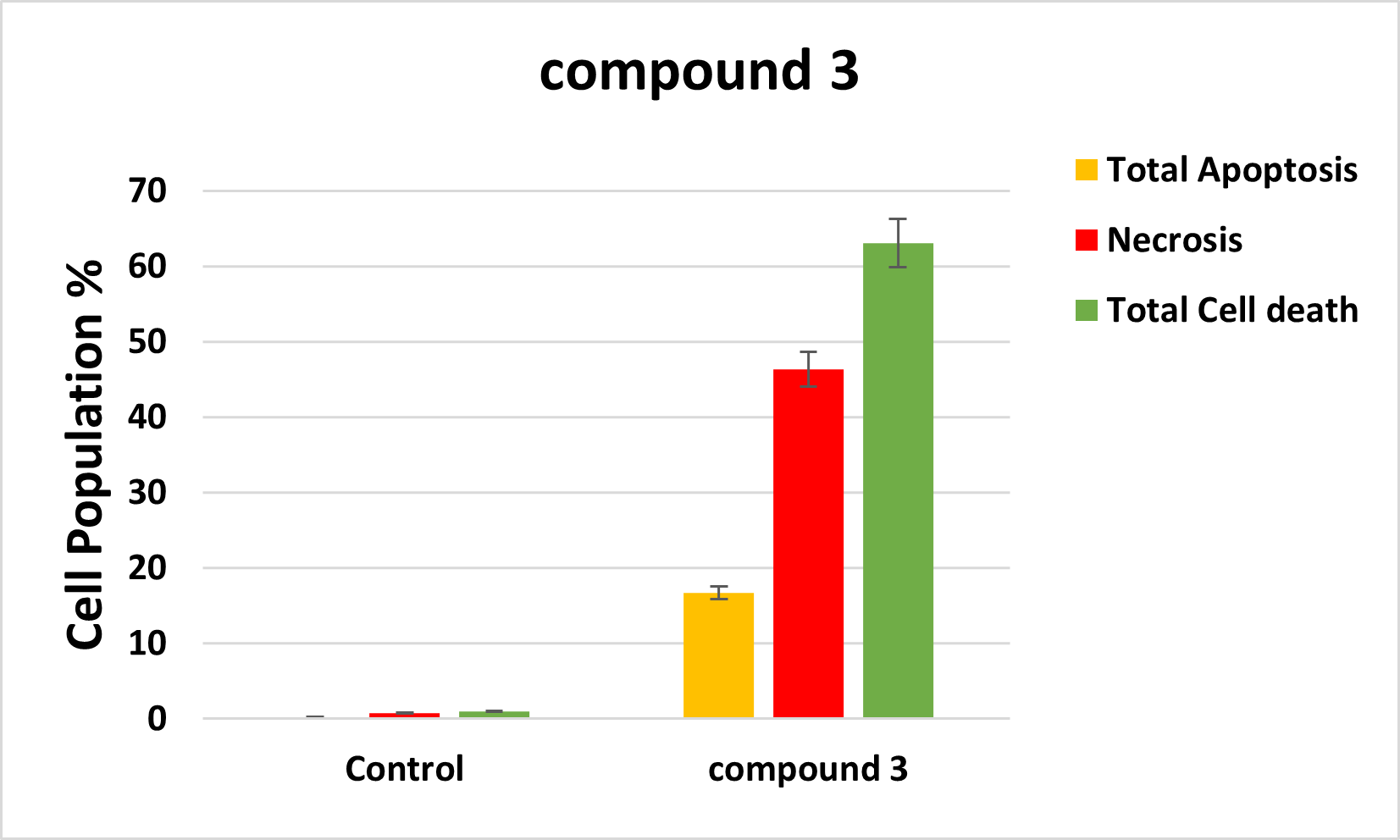


1. **Compound 11**


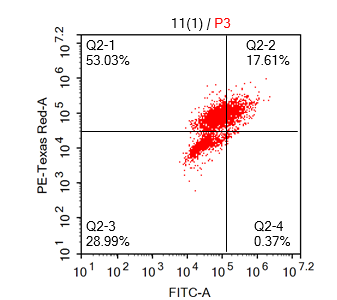

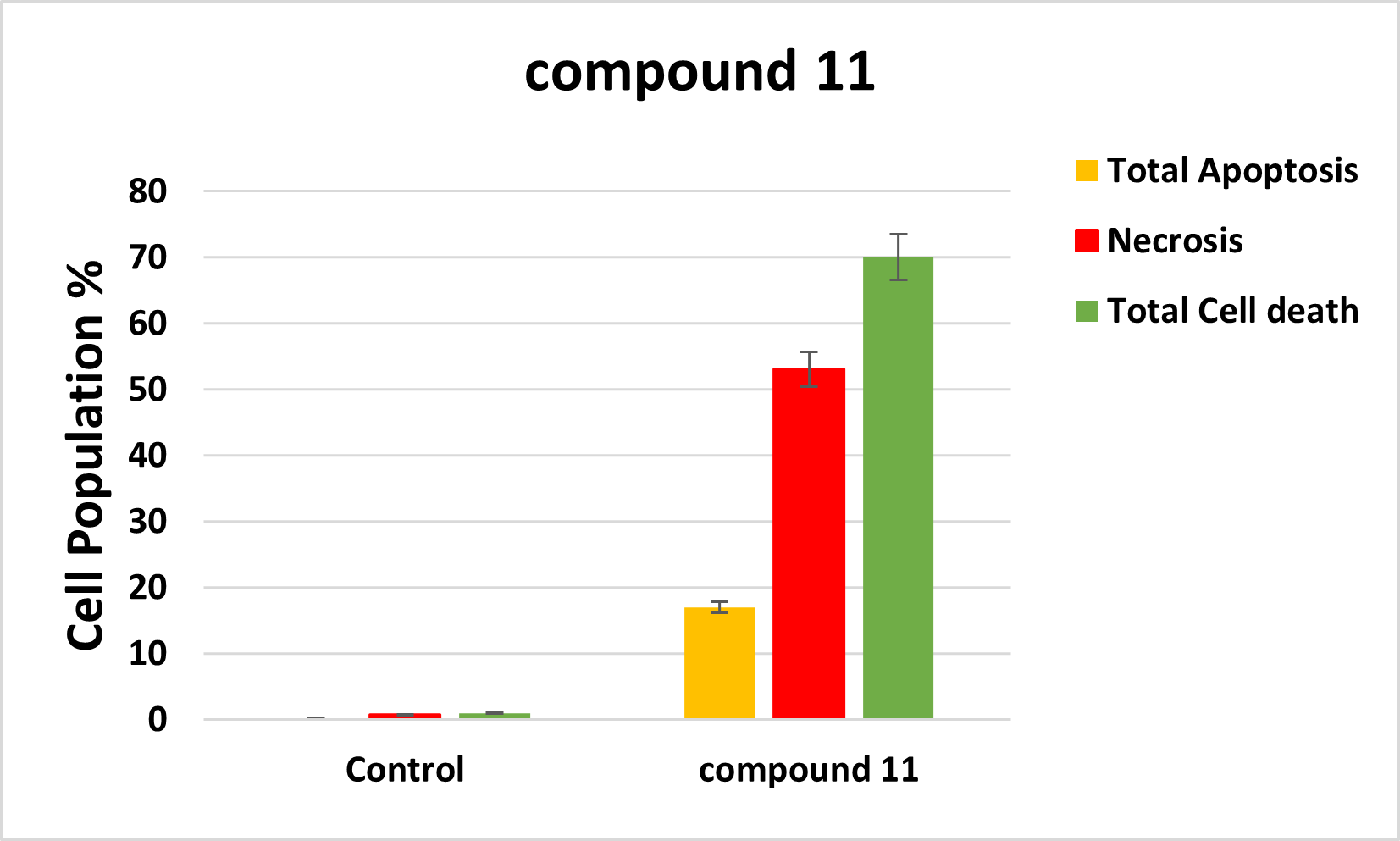

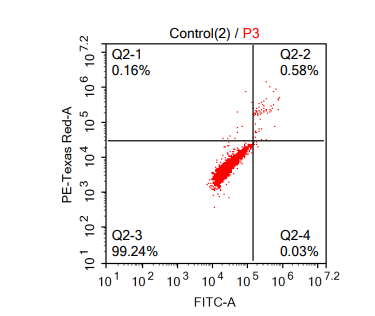


1. **5-FU**


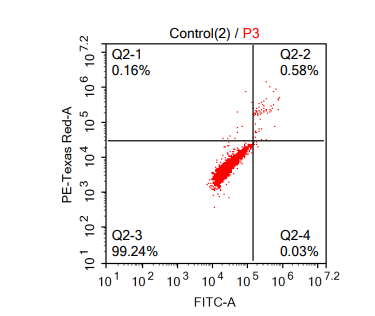

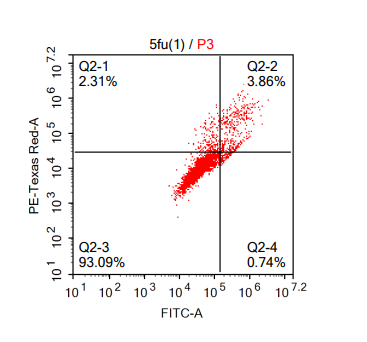

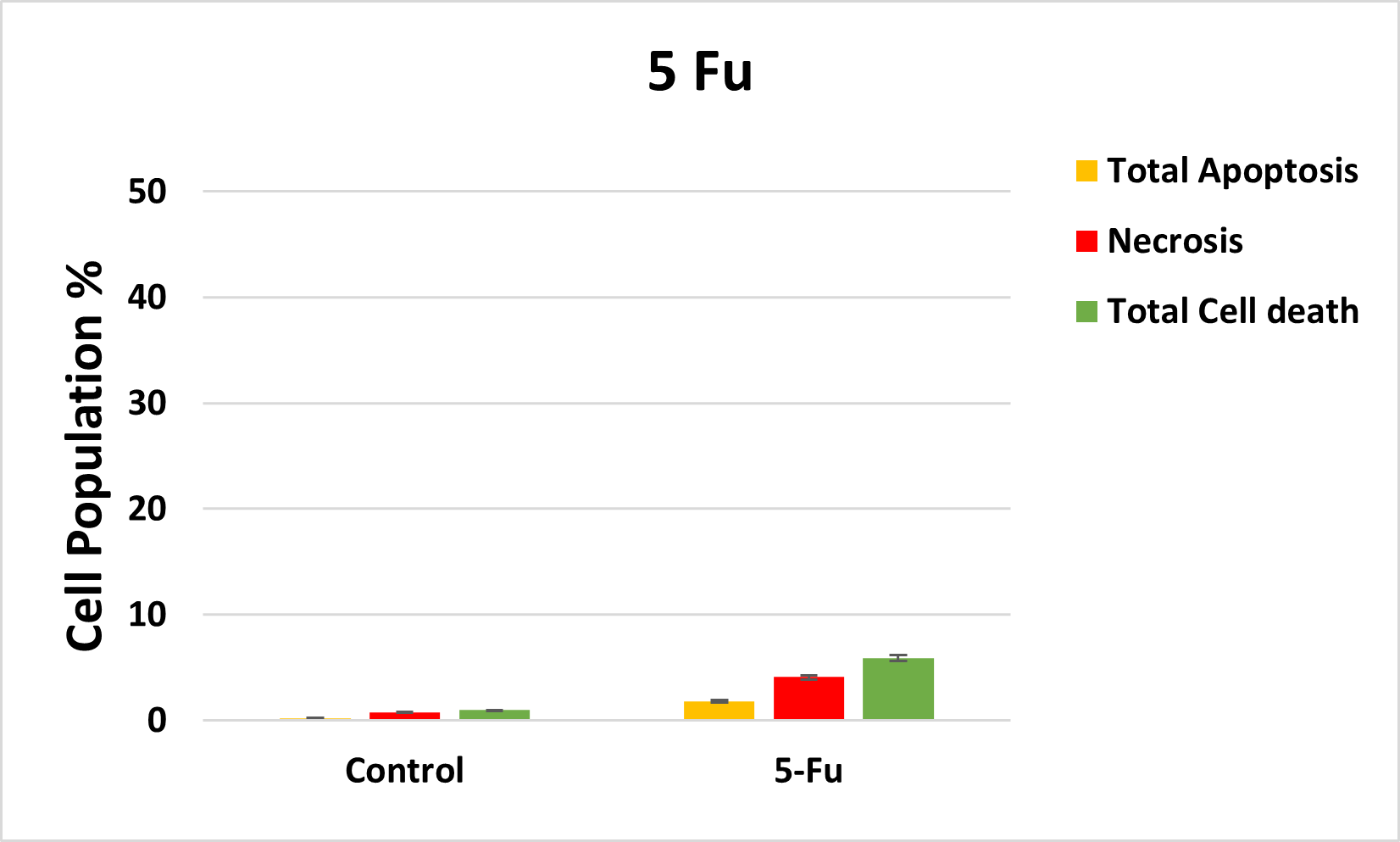


**Figure S3.** Apoptosis assessment for compounds **3**, **11** and **5-FU** on SW-620 cells; A, B and C, respectively.

1. **Compound 3**


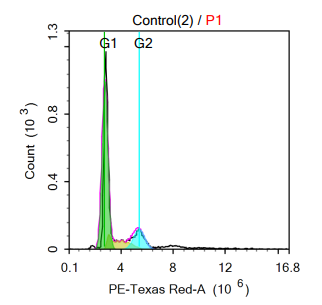

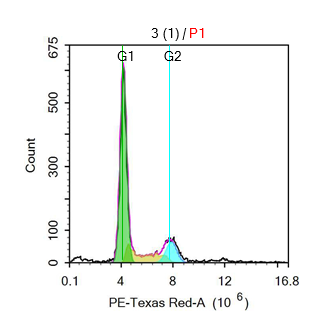

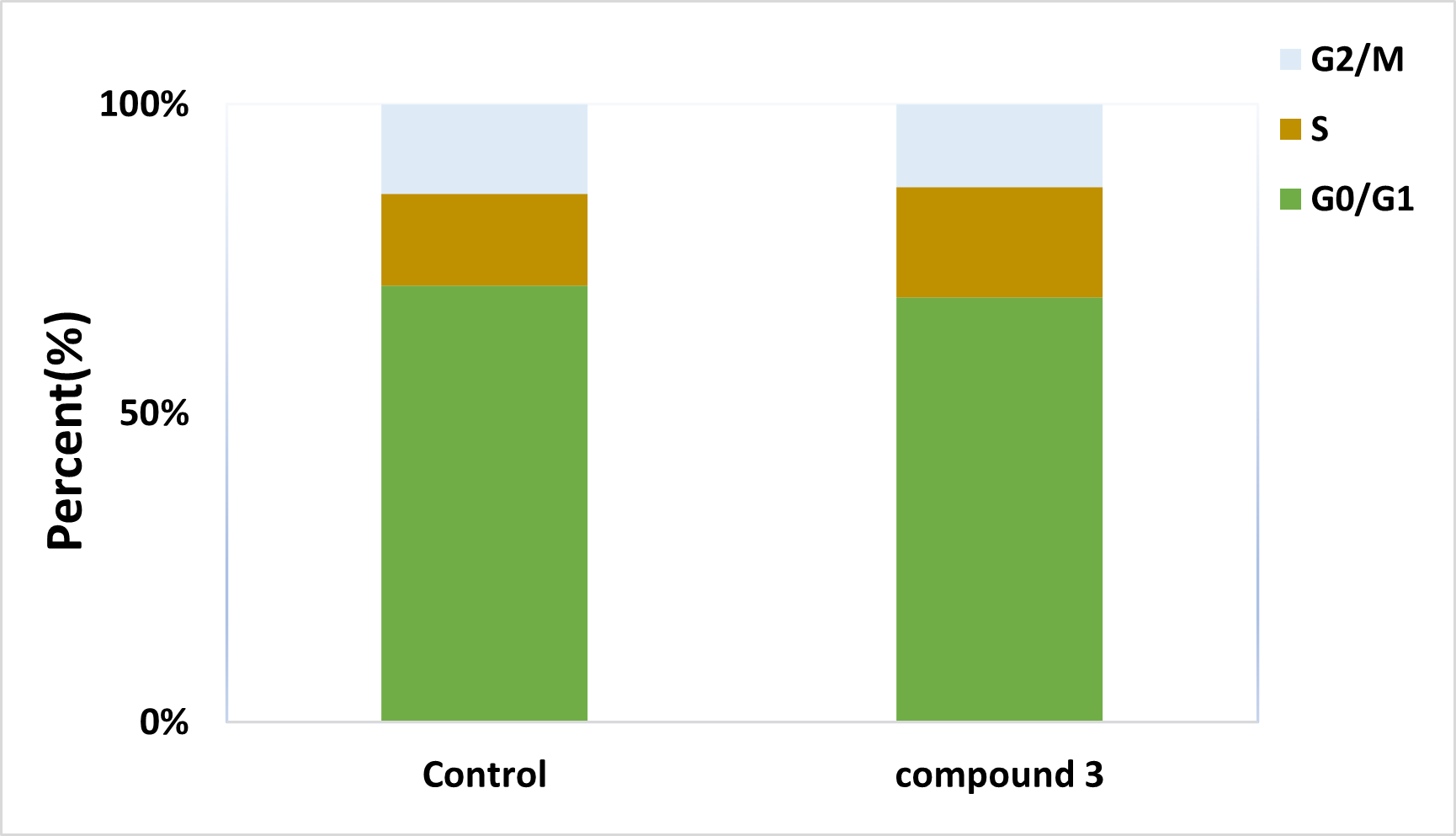


1. **Compound 11**


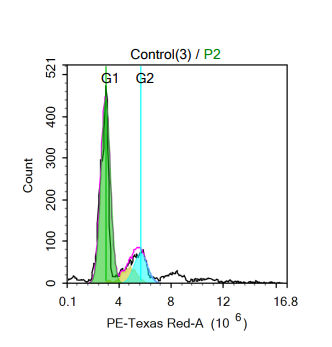

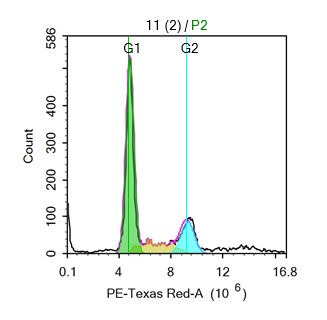

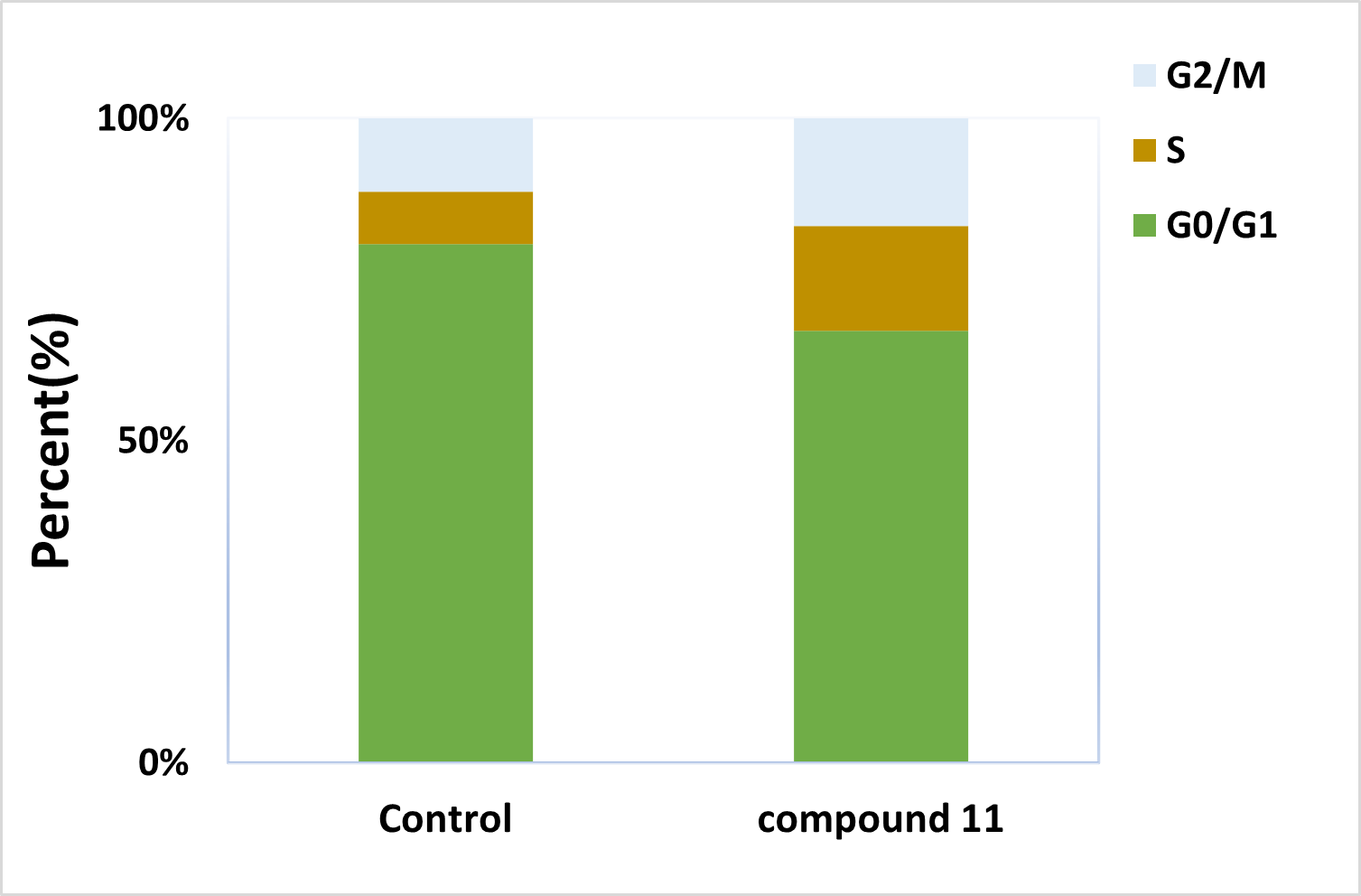


1. **5-FU**


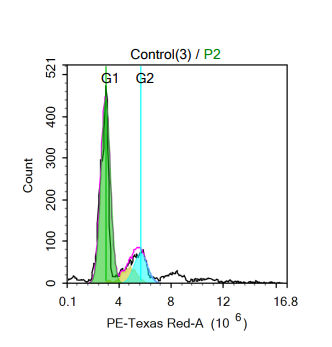

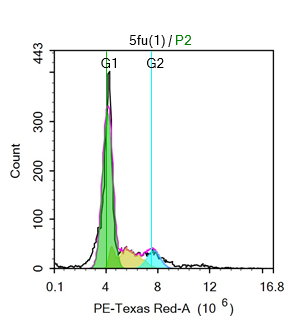

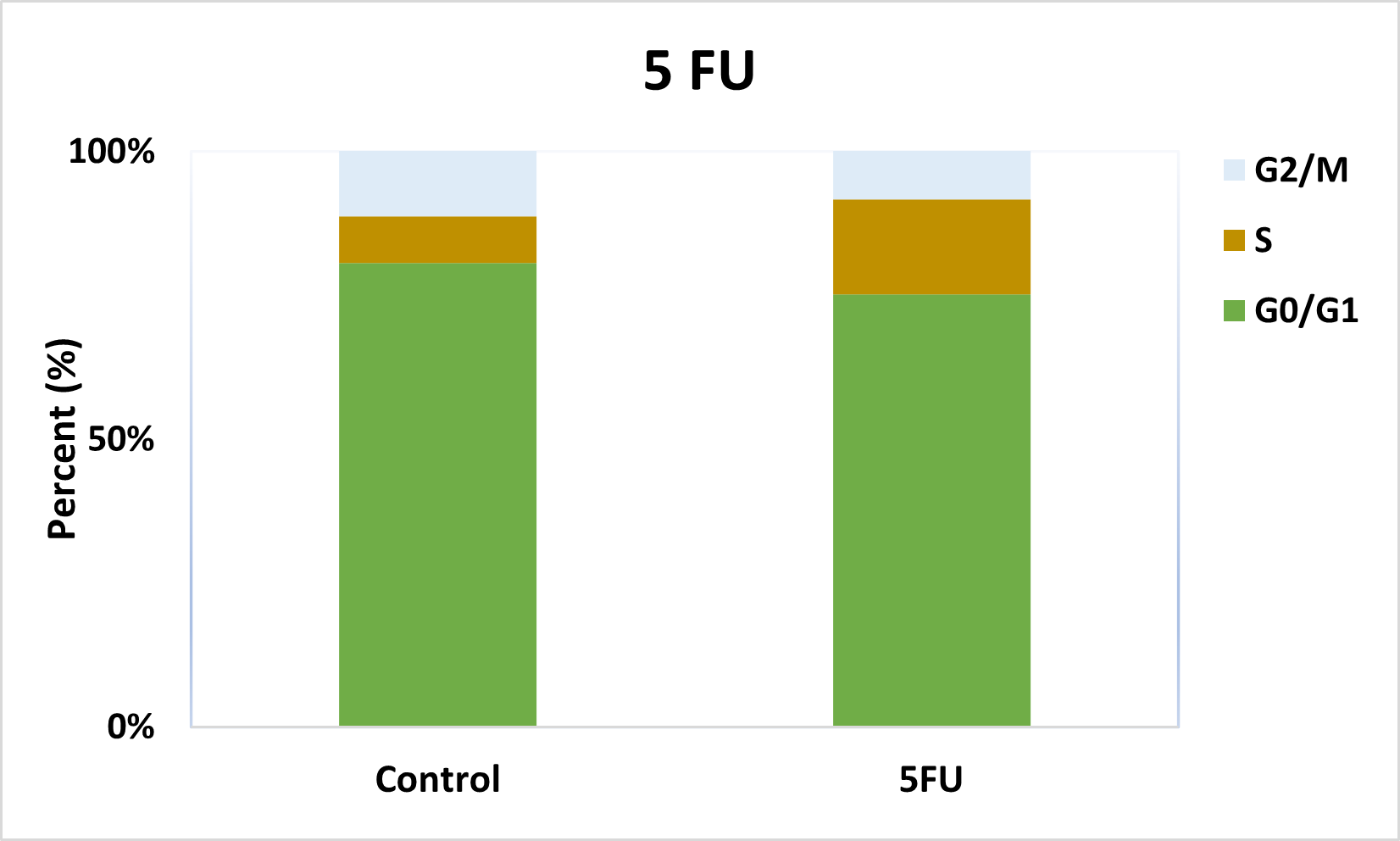


**Figure S4.** Cell cycle assessment for compounds **3**, **11** and **5-FU** on HT-29 cells; A, B and C, respectively.

1. **compound 3**


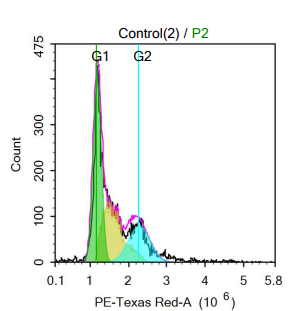

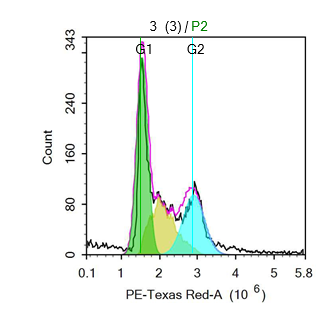

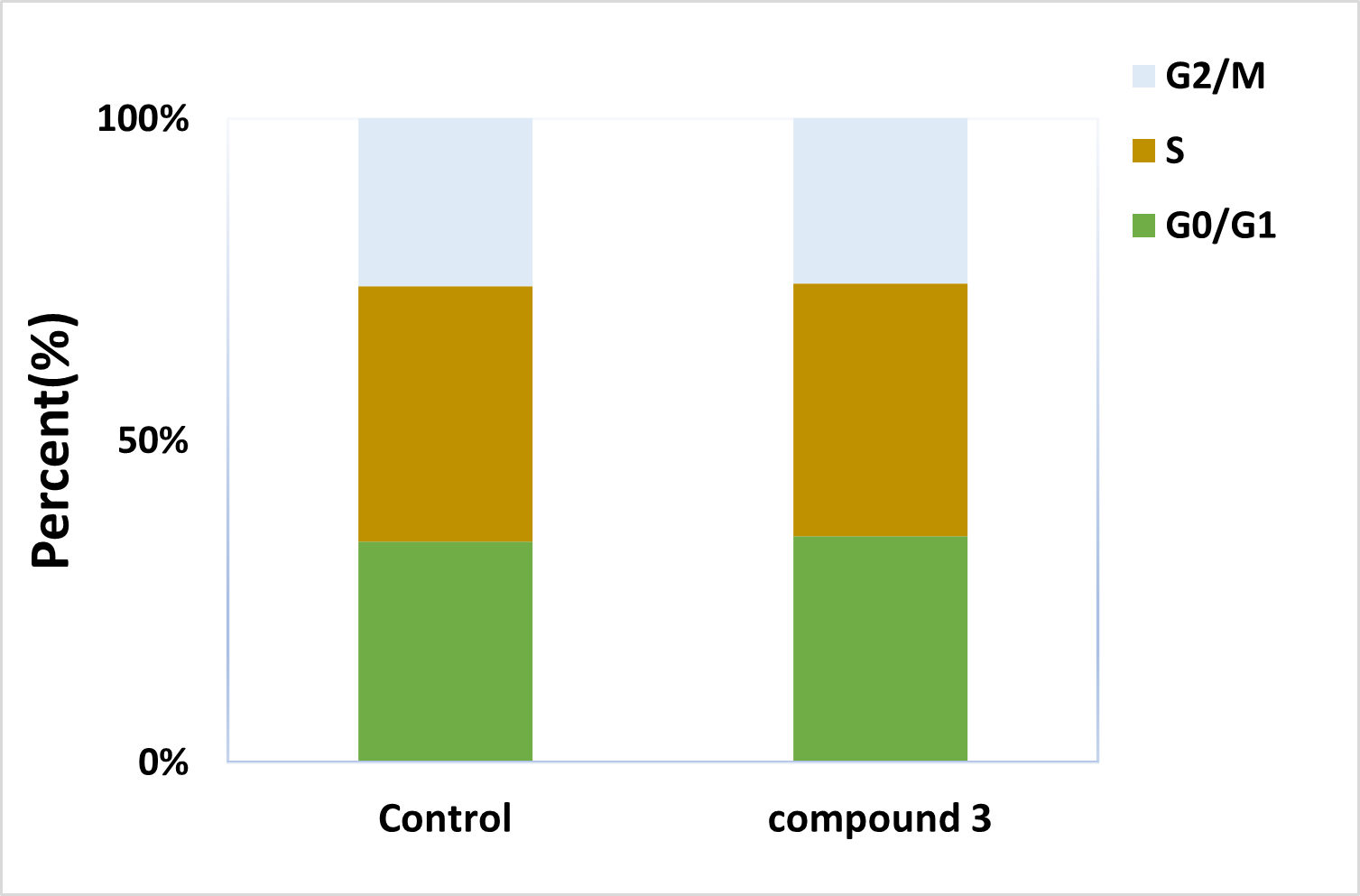


1. **Compound 11**


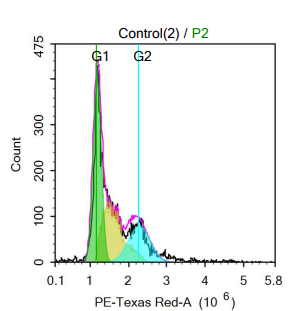

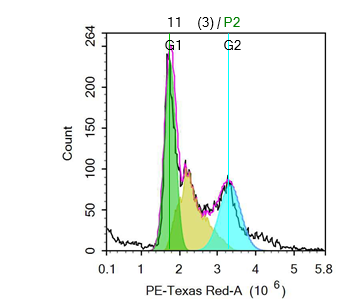

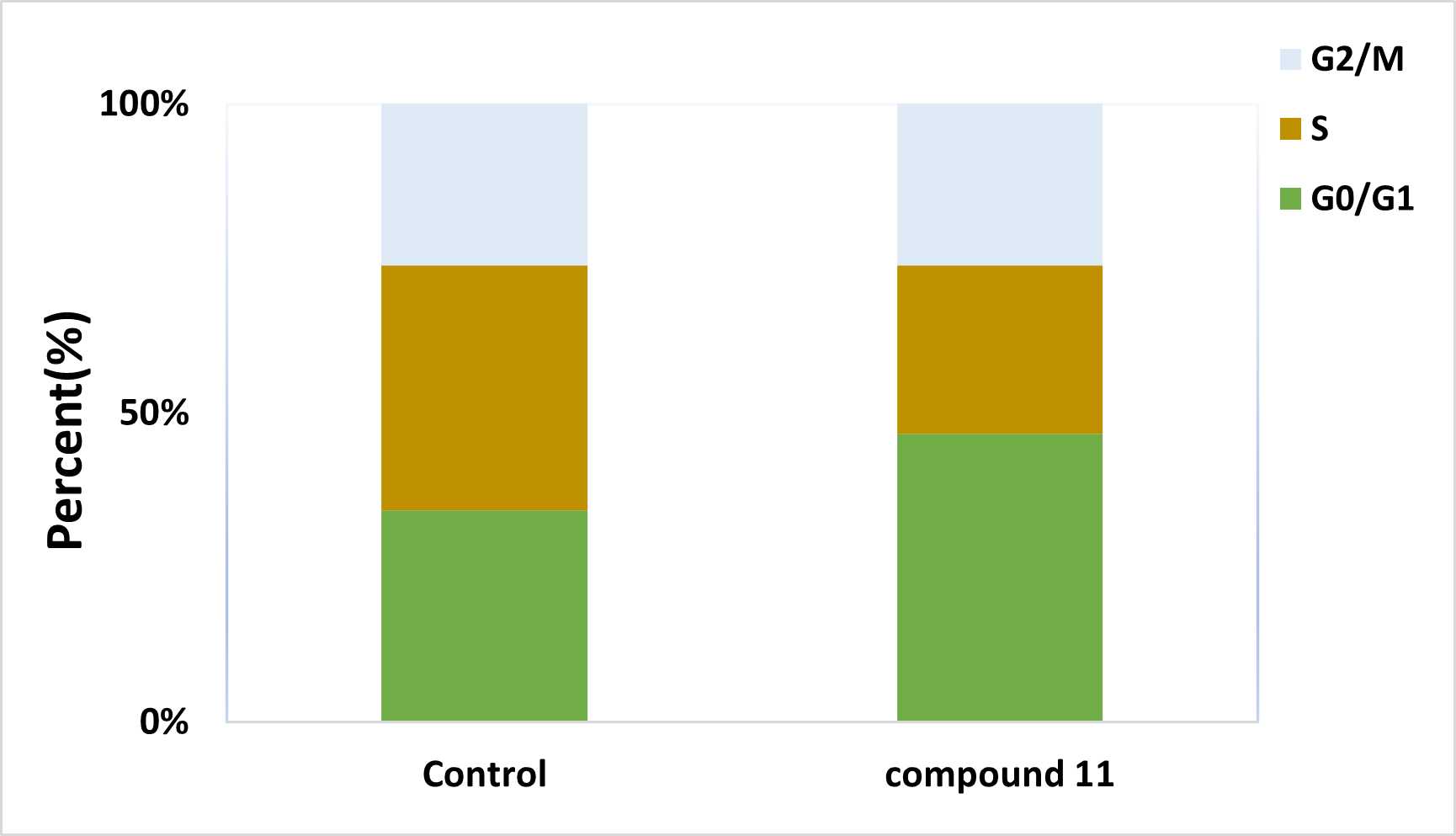


1. **5-FU**


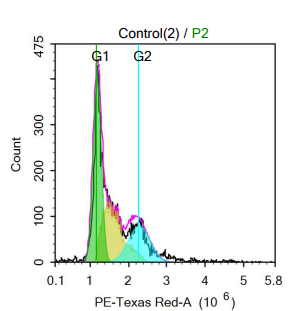

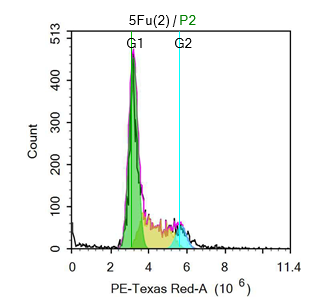

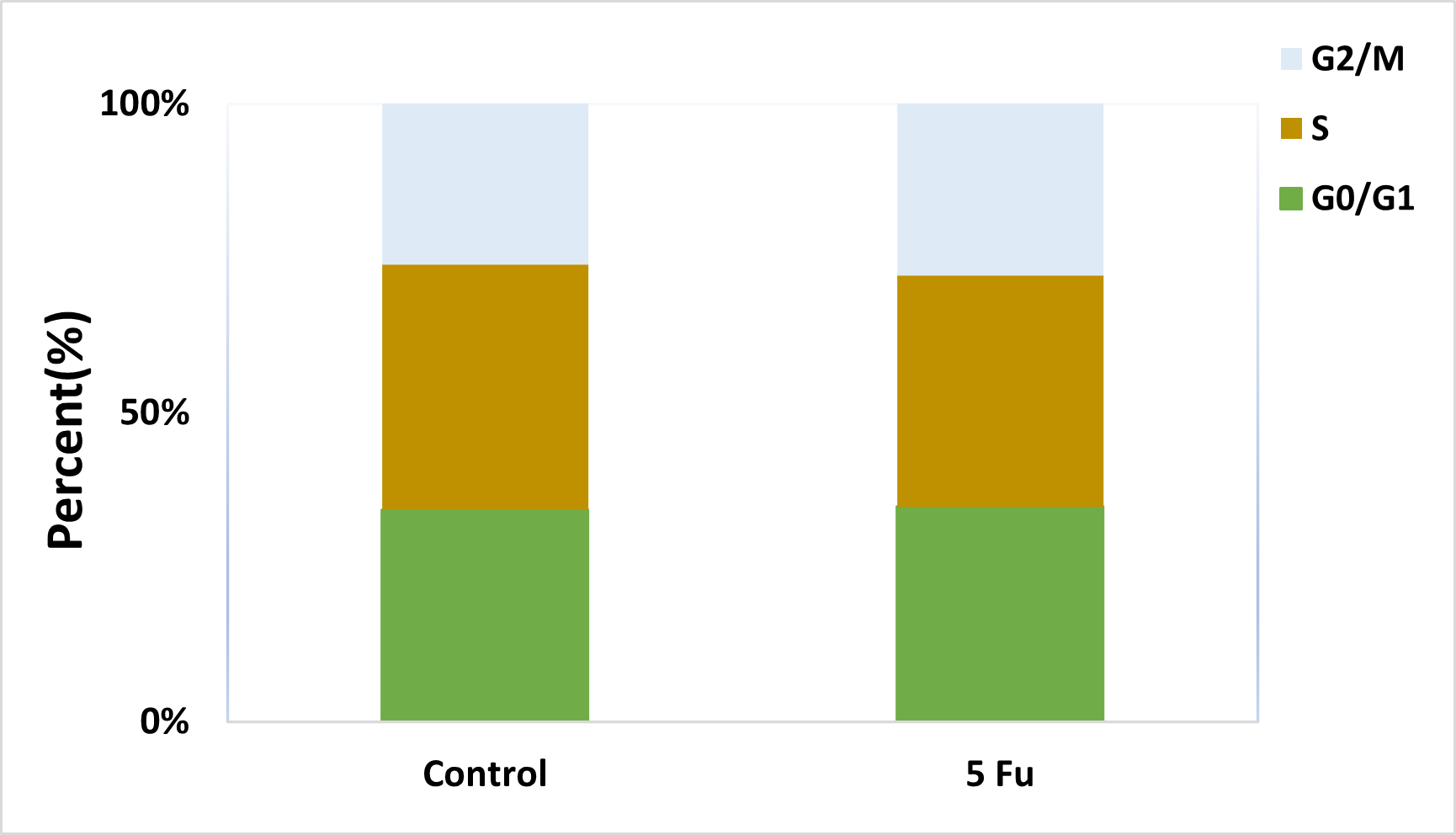


**Figure S5.** Cell cycle assessment for compounds **3**, **11** and **5-FU** on SW-620 cells; A, B and C, respectively.

1. **Compound 3**

**
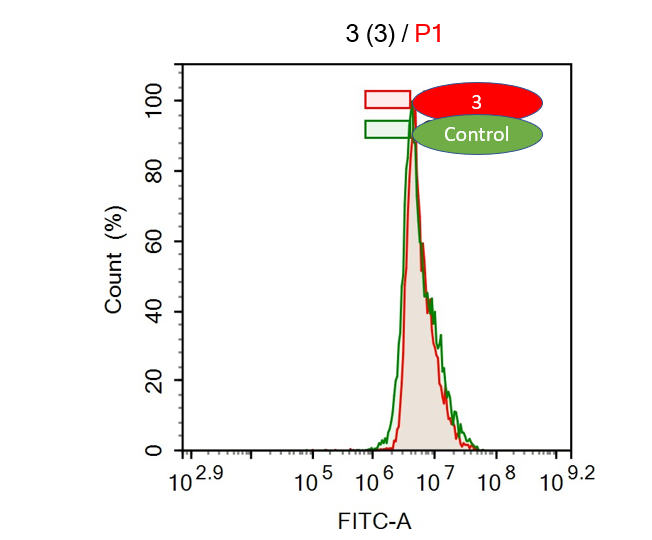
**

1. **Compound 11**


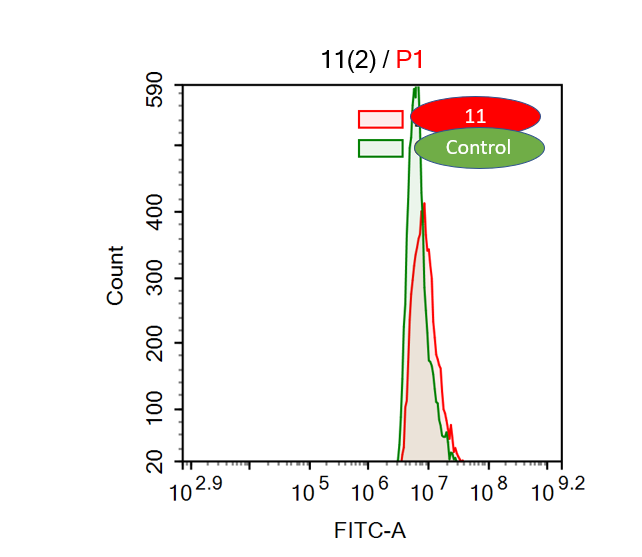


1. **5-FU**

**
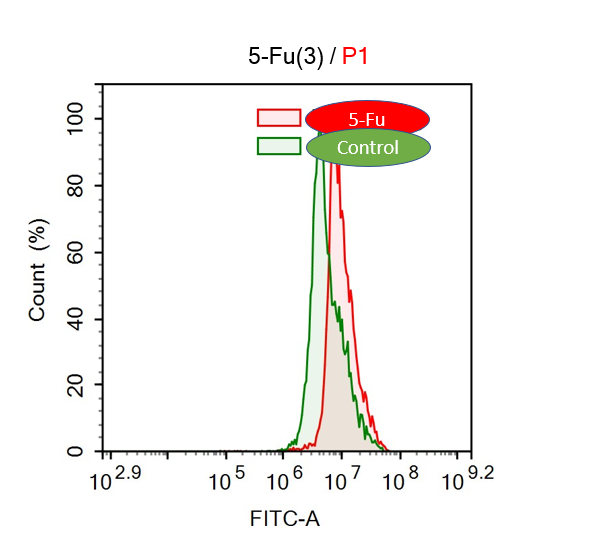
**

**Figure S6.** Autophagy assessment for compounds **3**, **11** and **5-FU** on HT-29 cells; A, B and C, respectively.

1. **Compound 3**

**
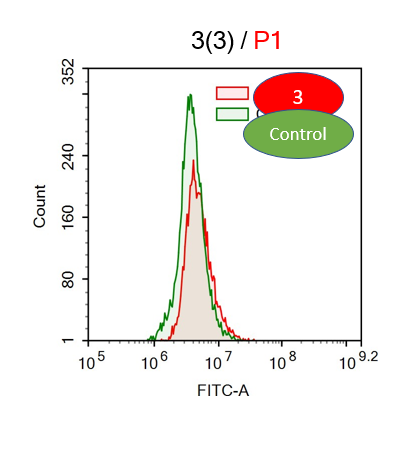
**

1. **Compound 11**

**
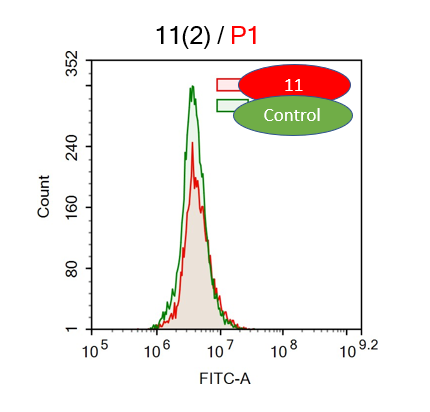
**

1. **5-FU**

**
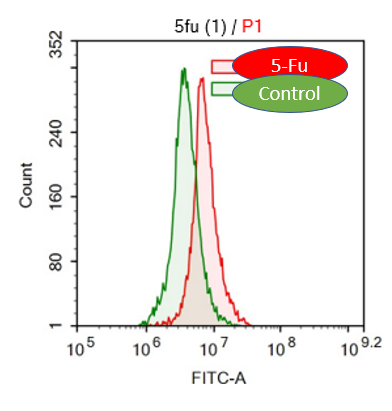
**

**Figure S7.** Autophagy assessment for compounds **3**, **11**, and **5-FU** on SW-620 cells; A, B and C, respectively.

**Figure S8.** Dose response curves for compounds **3**, **11** and **5-FU** on normal cells (OEC).

1. **SW-620**

**A. Control Compound 3 Compound 11**

| 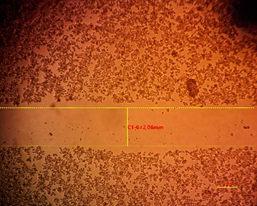 | 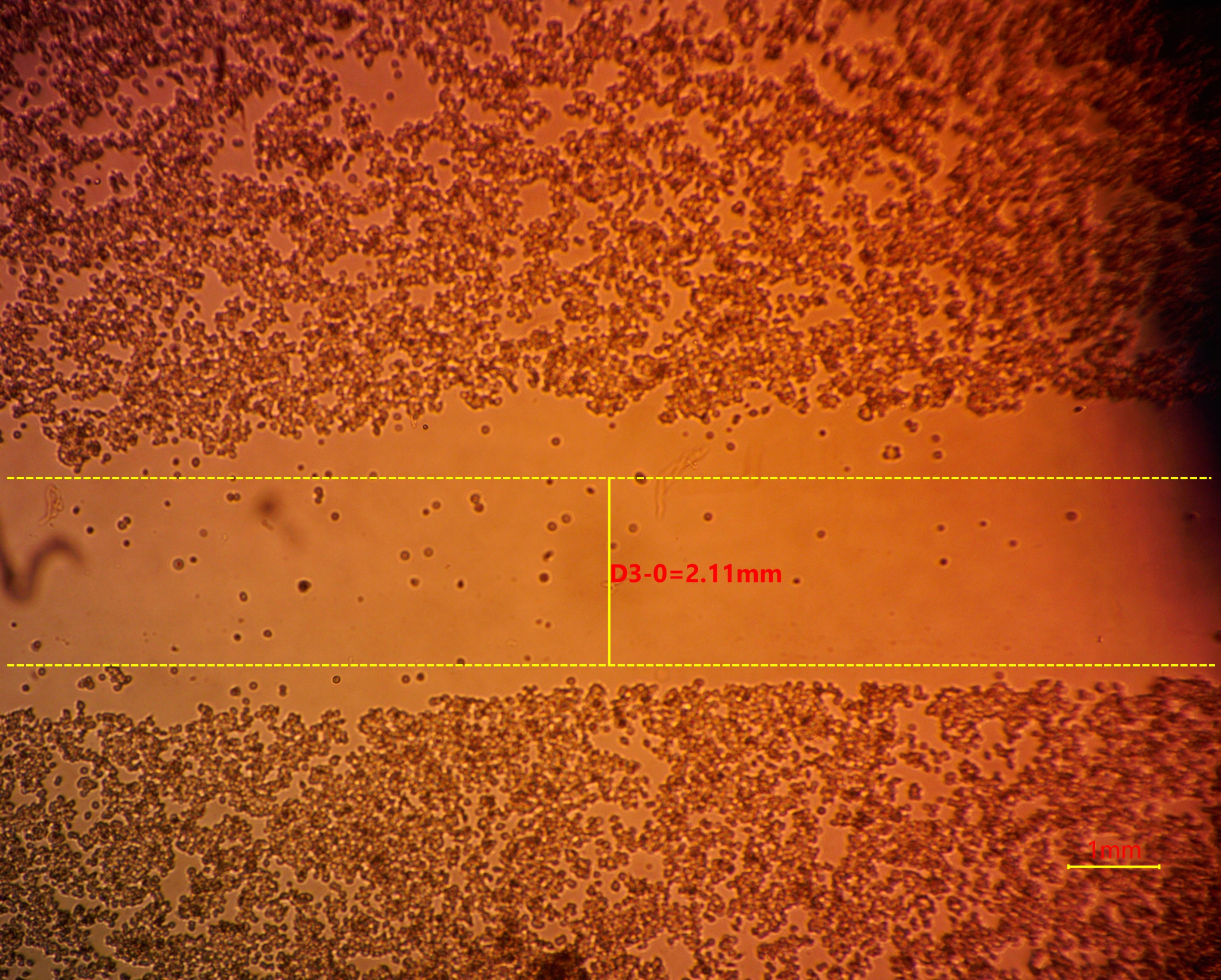 | 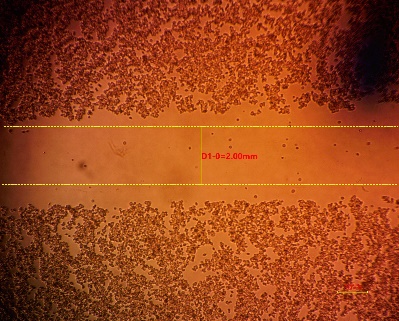 |  |
| --- | --- | --- | --- |
| 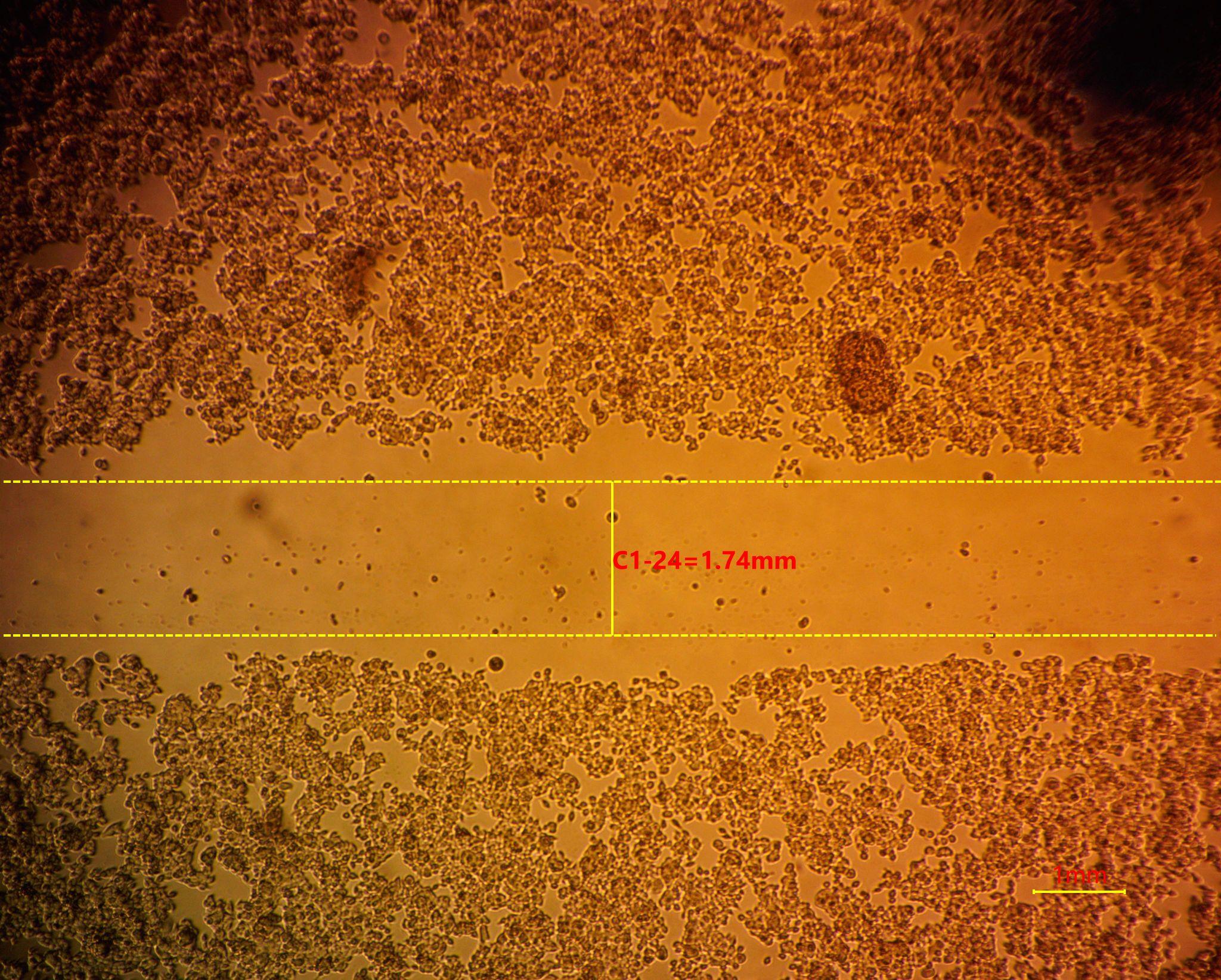 | 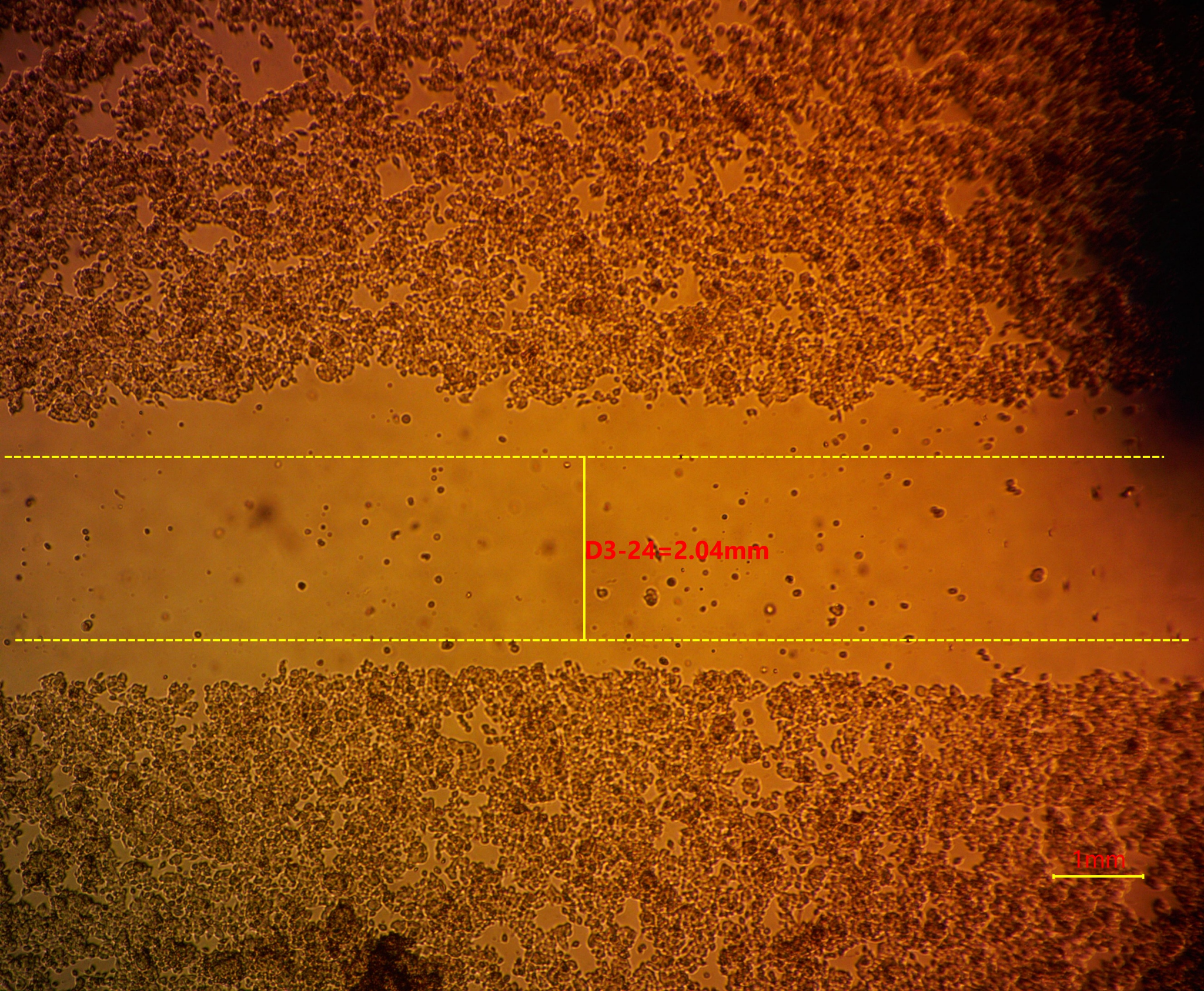 | 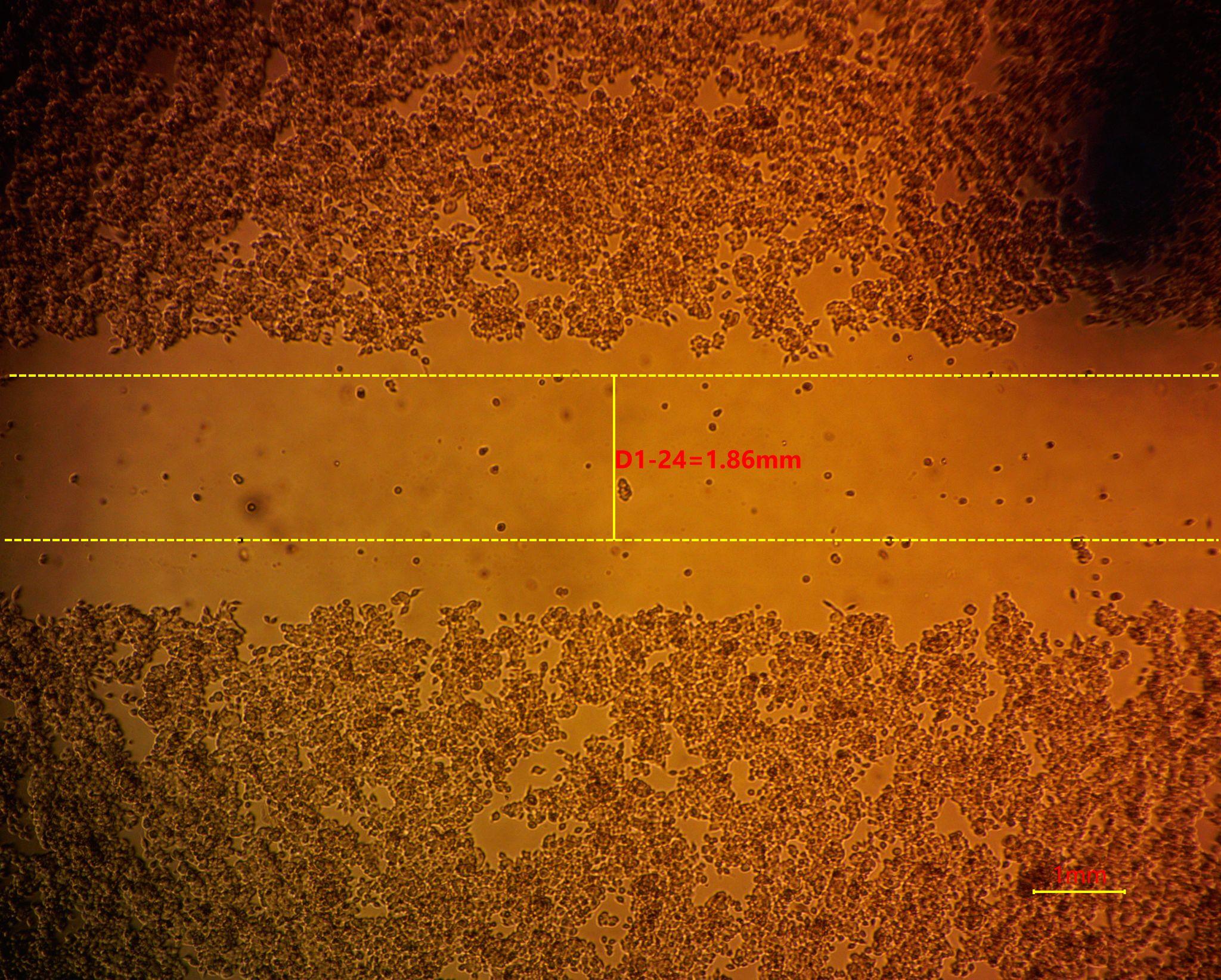 |  |
| 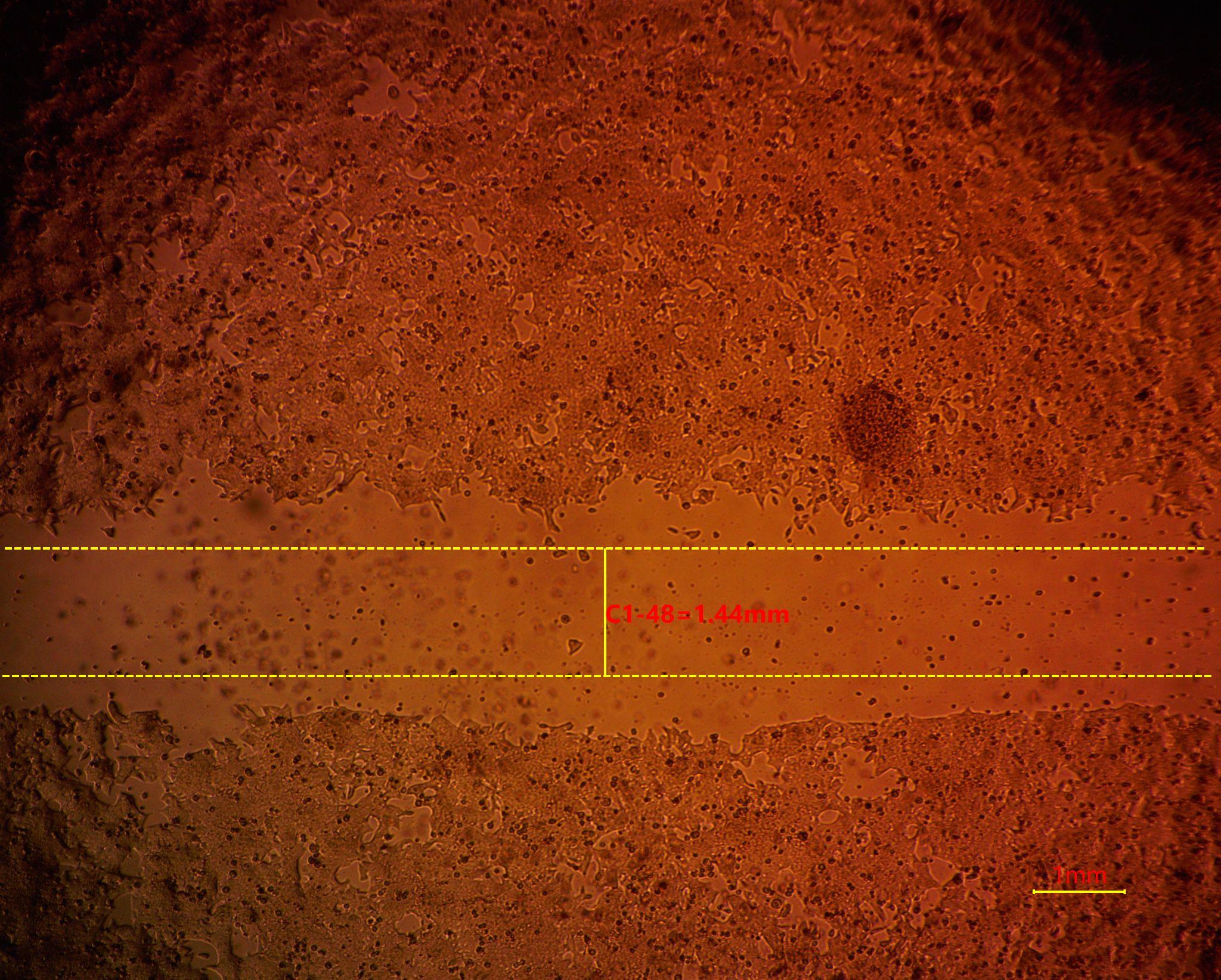 | 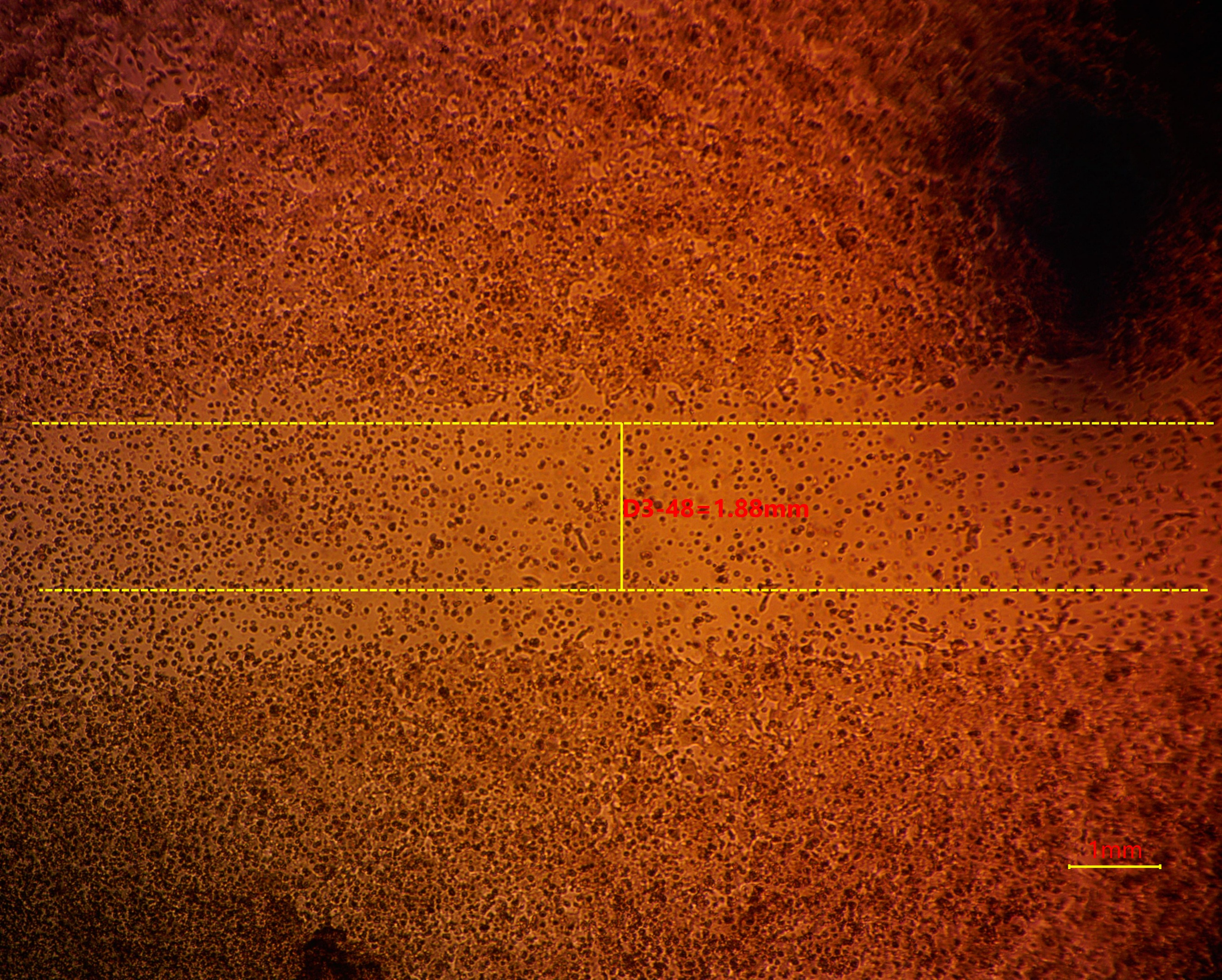 | 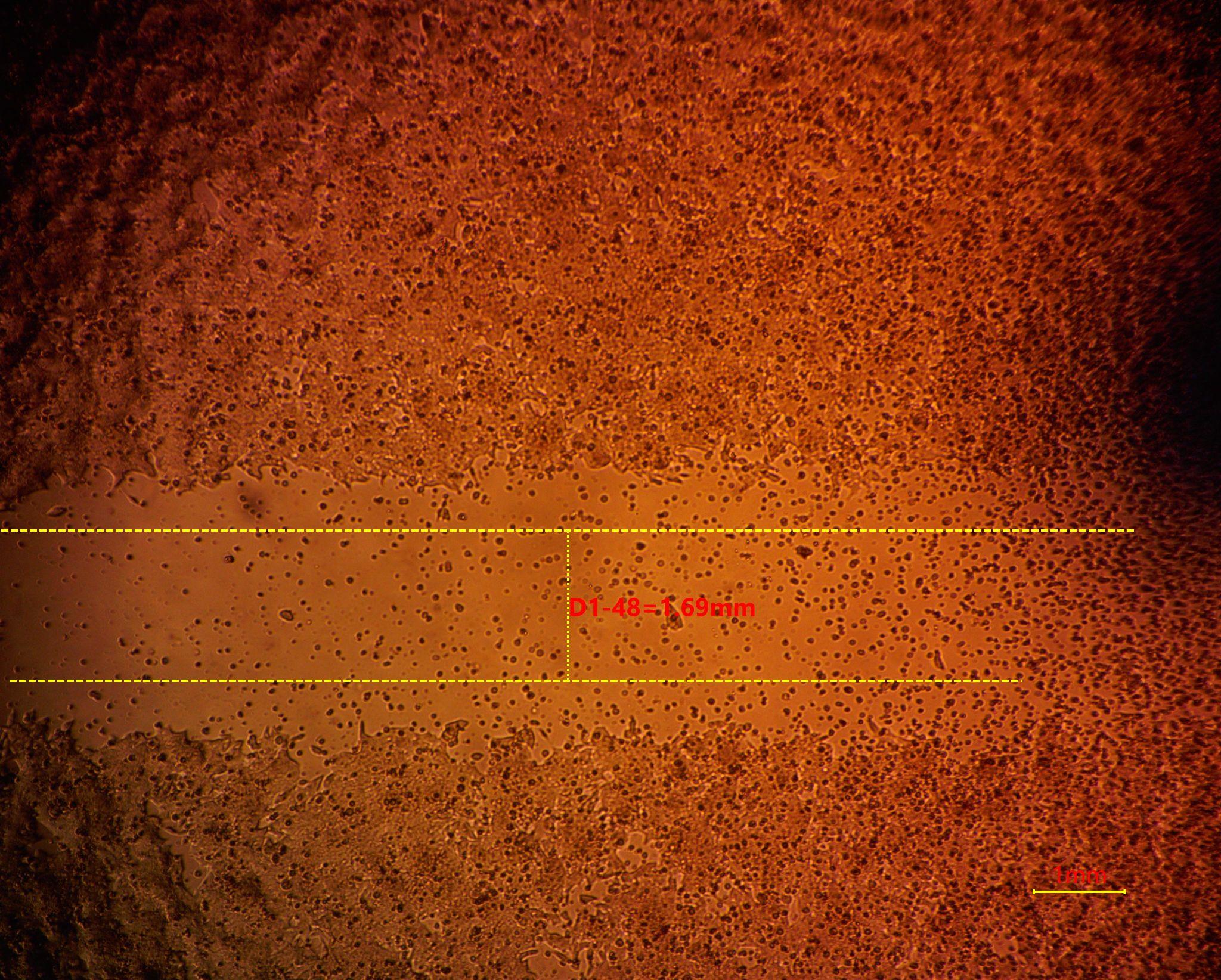 | |
| 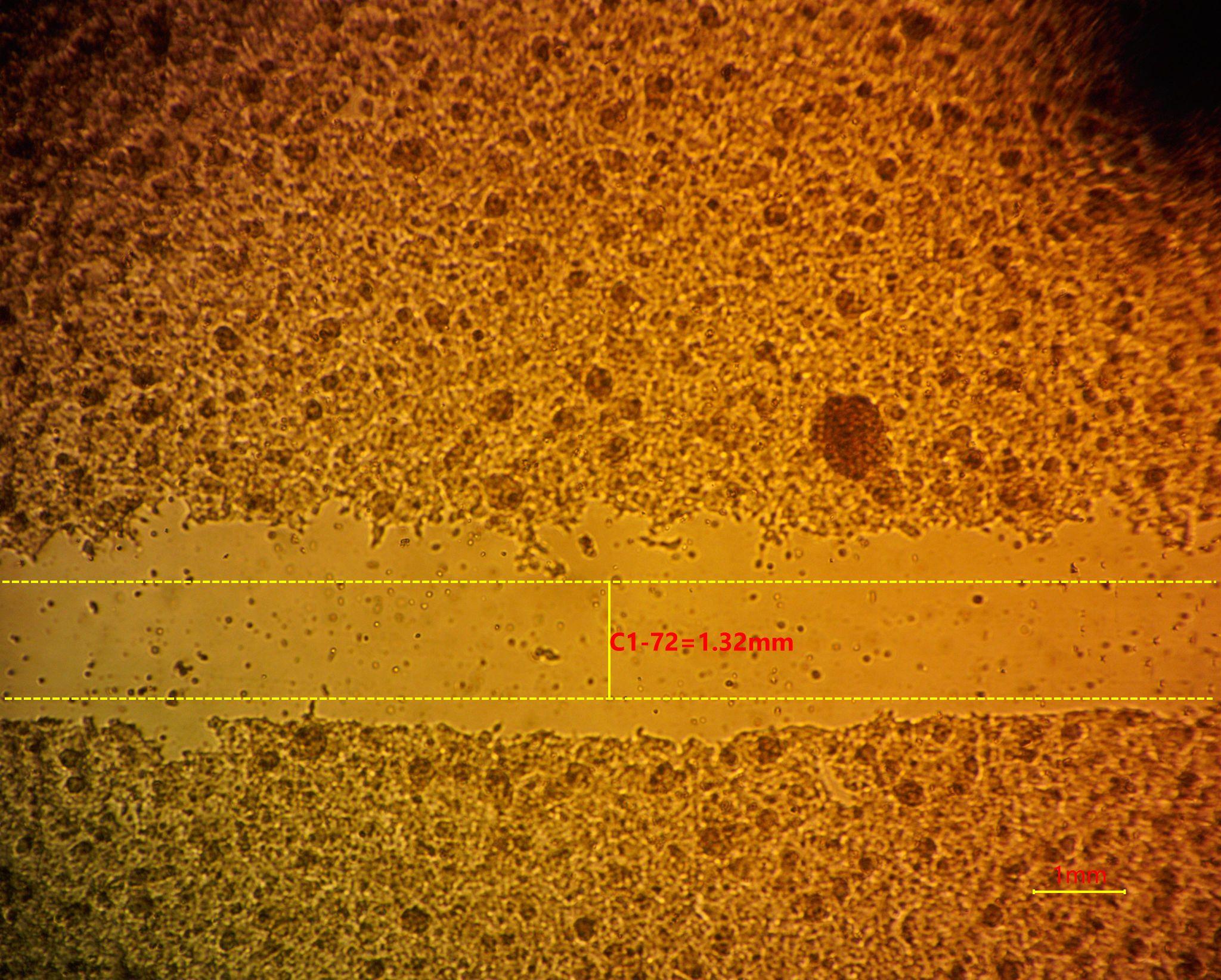 | 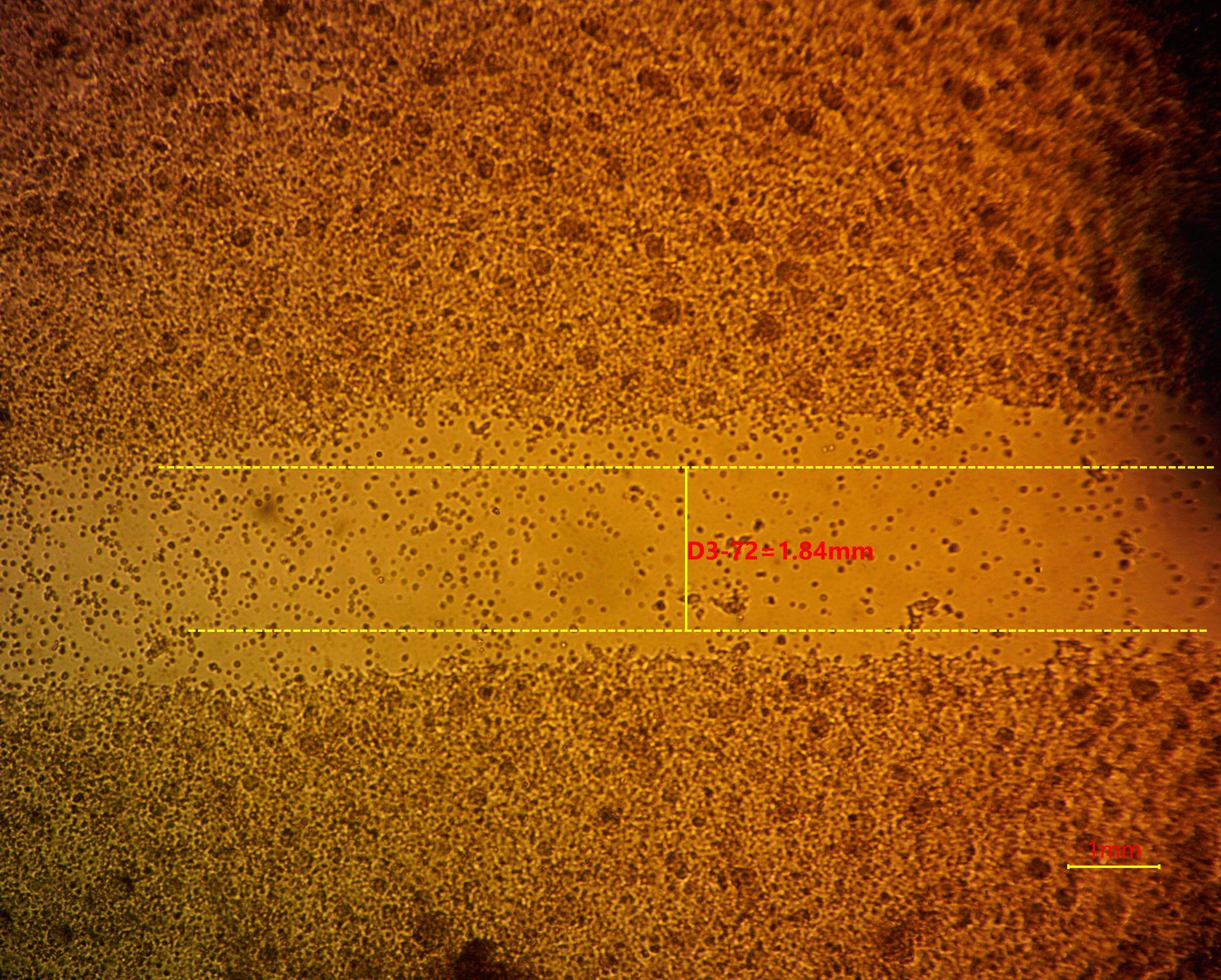 | 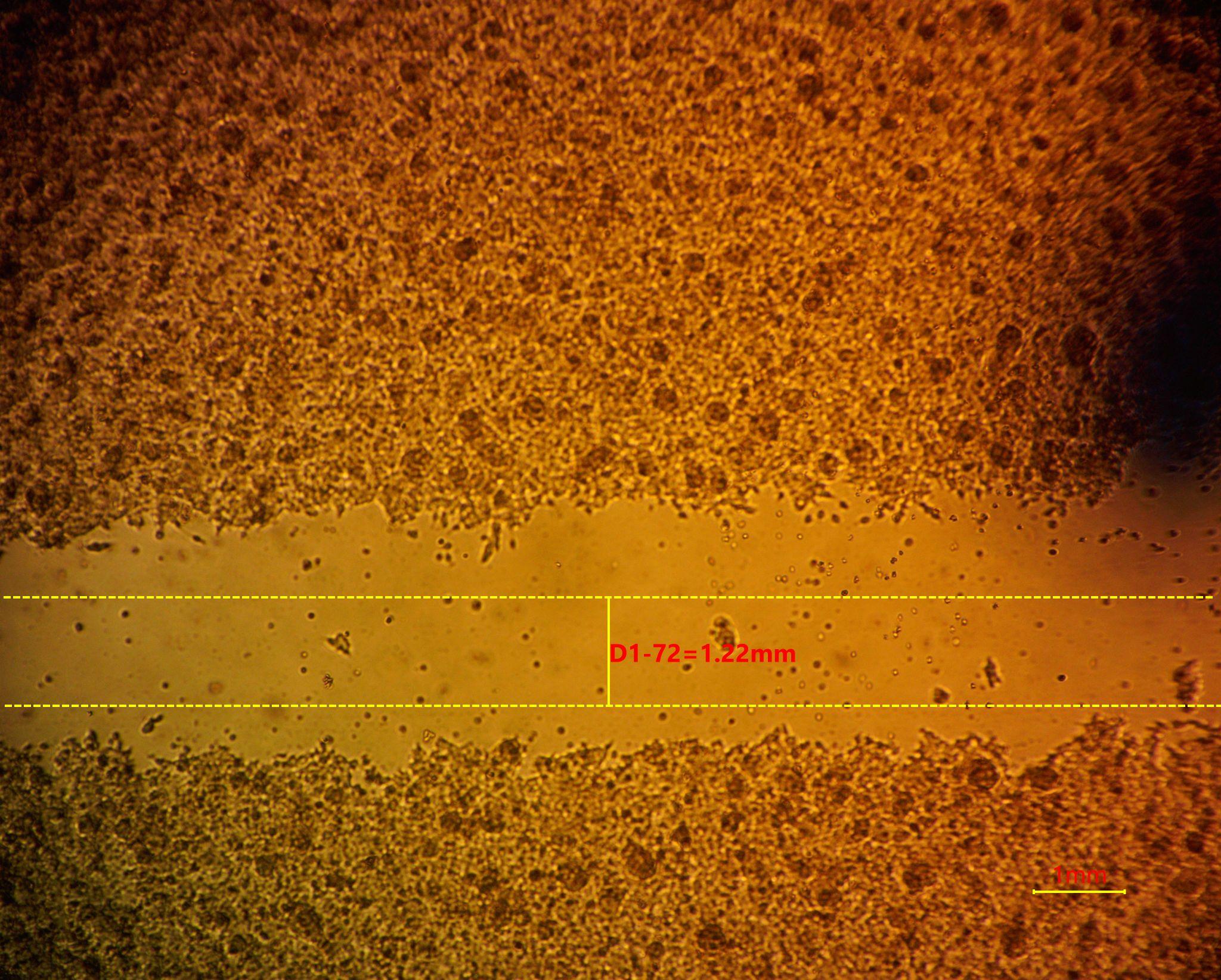 | |

**B.**


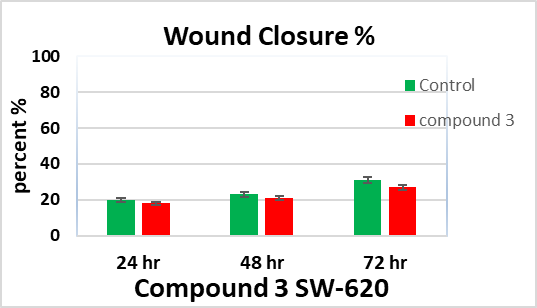

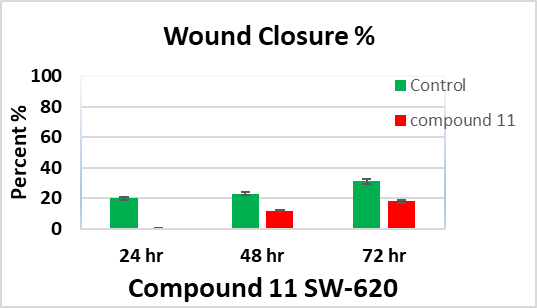


**Figure S9A, B**: Wound closure percentage for both compounds **3** and **11** after three-time intervals, 24, 48, and 72 hrs on SW-620.


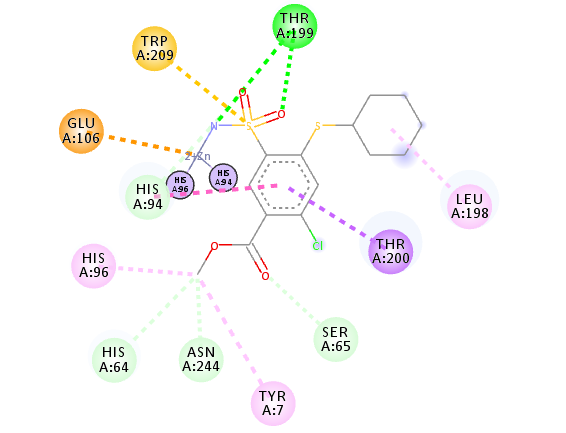


**A)**


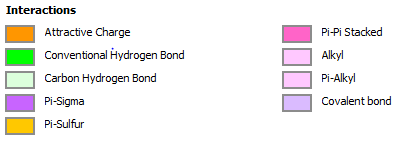


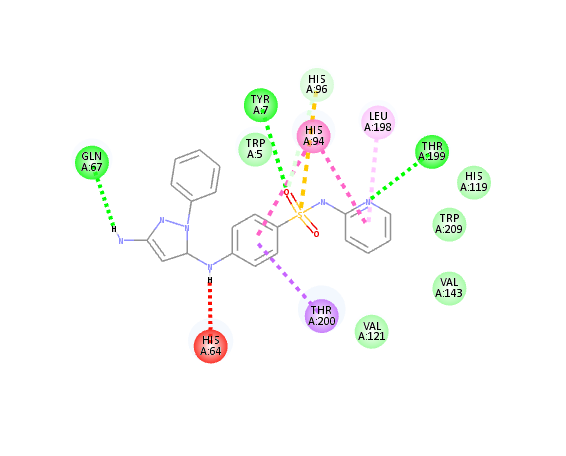


**B)**


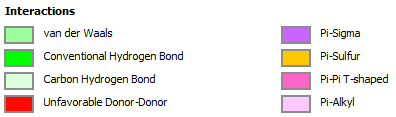


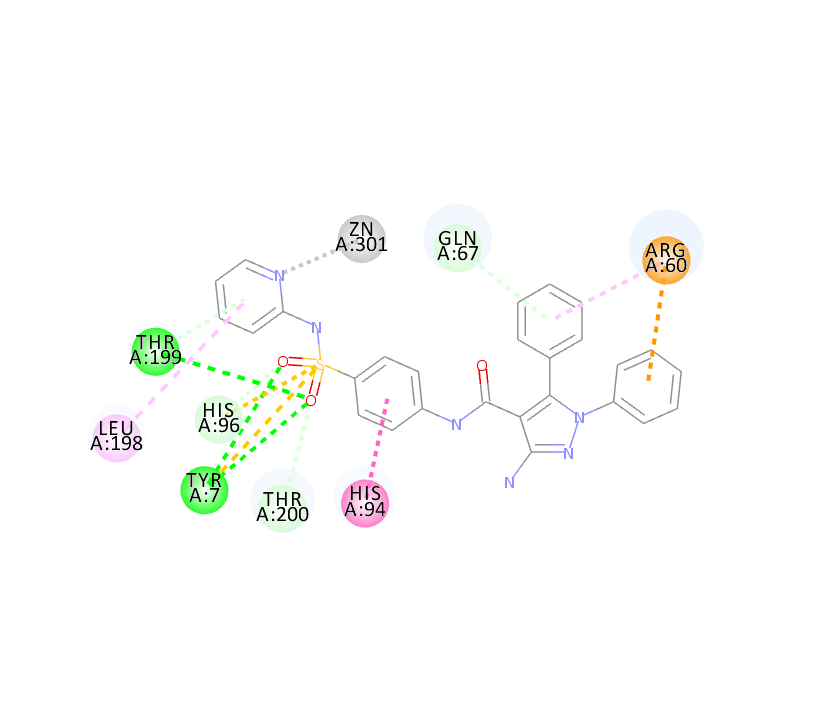


**C)**


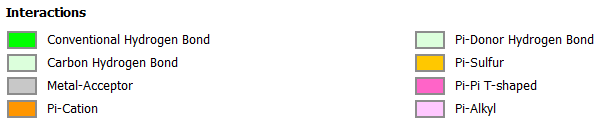


**Figure S10.** (**A**) 2D interaction diagram of sulfonamide co-crystallized ligand with CA IX (PDB ID: 7pom). (**B**) 2D interaction diagram of compound **3** with CA IX (PDB ID: 7pom). (**C**) 2D interaction diagram of compound **11** with CA IX (PDB ID: 7pom).

**A.**

**
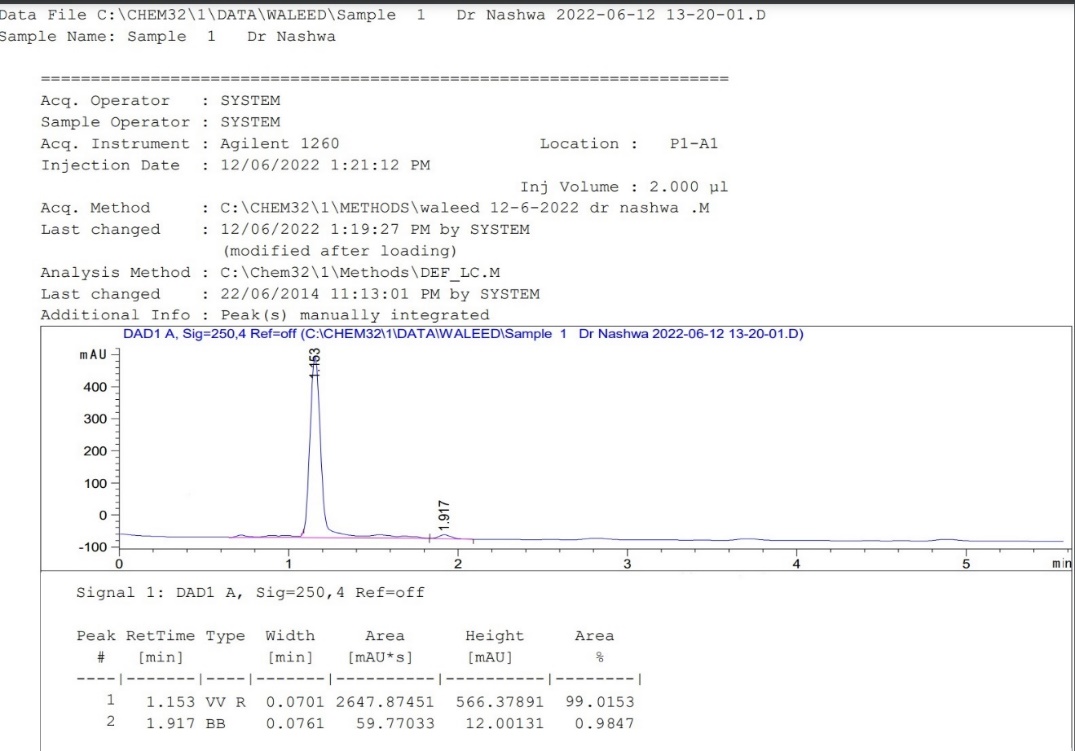
**

**B.**

**
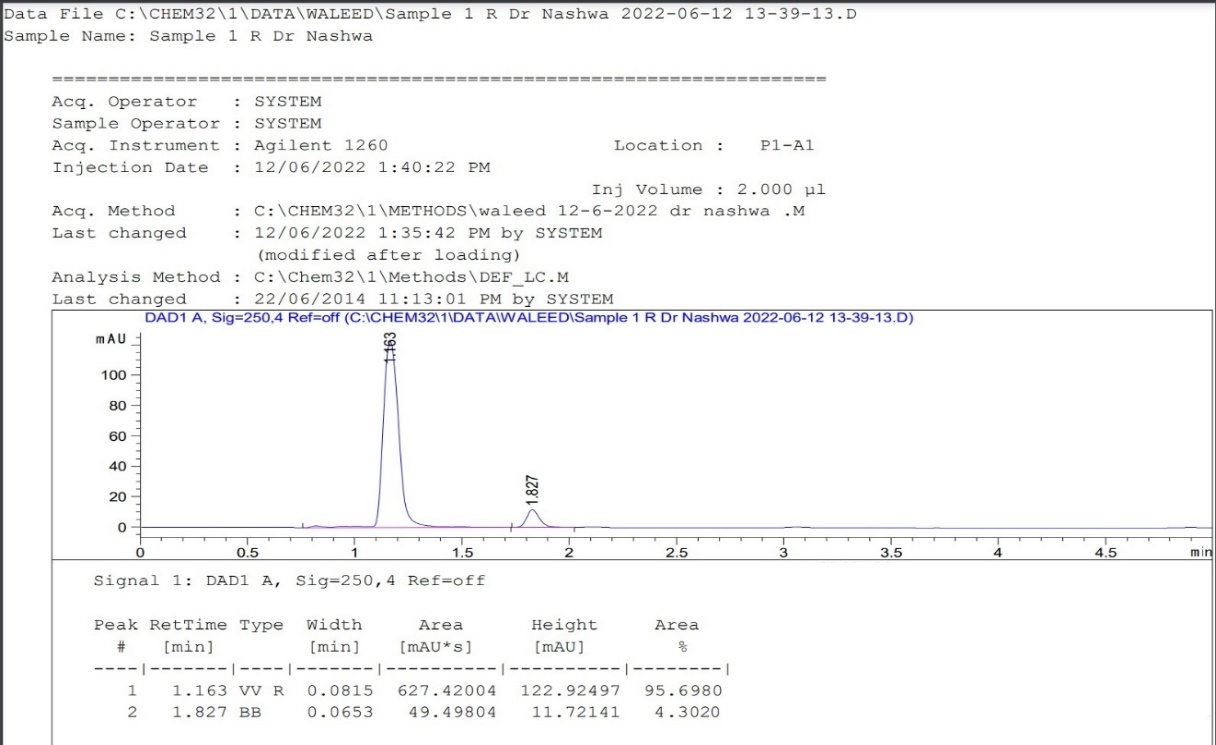
**

**Figure S11. A, B:** Compound **11** HPLC purity analysis before and after radiation respectively.

**Compound 2 ^1^H-NMR**

**
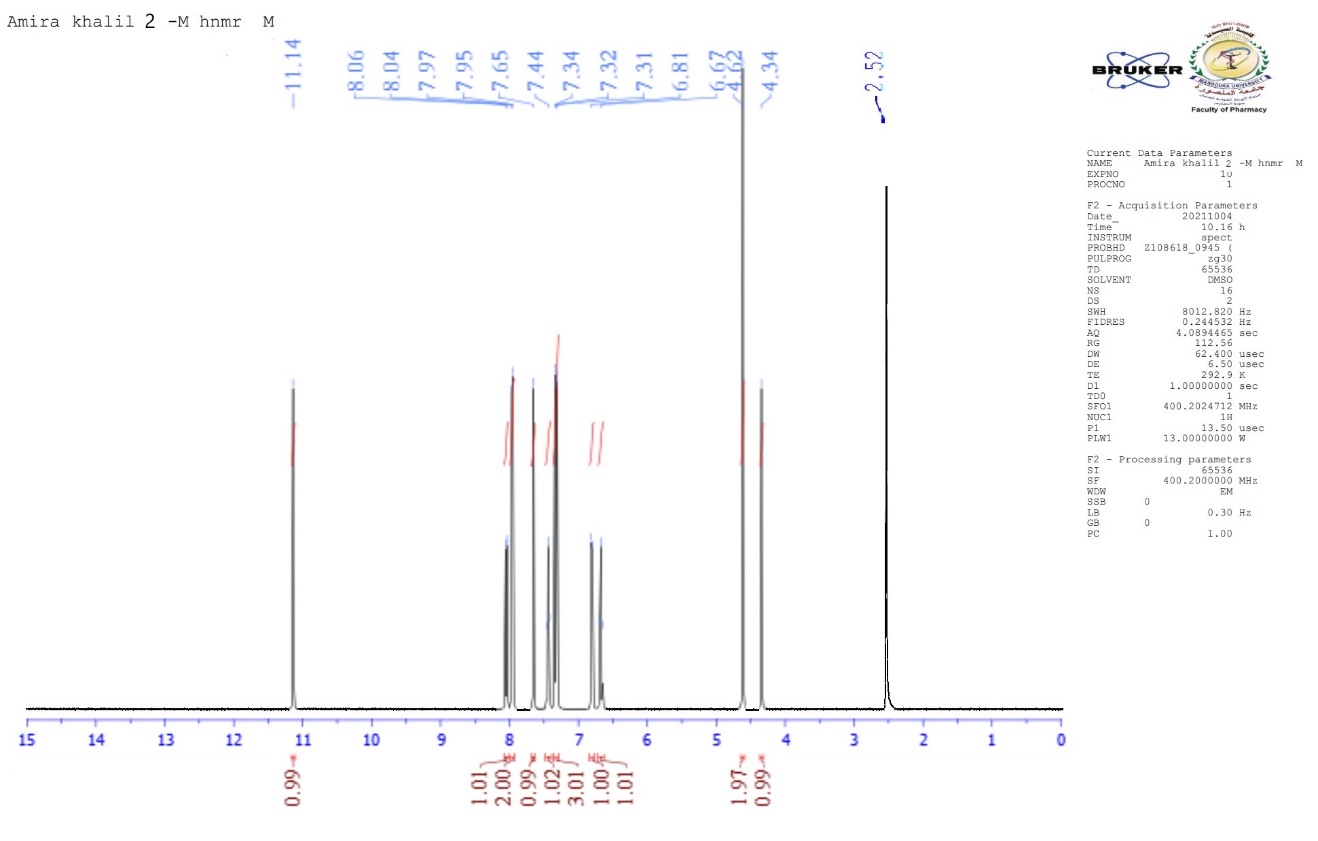
**

**
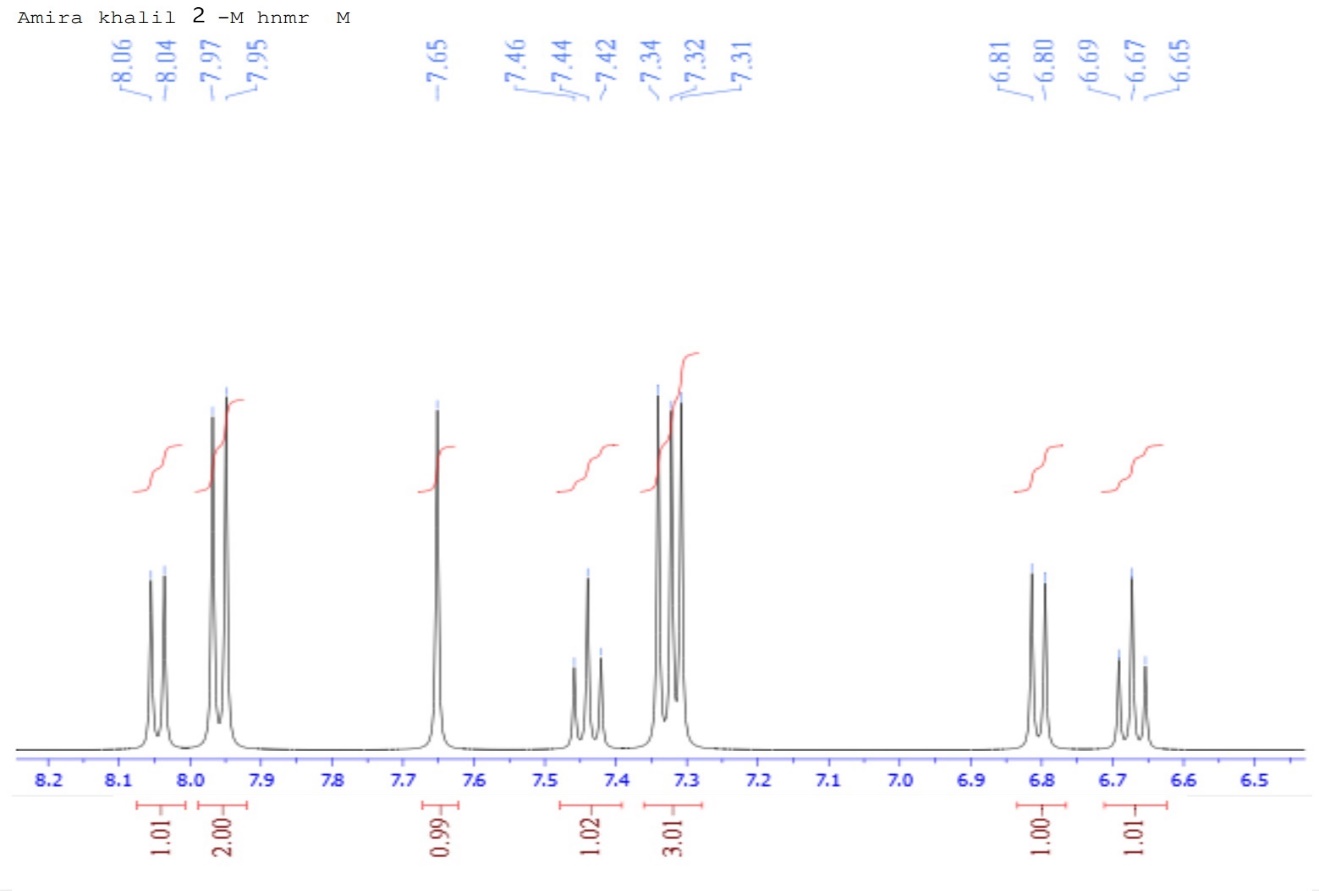
**

**Compound 2 ^1^H-NMR-D2O**


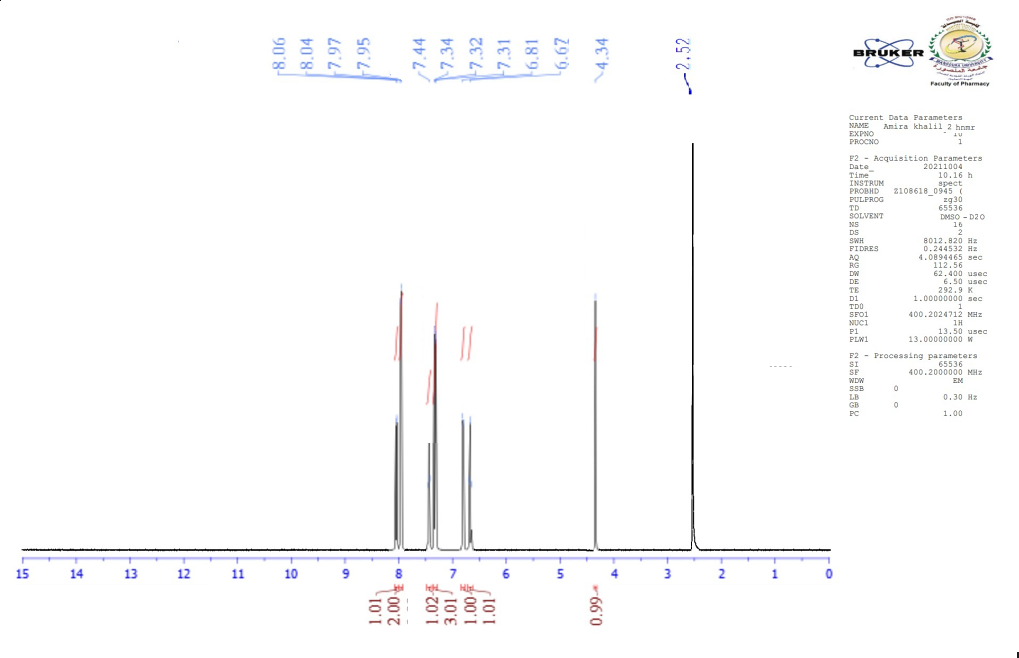


**Compound 2 ^13^C-NMR**


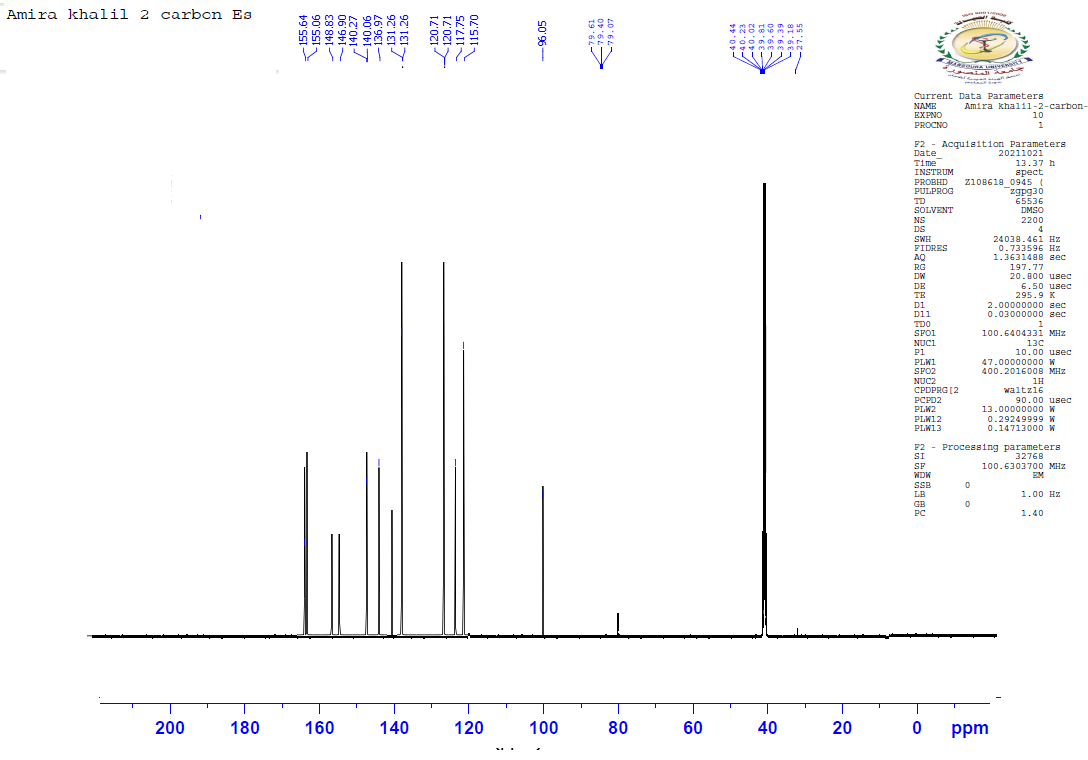


**Compound 3 ^1^H-NMR**


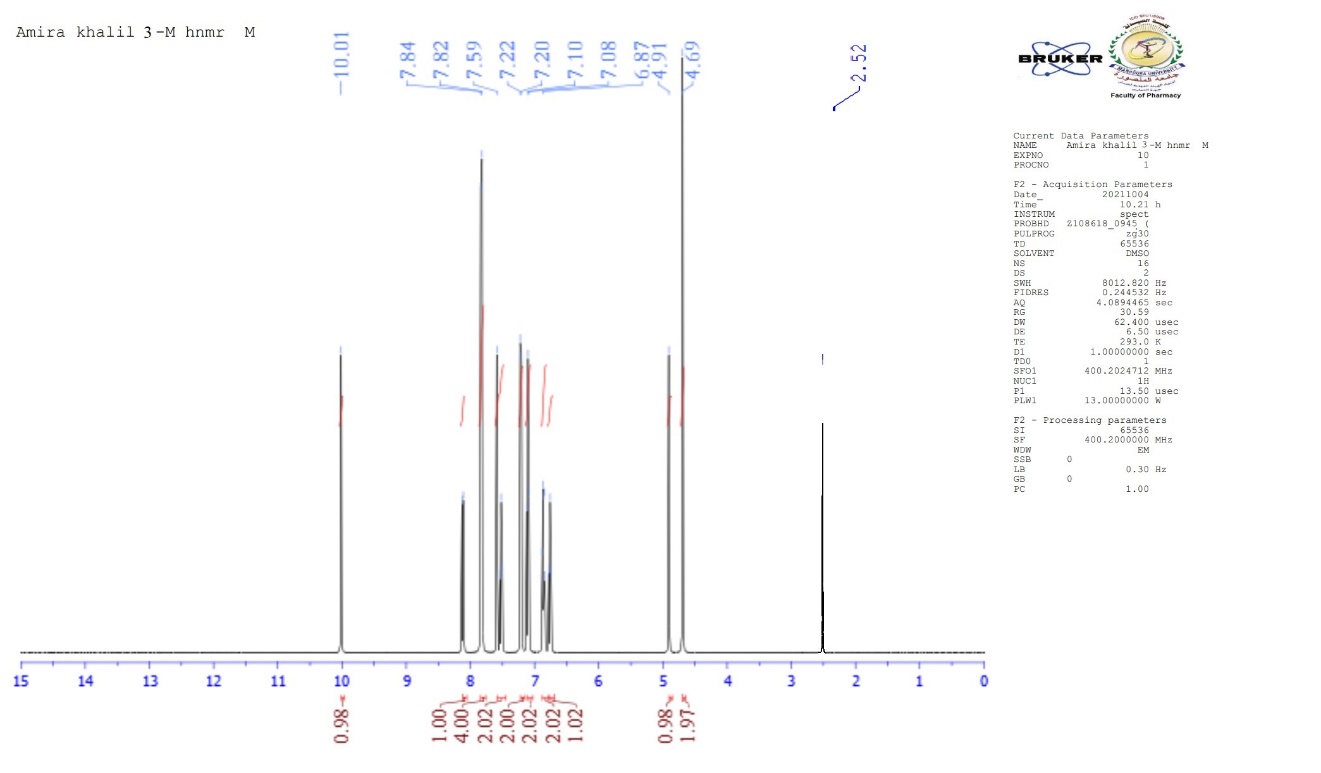


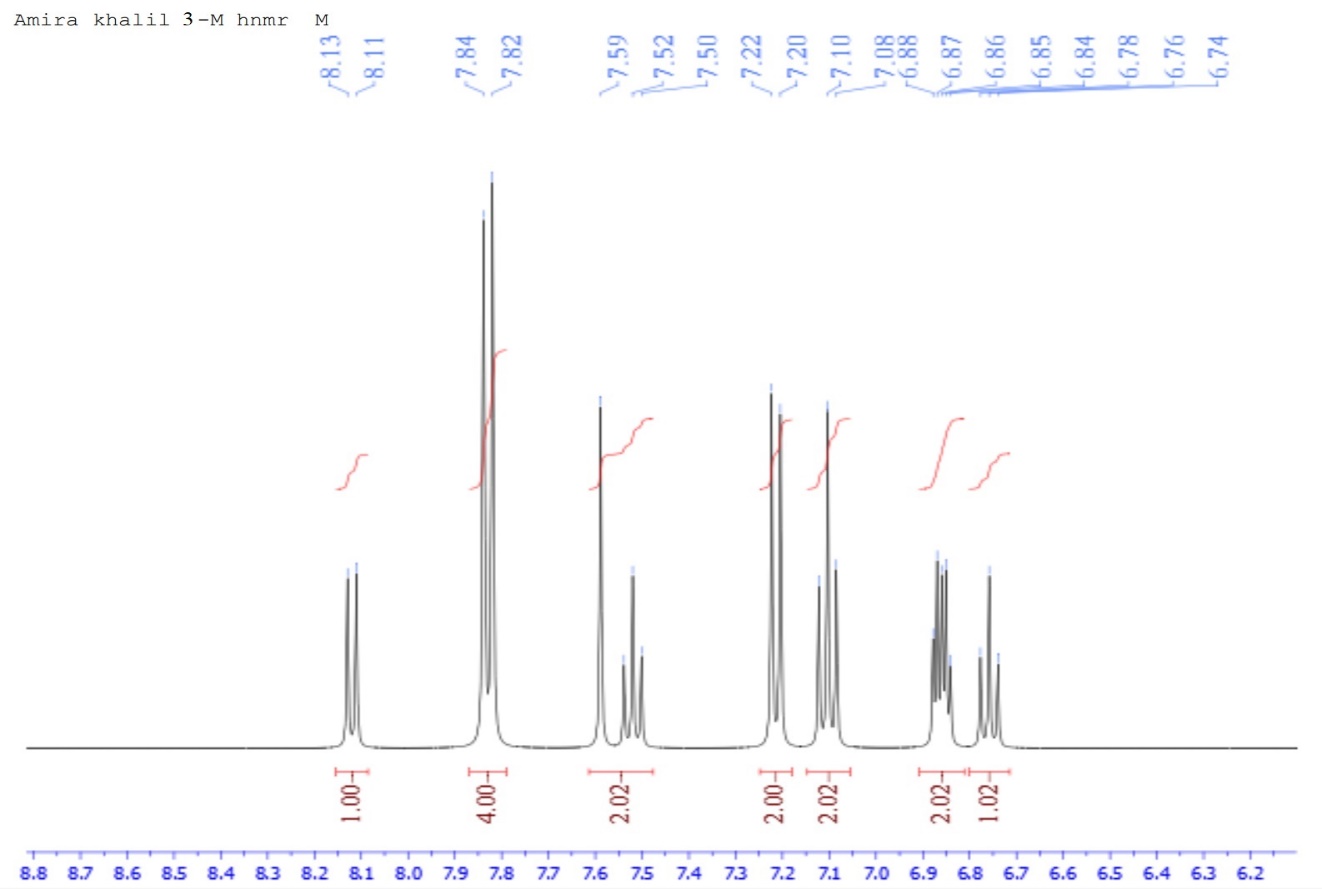


**Compound 3 ^1^H-NMR-D2O**


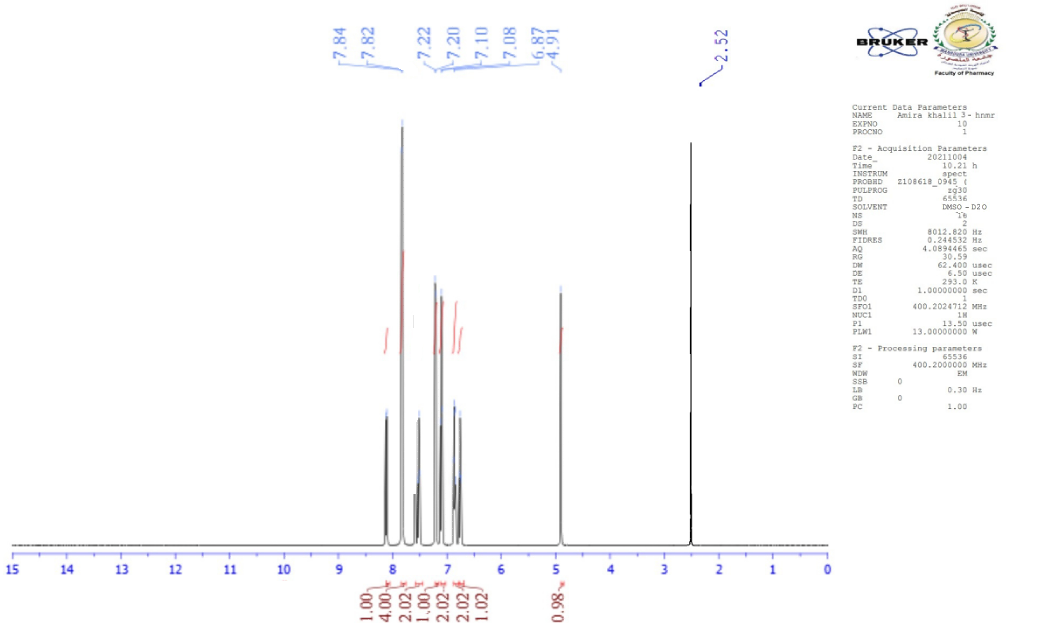


**Compound 3 ^13^C-NMR**


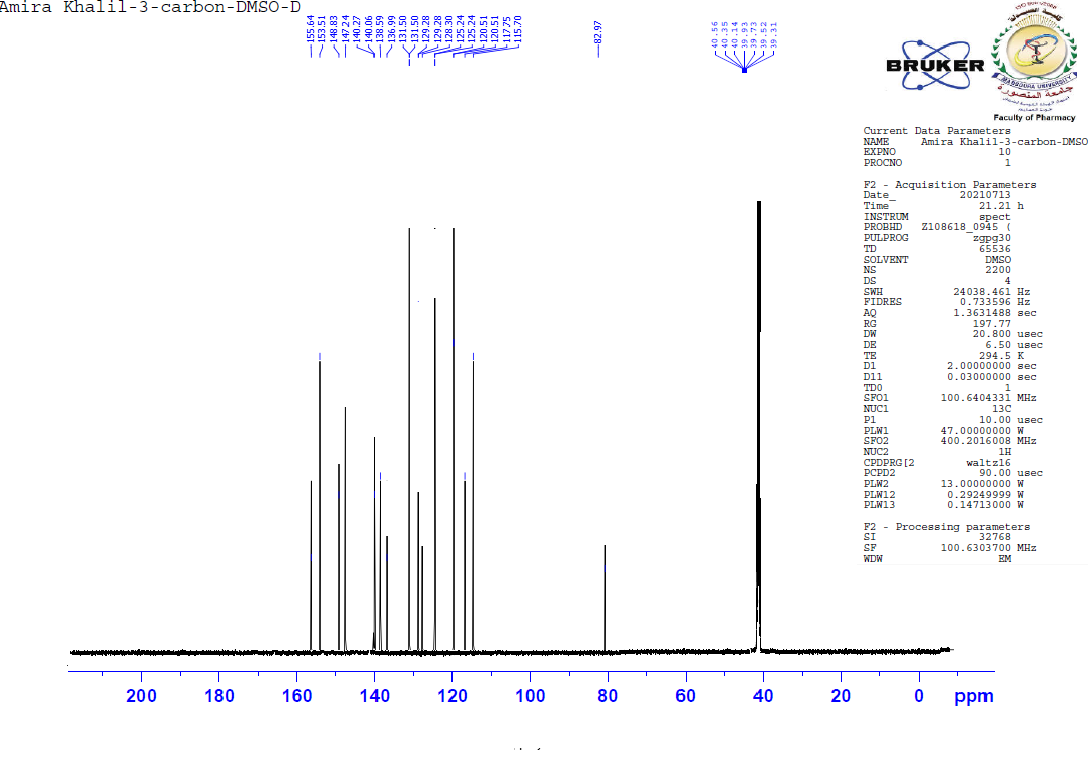


**Compound 4 ^1^H-NMR**


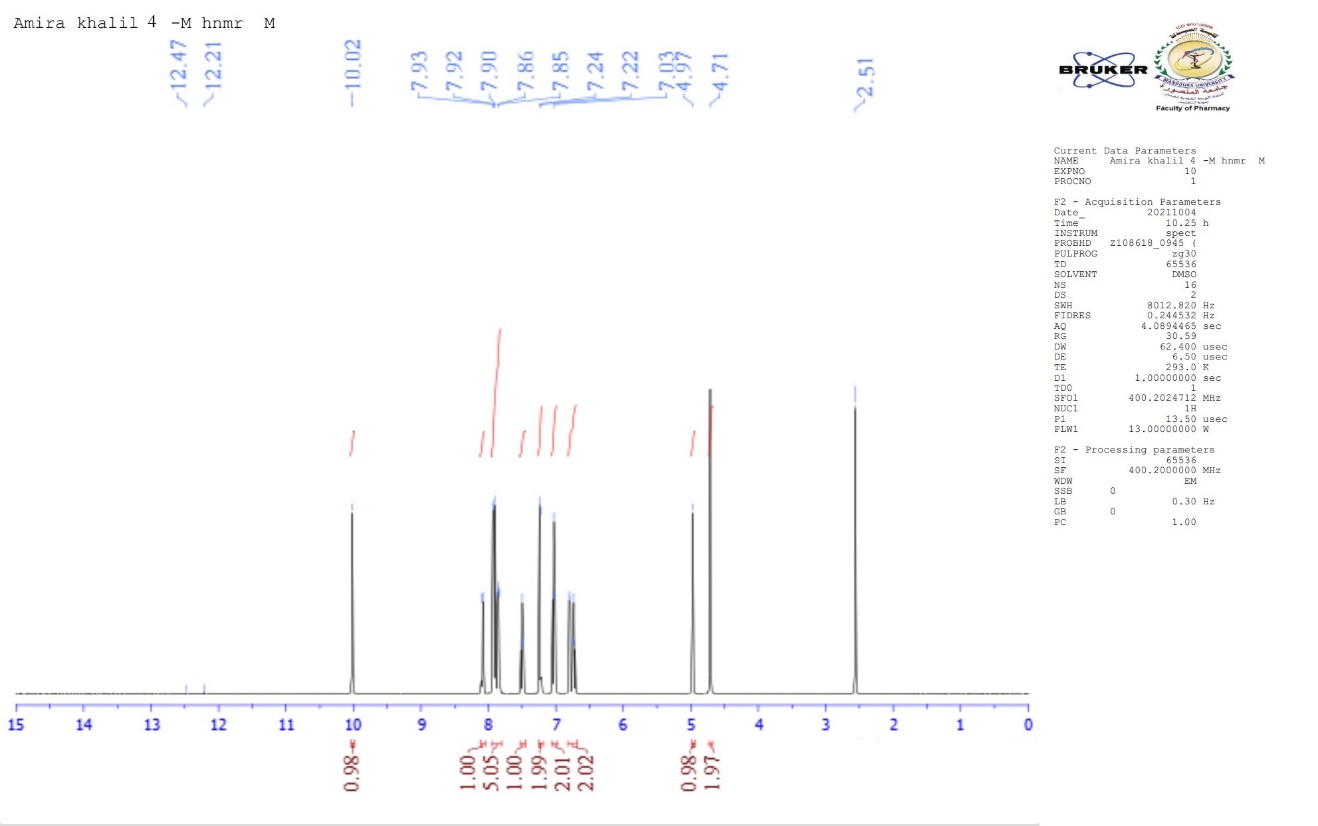


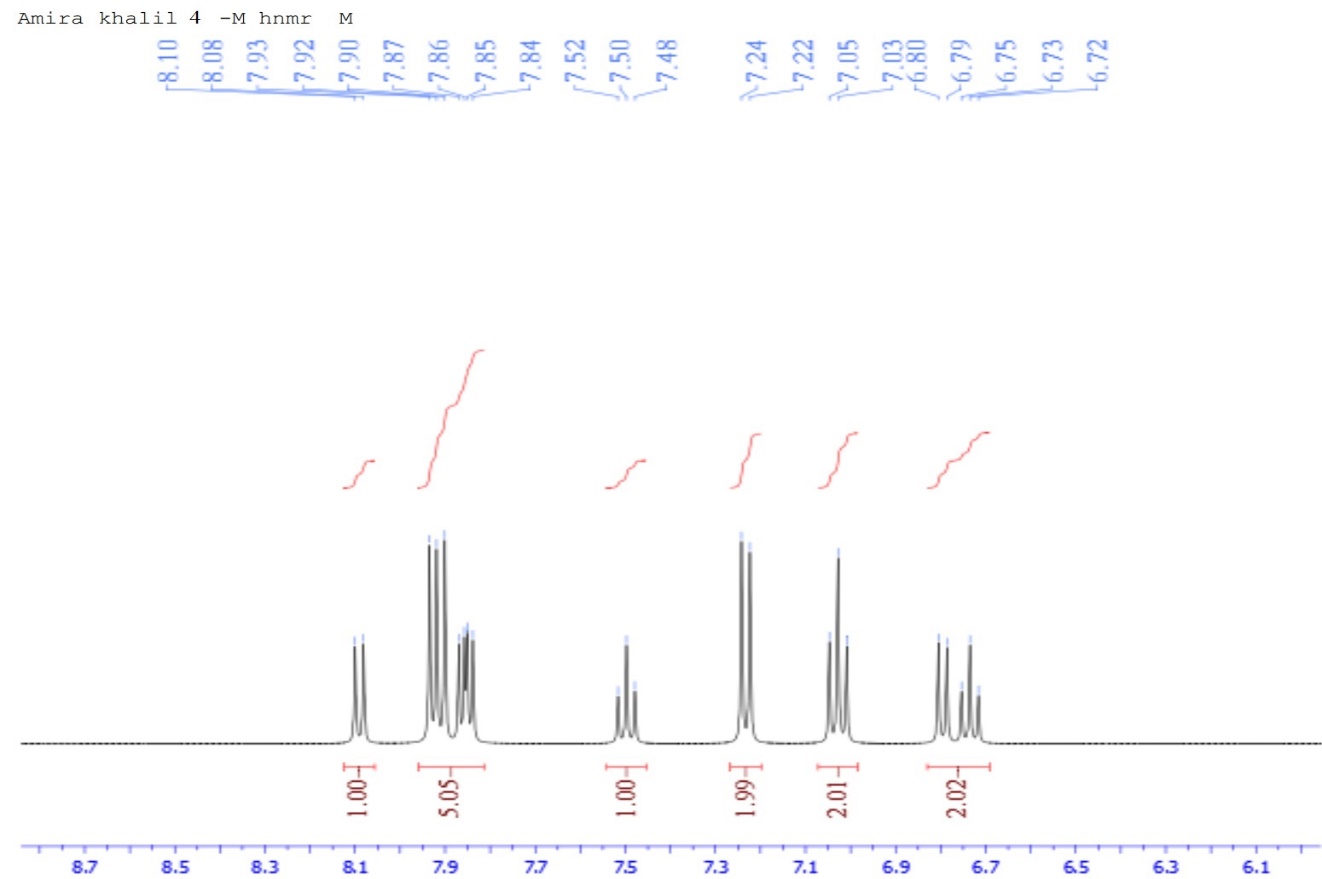


**Compound 4 ^1^H-NMR-D2O**


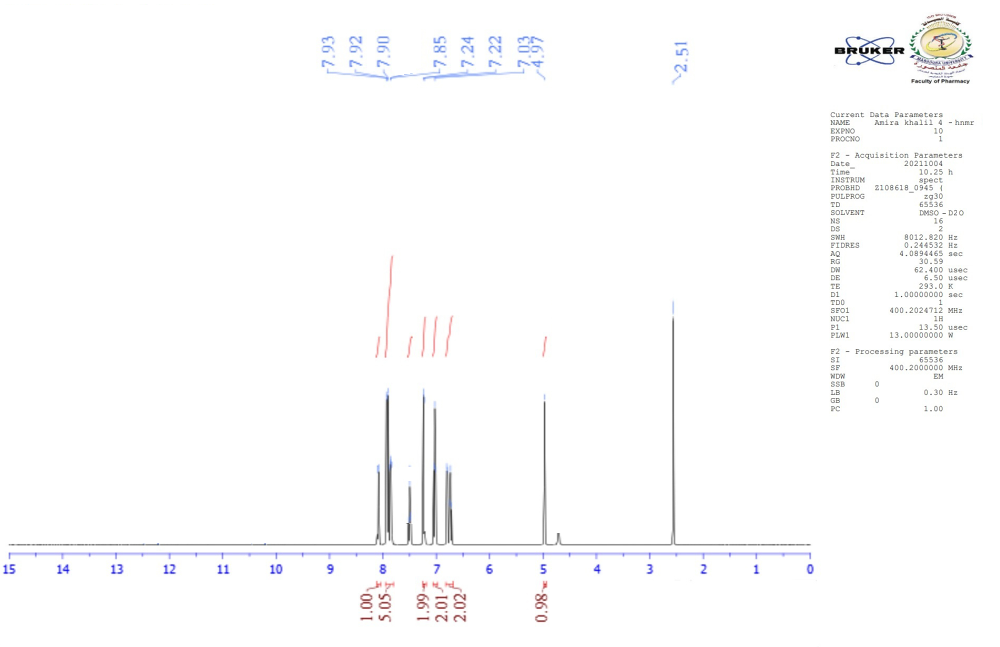


**Compound 4 ^13^C-NMR**


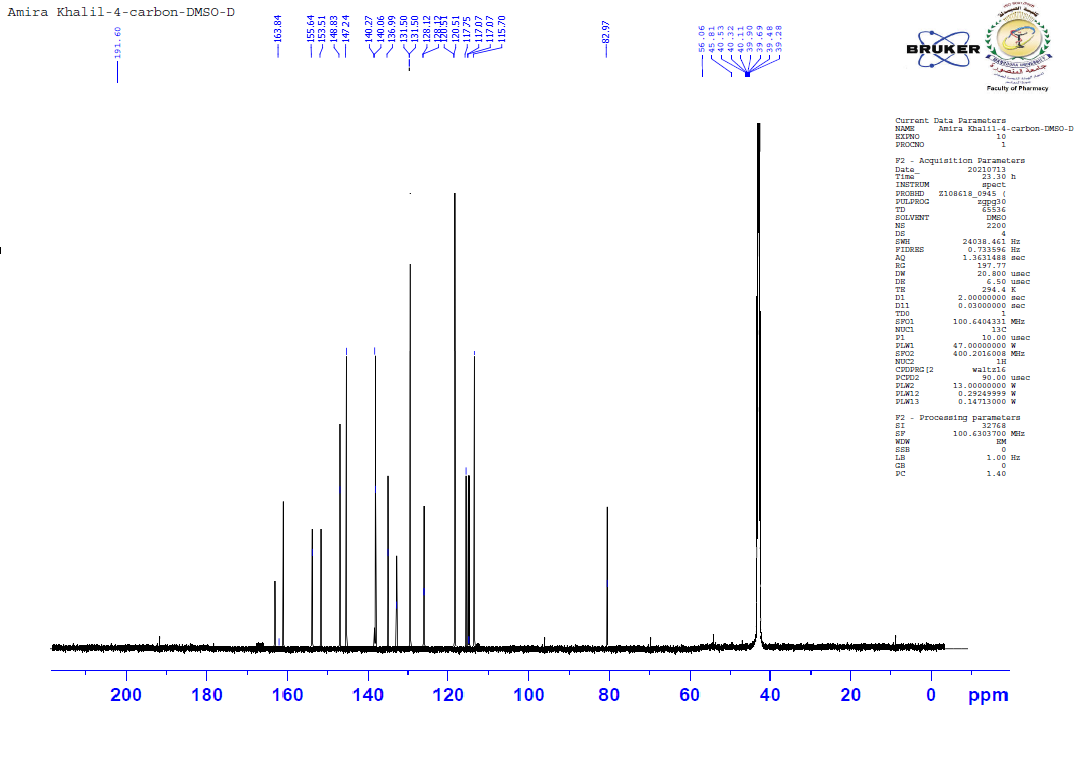


**Compound 5 ^1^H-NMR**


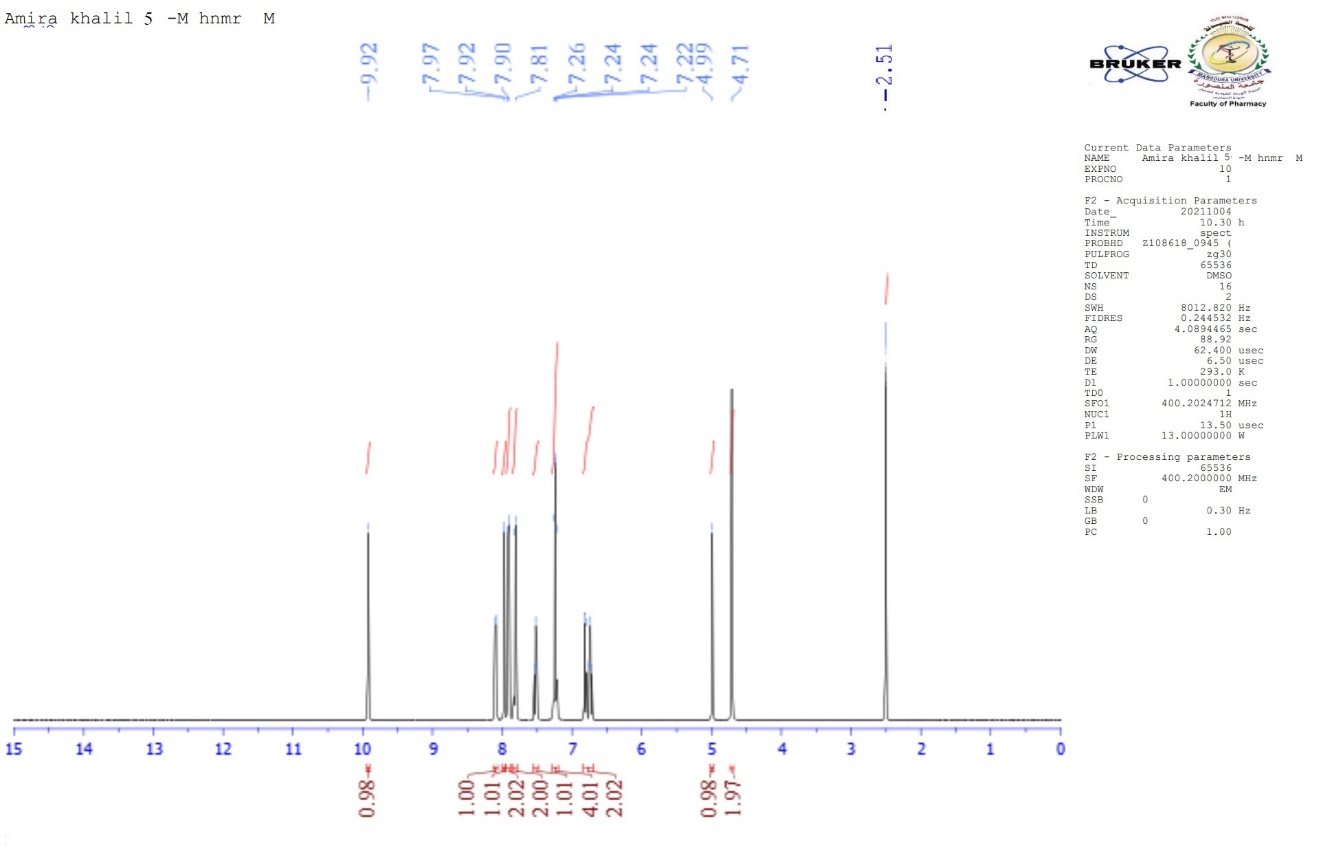


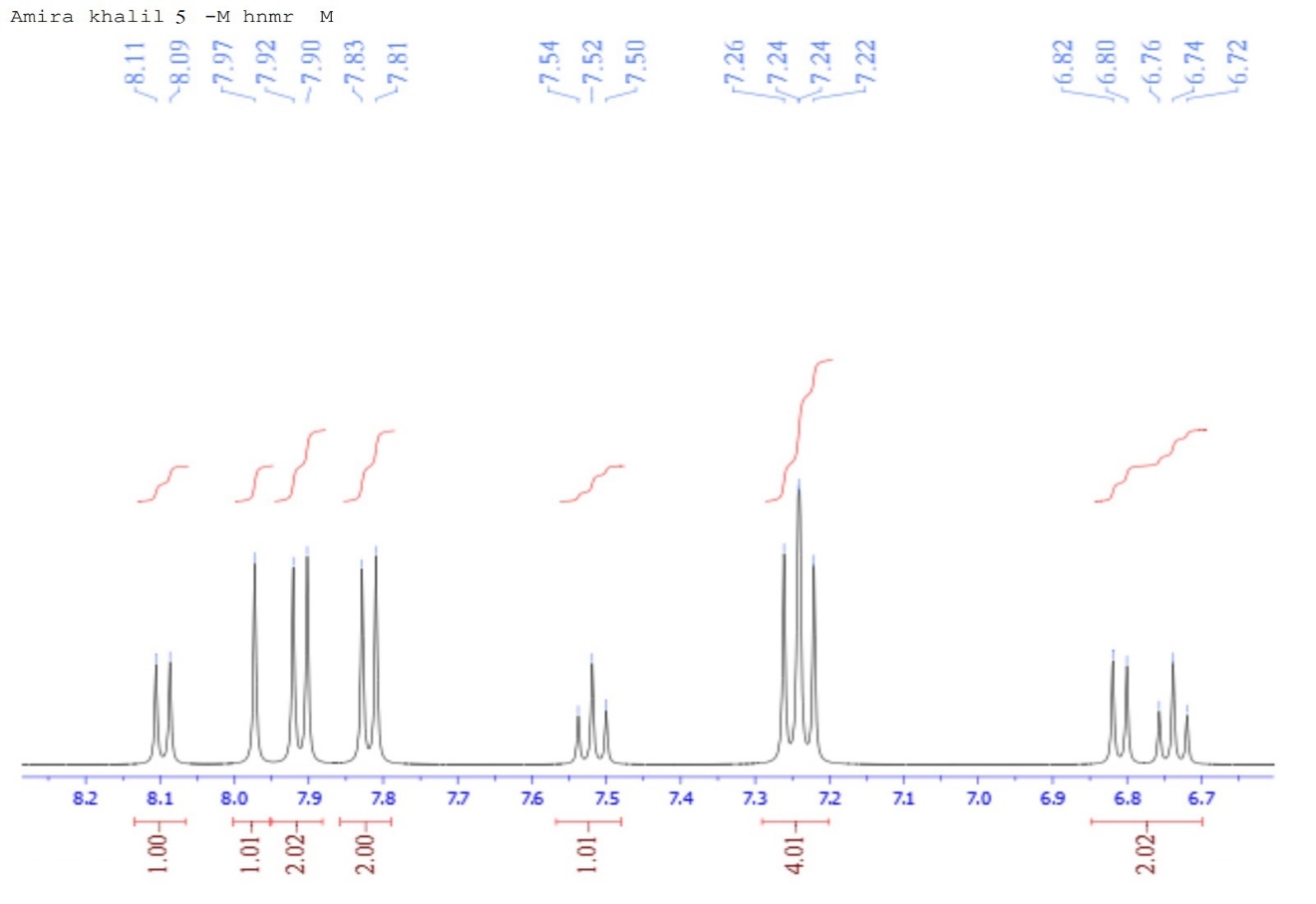


**Compound 5 ^1^H-NMR-D2O**


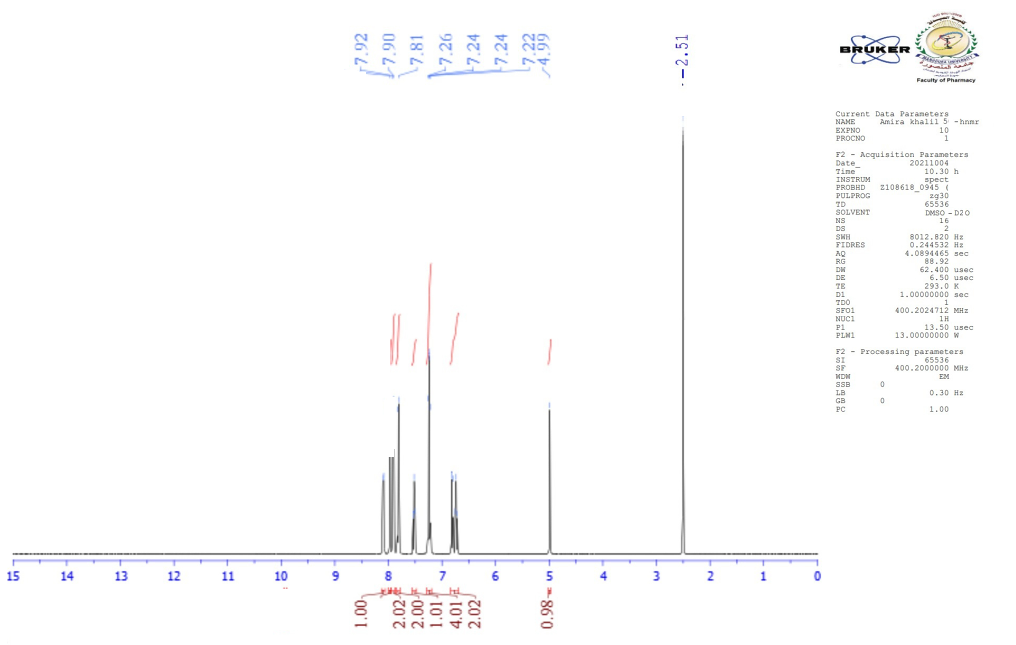


**Compound 5 ^13^C-NMR**

**
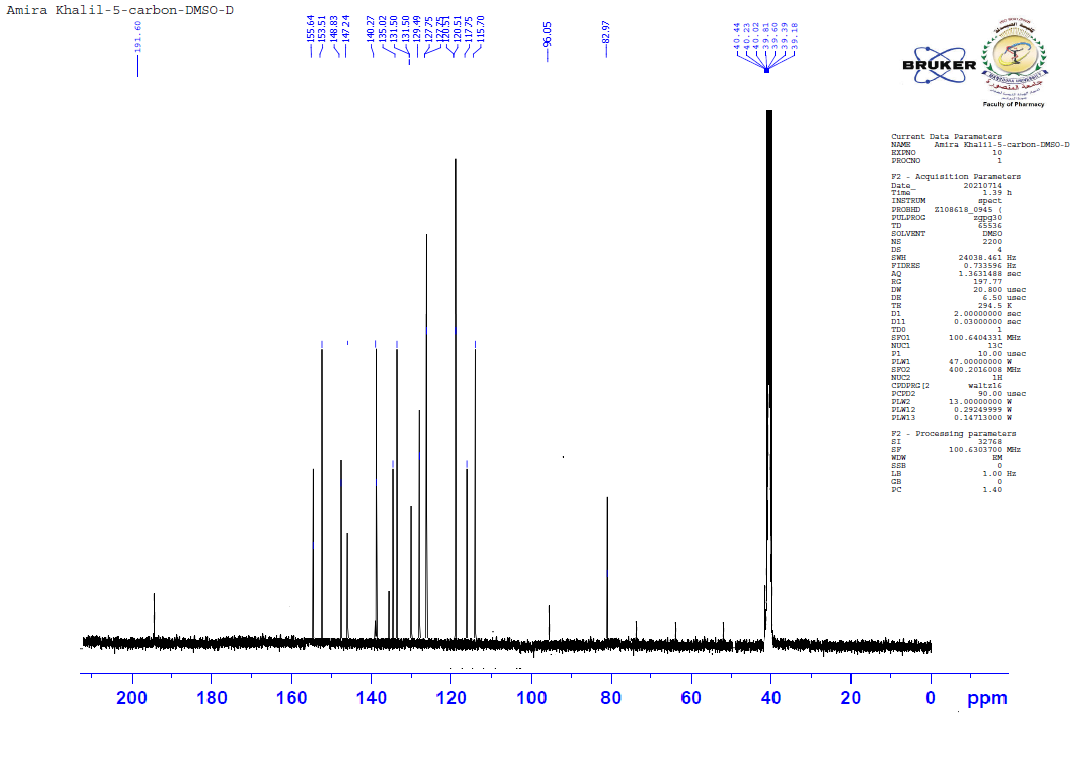
**

**Compound 6 ^1^H-NMR**

**
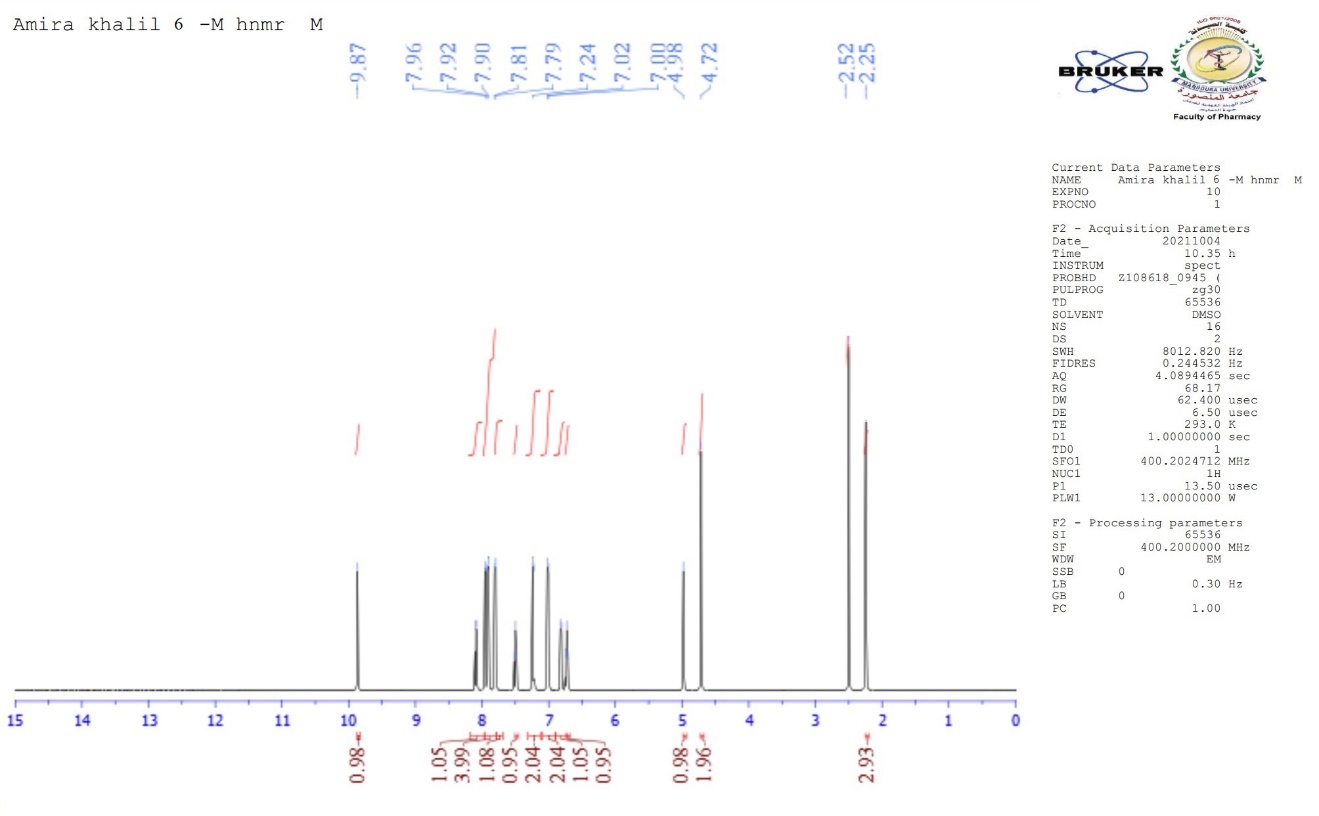
**

**
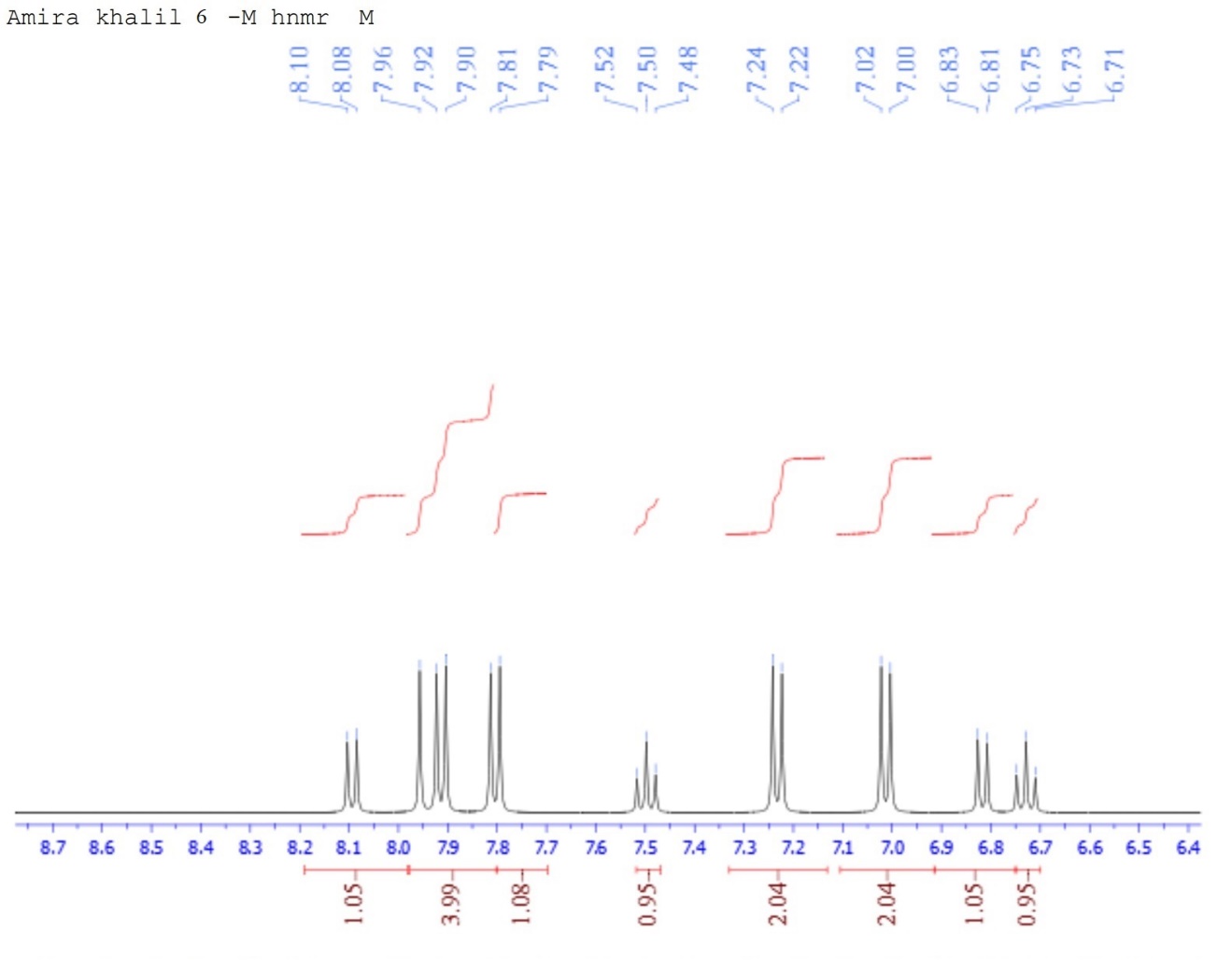
**

**Compound 6 ^1^H-NMR-D2O**


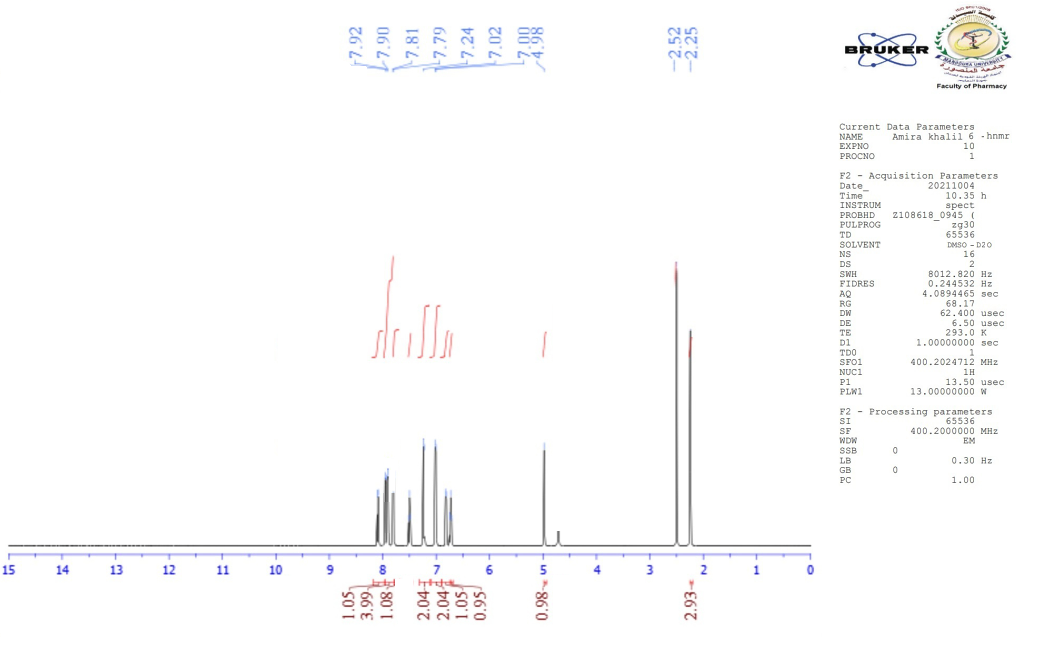


**Compound 6 ^13^C-NMR**


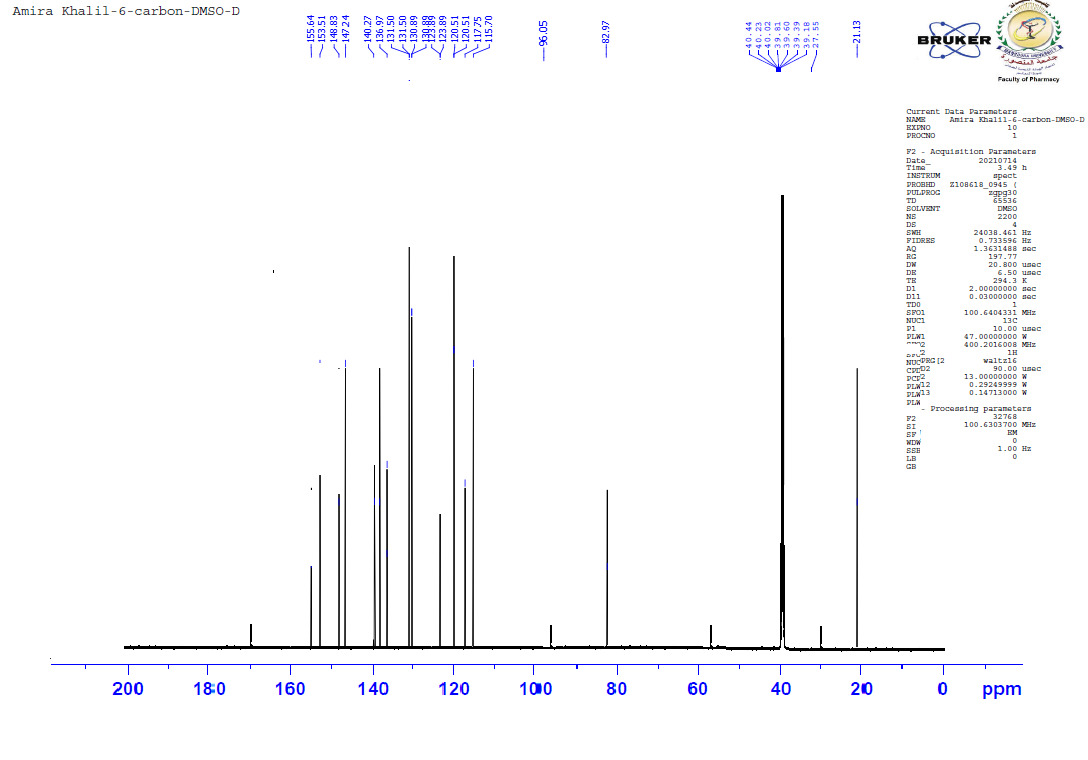


**Compound 7 ^1^H-NMR**


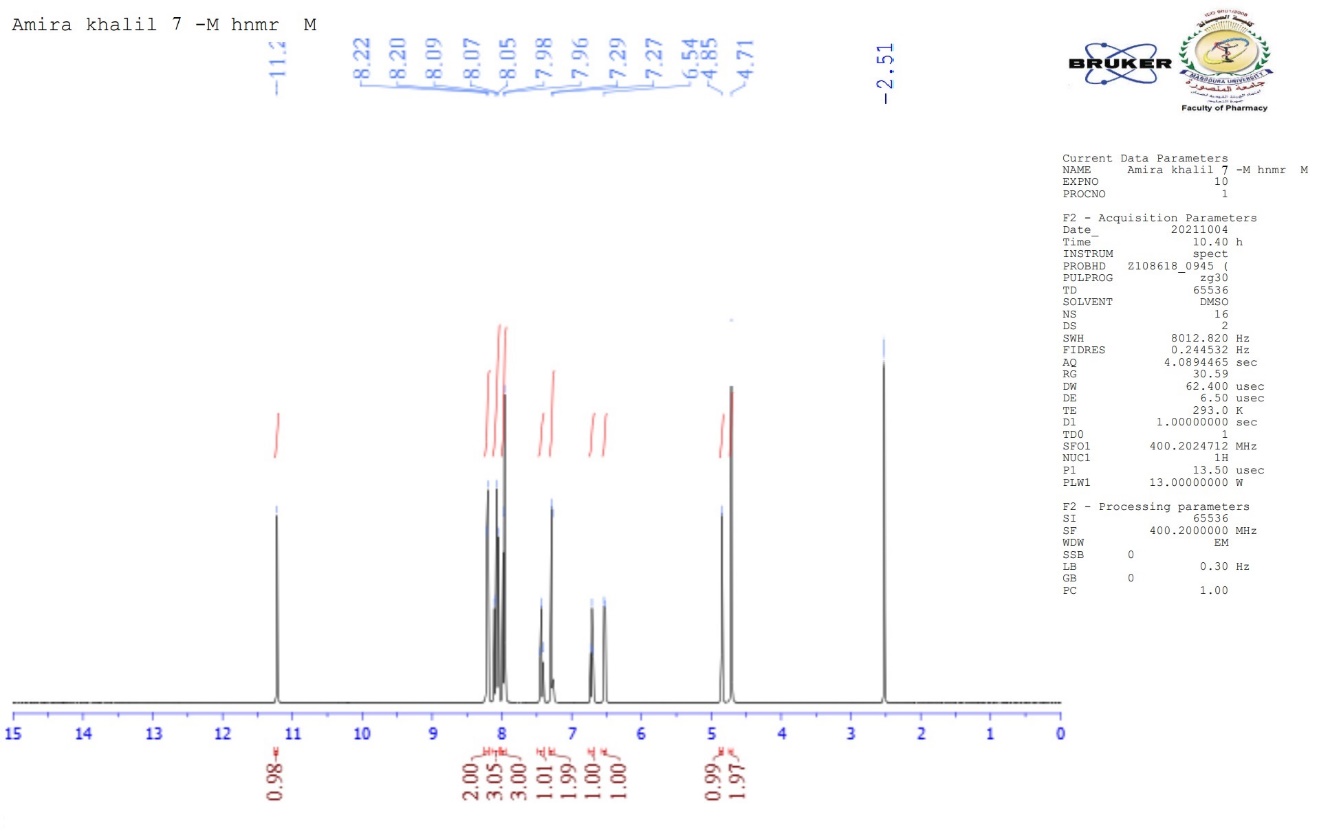


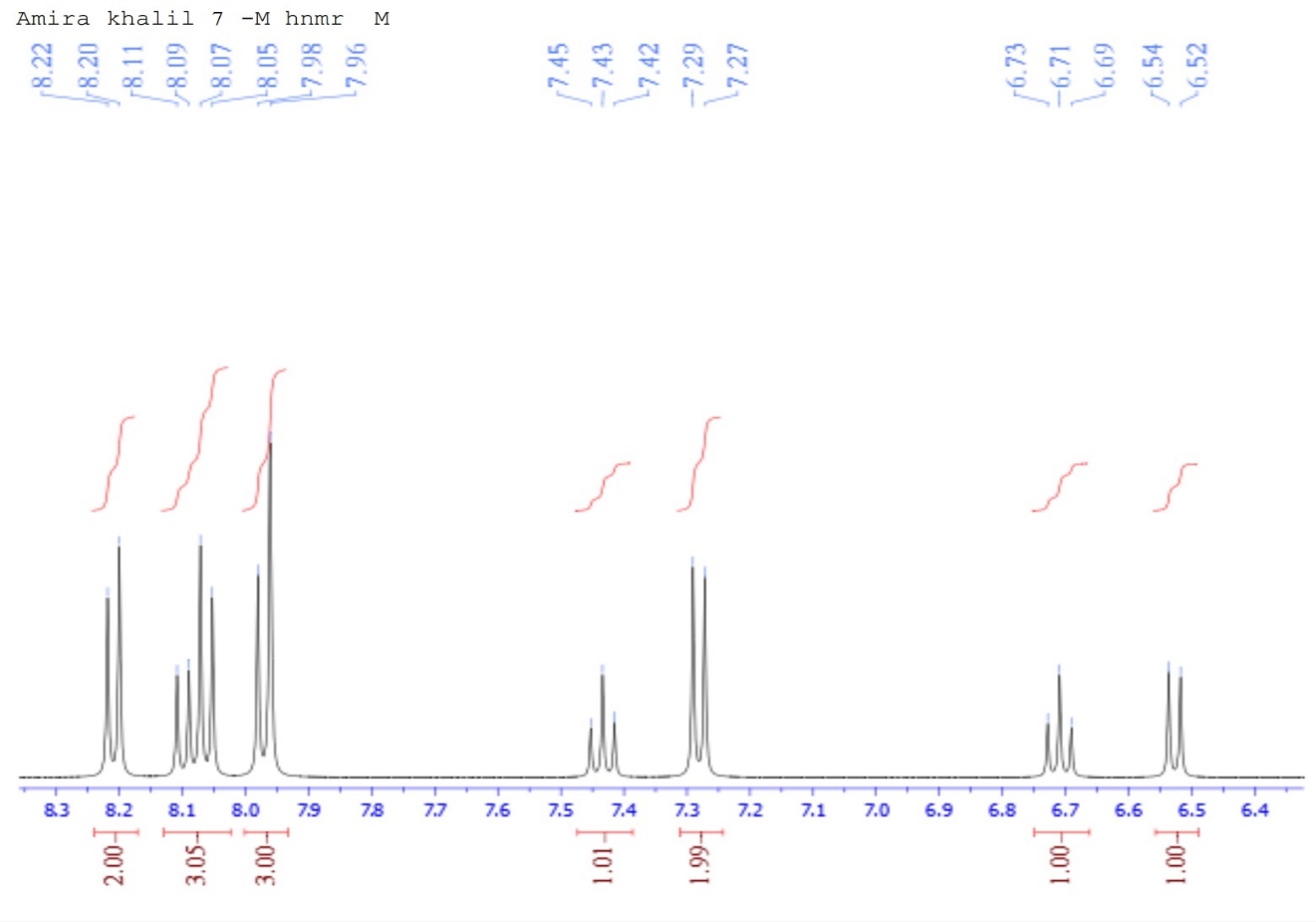


**Compound 7 ^1^H-NMR-D2O**


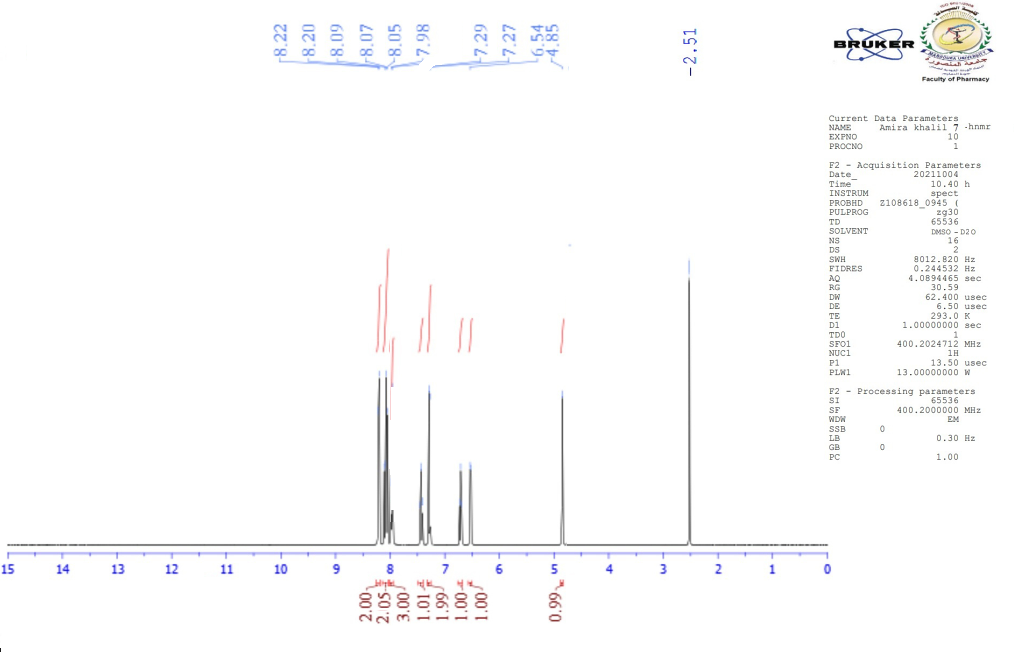


**Compound 7 ^13^C-NMR**


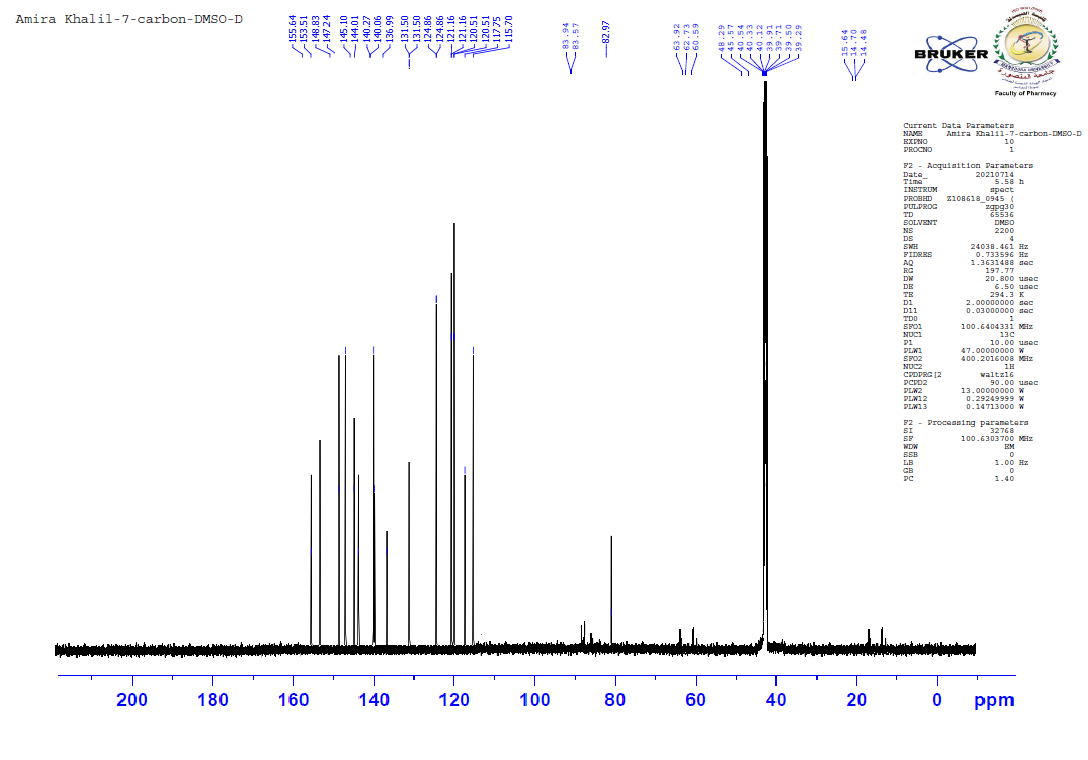


**Compound 8 ^1^H-NMR**

**
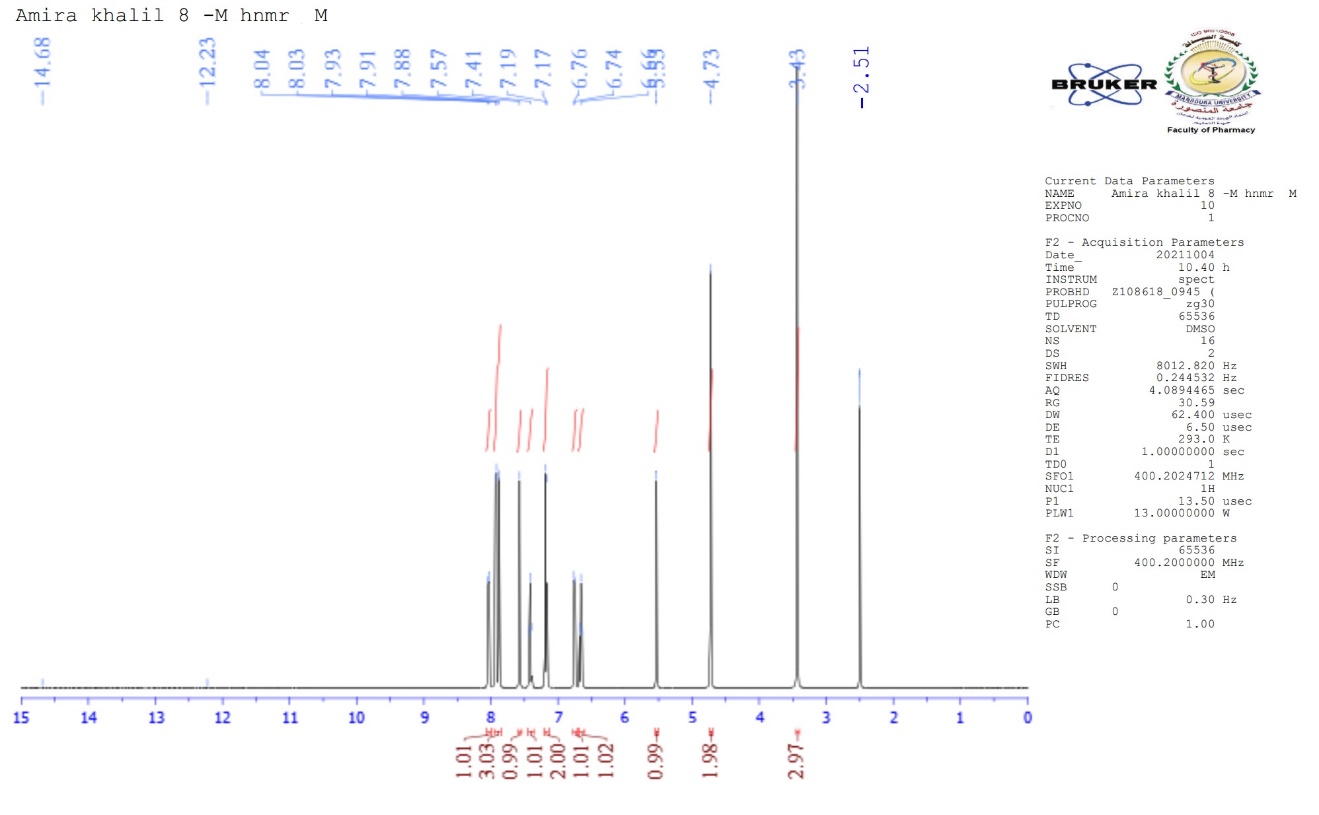
**

**
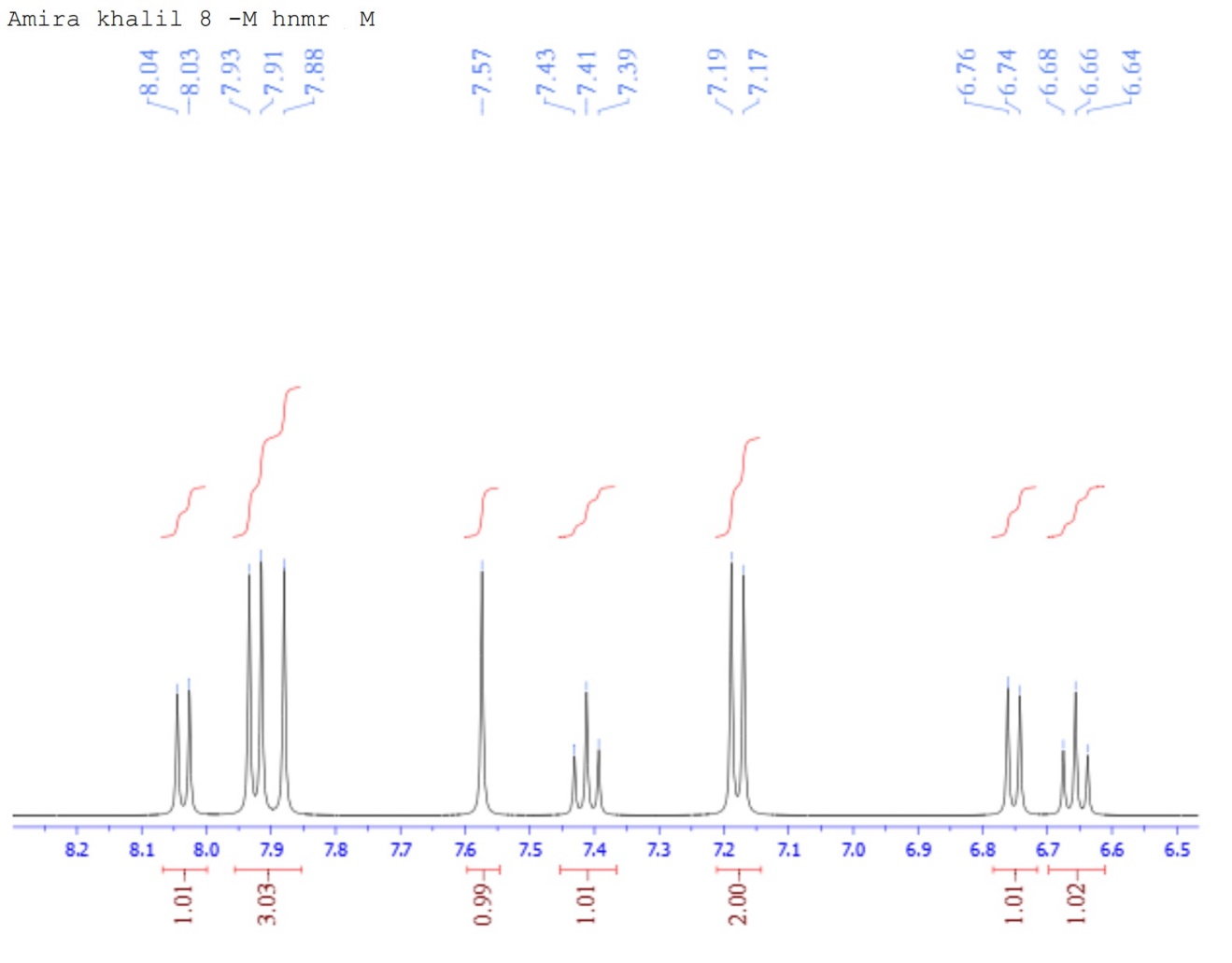
**

**Compound 8 ^1^H-NMR-D2O**


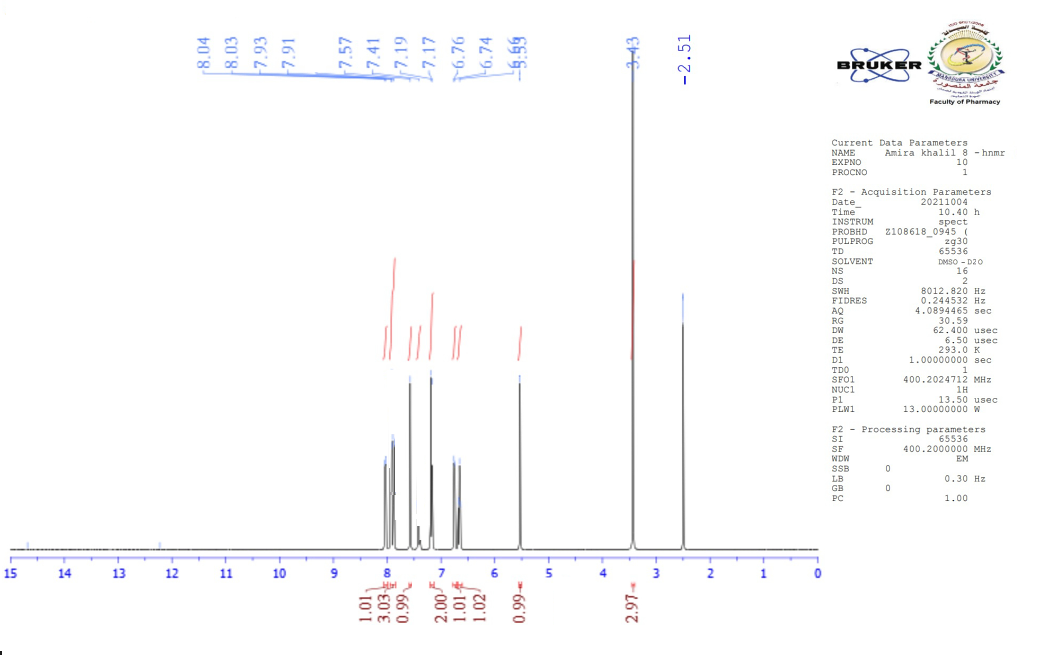


**Compound 8 ^13^C-NMR**


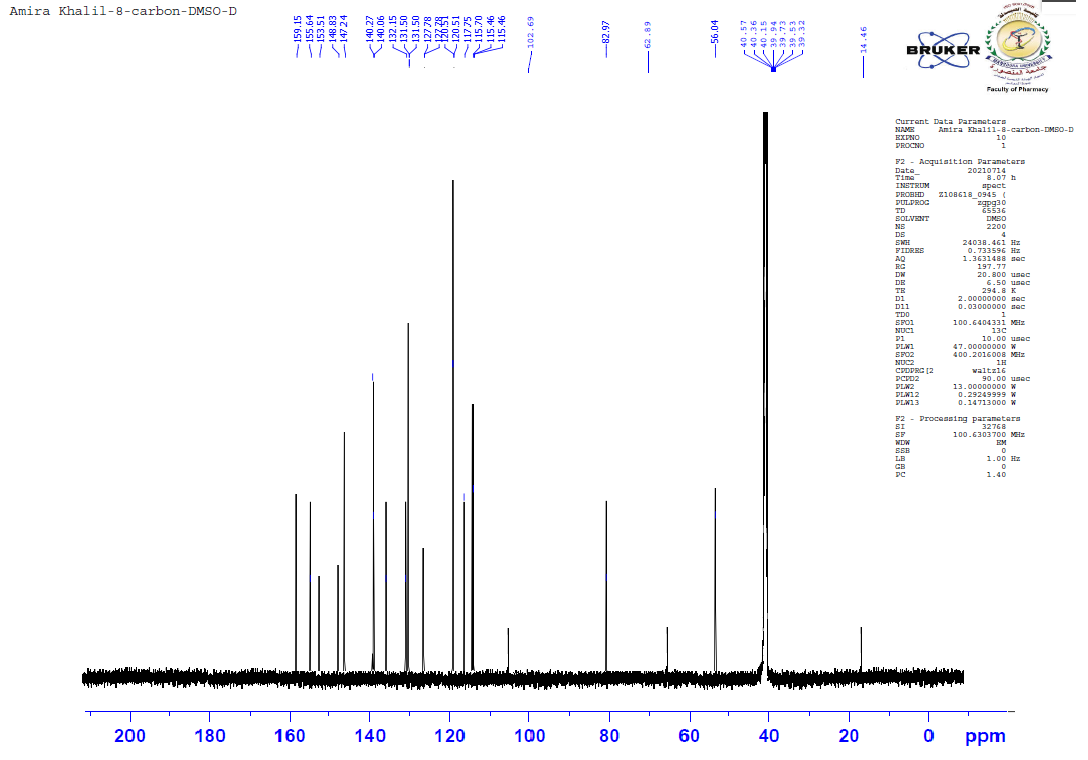


**Compound 9 ^1^H-NMR**


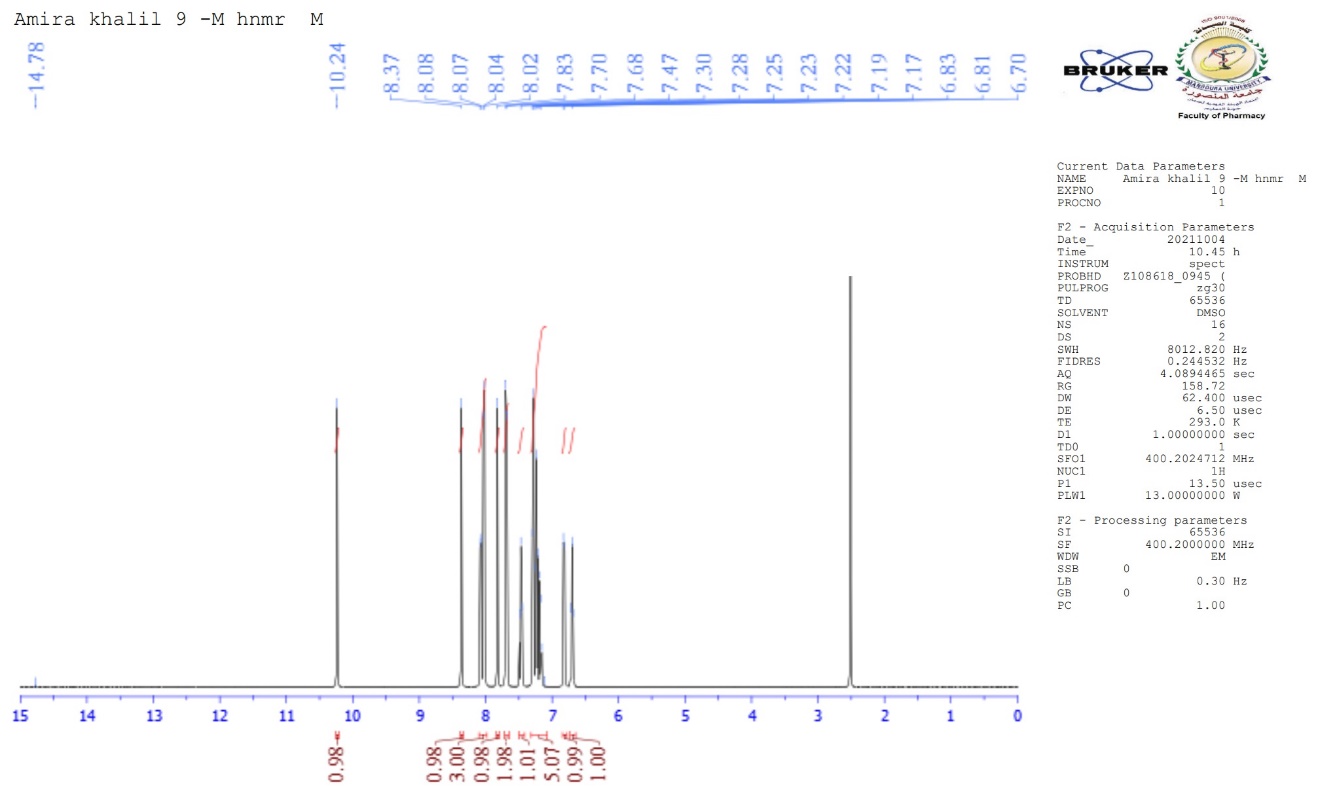


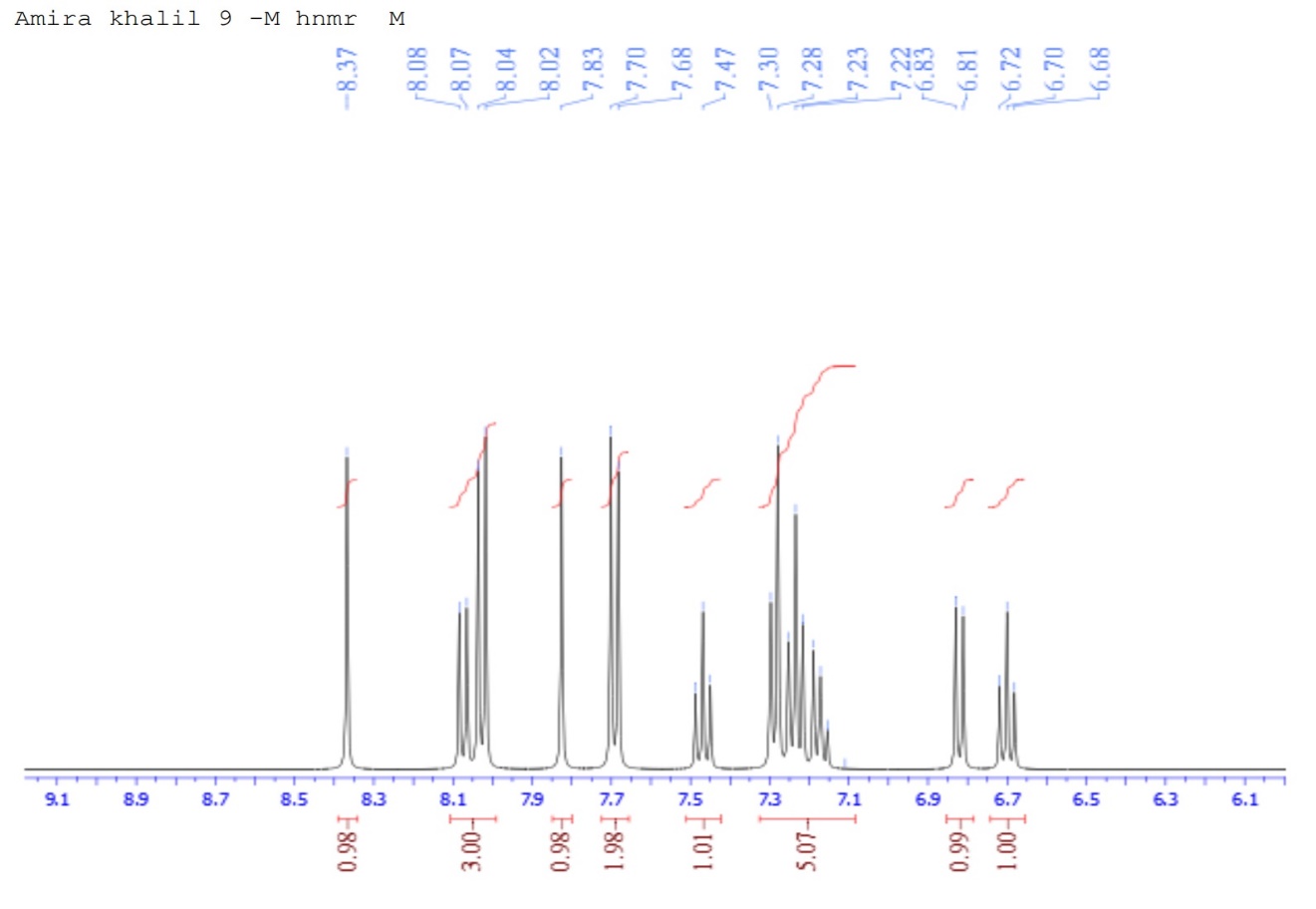


**Compound 9^1^ H-NMR-D2O**


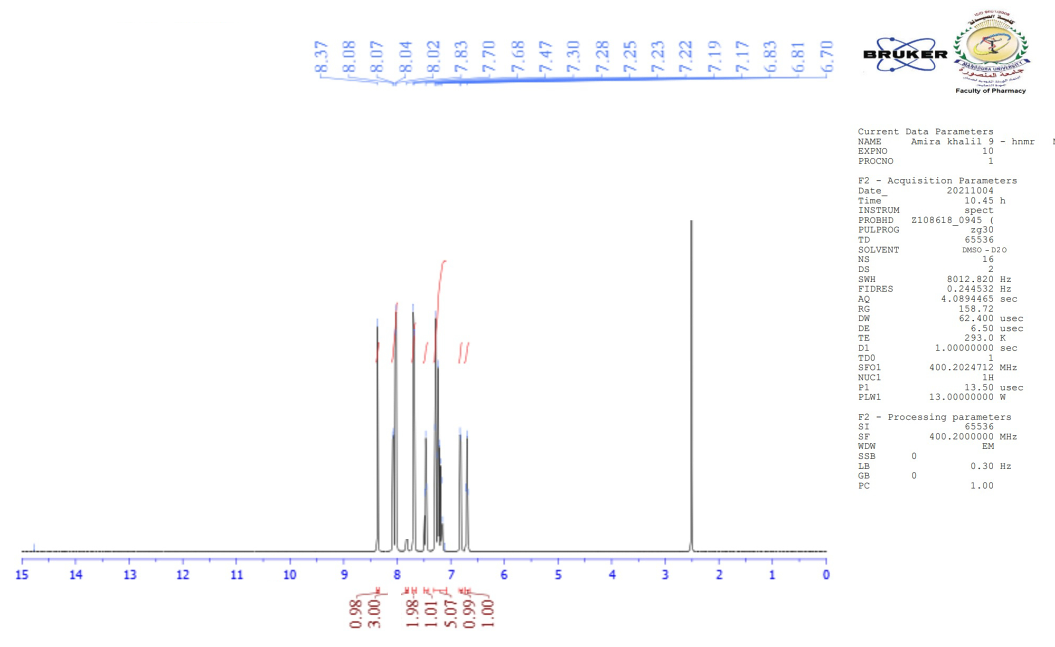


**Compound 9 ^13^C-NMR**

**
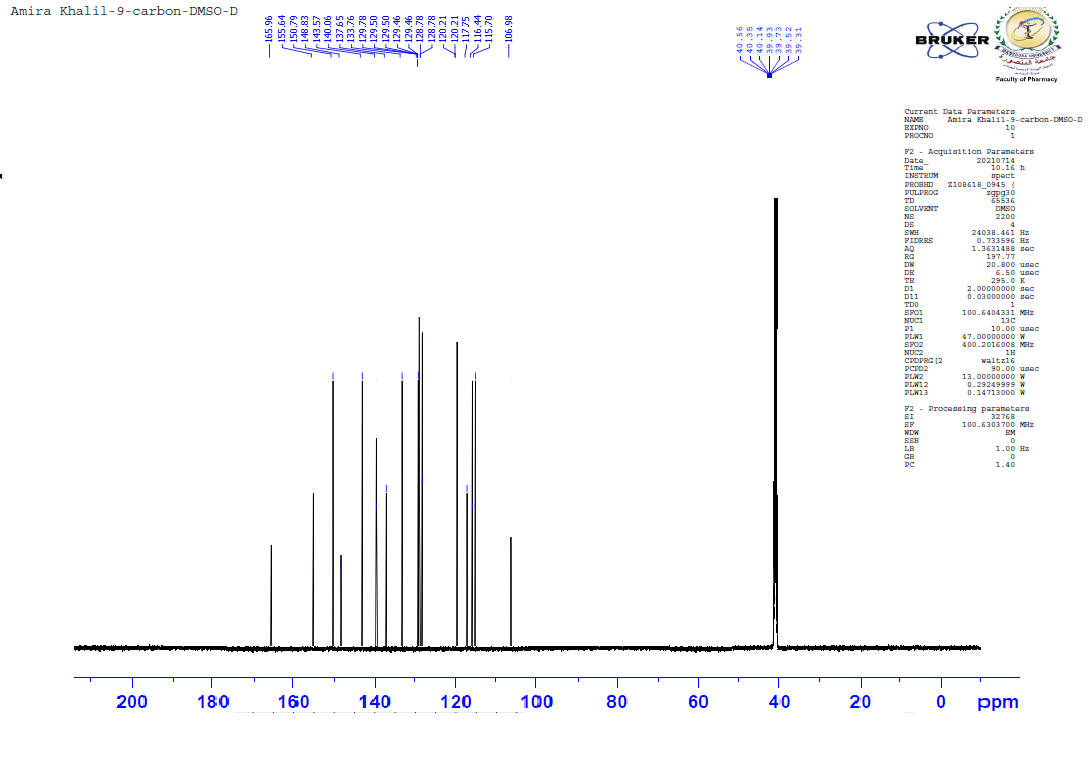
**

**Compound 10 ^1^H-NMR**


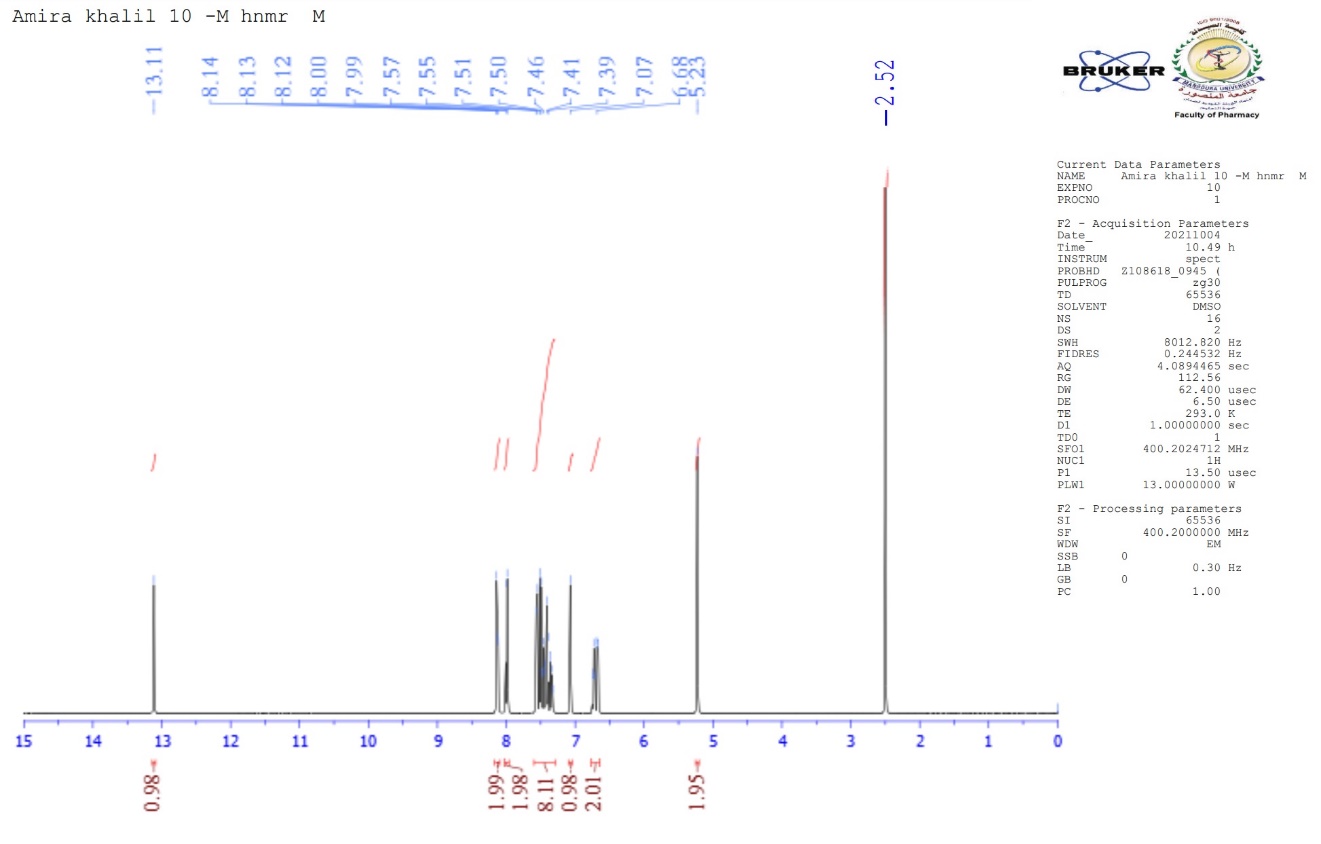


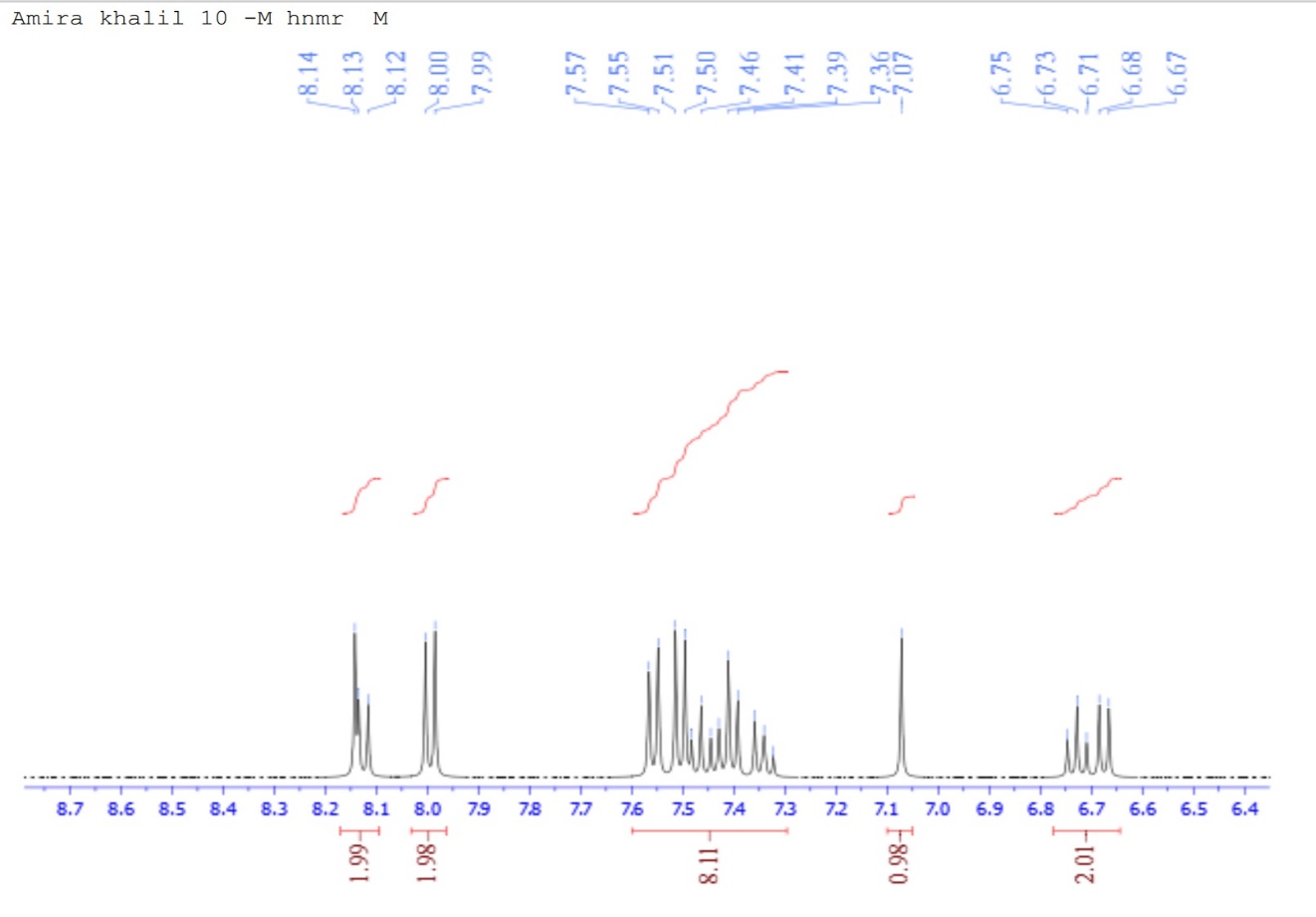


**Compound 10 ^1^H-NMR-D2O**


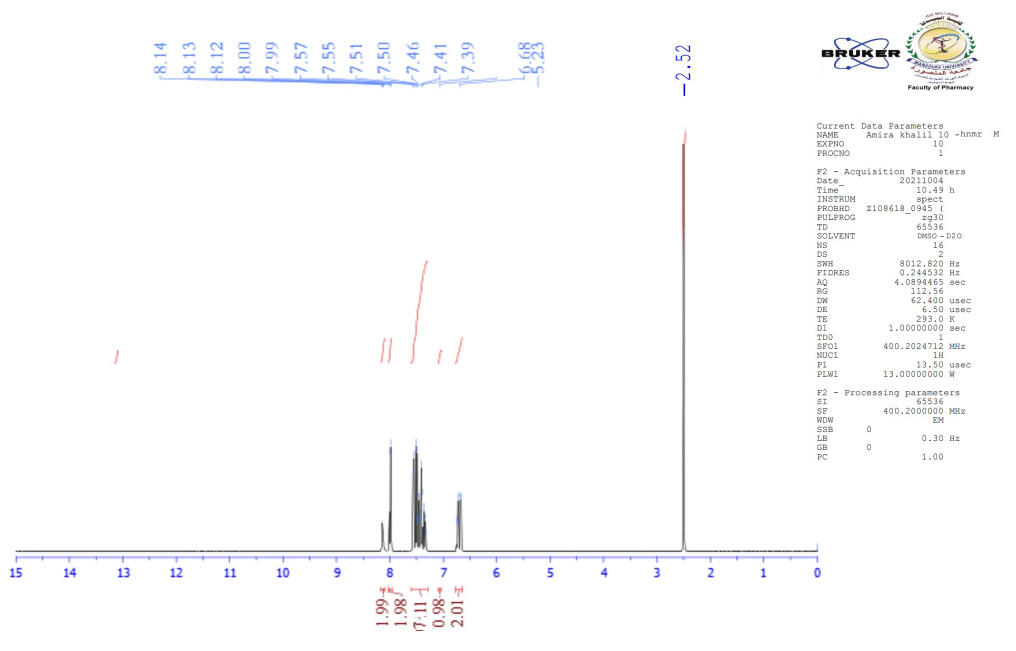


**Compound 10 ^13^C-NMR**


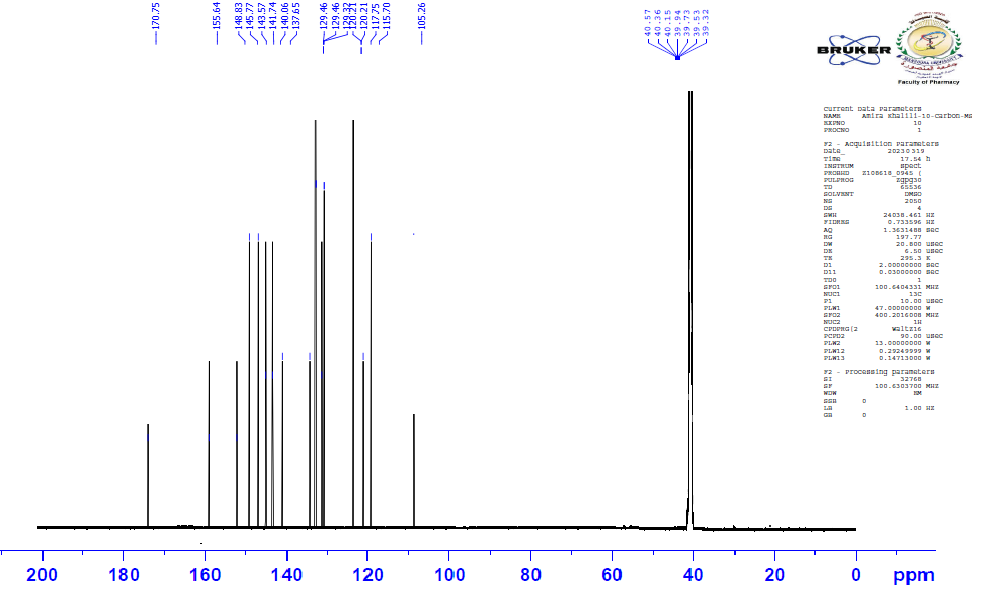


**Compound 11 ^1^H-NMR**

**Compound 11 ^1^H-NMR-D2O**

**Compound 11 ^13^C-NMR**

**Compound 12 ^1^H-NMR**

**Compound 12 ^1^H-NMR-D2O**

**Compound 12 ^13^C-NMR**

**Compound 13 ^1^H-NMR**

**Compound 13 ^1^H-NMR-D2O**

**Compound 13 ^13^C-NMR**

**Compound 14 ^1^H-NMR**

**Compound 14 ^1^H-NMR-D2O**

**Compound 14 ^13^C-NMR**

**Compound 15 ^1^H-NMR**

**Compound 15 ^1^H-NMR-D2O**

**Compound 15 ^13^C-NMR**

**Compound 16 ^1^H-NMR**

**Compound 16 ^1^H-NMR-D2O**

**Compound 16 ^13^C-NMR**
